# Epidemiology, diagnostics, treatments, and pregnancy outcomes of gestational diabetes mellitus in Africa: a systematic review, meta-analysis, and bibliometric study

**DOI:** 10.3389/fcdhc.2026.1795881

**Published:** 2026-05-04

**Authors:** Benjamin Aye Simon, Yasmeen Thandar, Nalini Govender, Francis Akpa-Inyang, Lawrence Chauke

**Affiliations:** 1 Department of Basic Medical Sciences, Faculty of Health Sciences, Durban University of Technology, Durban, South Africa; 2 Durban University of Technology, Durban, South Africa; 3 Department of Obstetrics and Gynaecology, Faculty of Health Sciences, The University of the Witwatersrand, Johannesburg, South Africa

**Keywords:** Africa, bibliometric analysis, diagnostic criteria, epidemiology/prevalence, gestational diabetes mellitus, maternal and child health outcomes, metabolic risk factors, systematic review

## Abstract

**Background:**

Gestational diabetes mellitus (GDM) is an increasingly important contributor to maternal and neonatal morbidity in Africa, yet the evidence base remains fragmented across settings and research domains. This study integrates systematic review and meta-analysis with bibliometric analysis to synthesize epidemiological evidence, characterize research activity, and examine risk factors, diagnostic practices, management strategies, and pregnancy outcomes related to GDM in Africa.

**Methods:**

Publications reporting on GDM in African populations were retrieved from major scholarly databases. Bibliometric techniques were applied to evaluate publication trends, collaboration networks, and thematic structures. In parallel, 241 studies from 33 countries were systematically reviewed. Study quality was assessed, demonstrating gradual improvement over time (Spearman’s ρ = 0.307). A random-effects meta-analysis was conducted to estimate pooled GDM prevalence.

**Results:**

Research output increased steadily over time but remained concentrated in a limited number of countries, with minimal regional collaboration. Dominant research themes focused on metabolic risk, diagnostic testing, and pregnancy outcomes, while health-systems and implementation research were comparatively scarce. Across 44 prevalence studies, the pooled GDM prevalence was 12.62% (95% CI: 10.23%–15.21%) using a Freeman-Tukey-transformed and DerSimonian-Laird random-effects model. Heterogeneity was considerable (I² = 98.4%), with a wide 95% prediction interval (1.18%–33.50%), indicating substantial contextual variability. Prevalence estimates ranged from 0.7% to 45.9%. Common risk factors included advanced maternal age, elevated body mass index, family history of diabetes, and prior obstetric complications. Diagnostic and management approaches varied widely, with increasing adoption of the 75 g OGTT and IADPSG/WHO 2013 criteria.

**Conclusions:**

GDM prevalence in Africa is substantial and highly heterogeneous, reflecting uneven diagnostic practices and research capacity. Strengthened regional collaboration, greater harmonization screening, and improved health-system integration are essential to inform effective and contextually appropriate maternal health policies.

## 1 Introduction

Gestational diabetes mellitus (GDM) has undergone continuous conceptual and clinical evolution since the 1960s, with changes in definitions, screening strategies, diagnostic criteria, prevalence estimates, and treatment approaches (1–3). GDM is broadly defined as glucose or carbohydrate intolerance first identified during pregnancy (1–5). Globally, GDM affects a substantial proportion of pregnancies, with estimates indicating that 16.9% of pregnant women worldwide – approximately one in six pregnancies – were affected in 2017 (6–8). Of these cases, 86.4% represented gestational diabetes, while 13.6% were attributed to pregestational diabetes (6–8). In sub-Saharan Africa, GDM prevalence is estimated at approximately 14% (9–12), while studies conducted in South Africa within the last seven years report prevalence rates ranging from 9.1% to 25.8% (13, 14).

There is extensive evidence demonstrating that GDM poses significant short- and long-term health risks for both mothers and infants (15–19). Elevated insulin resistance during pregnancy has been strongly associated with fetal macrosomia, typically defined as birth weight ≥4,000 g (16, 19). Pregnancy-related hormonal changes involving estrogen, prolactin, and placental lactogen contribute to insulin resistance, leading to maternal hyperglycemia and increased transfer of glucose from the mother to the fetus through the placenta (16, 17). This metabolic environment promotes excessive fetal growth and adverse perinatal outcomes. Maternal complications associated with GDM include hypertension, cardiovascular disease, stroke, and vascular dysfunction (18).

Several maternal and environmental risk factors for GDM have been identified, including advanced maternal age, high parity, family history of diabetes, and a prior history of GDM (20–22). Lifestyle-related factors – such as obesity, poor dietary patterns, sedentary behavior, and complications from previous pregnancies, including macrosomia and high birth weight – further increase susceptibility to GDM (20–22).

Beyond immediate perinatal risks, maternal obesity and GDM have enduring intergenerational consequences. For example, fetal metabolic programming has been shown to predispose offspring to obesity, insulin resistance, and metabolic syndrome later in life (23, 24). Numerous studies report a strong association between maternal obesity, GDM, and macrosomia, with macrosomia itself linked to neonatal obesity and long-term metabolic disorders (19, 25–37). A population-based analysis in China identified maternal obesity and GDM as the most prevalent risk factors for macrosomia, with an overall prevalence of 8.7% (19). Infants with birth weights ≥4,000 g demonstrated significantly higher risks of obstetric and neonatal complications (19).

Maternal obesity and GDM independently and synergistically increase rates of macrosomia, neonatal intensive care admissions, and adverse pregnancy outcomes (26). Pre-pregnancy obesity has also been strongly linked to obesity in offspring later in life (26). If inadequately managed, GDM and obesity contribute to future metabolic disorders in both mothers and children, including diabetes and other metabolic dysfunctions (27, 32). Collectively, these findings highlight a self-perpetuating cycle whereby maternal obesity and diabetes increase the risk of metabolic disease in subsequent generations (26, 27, 31). Effective glycemic control before and during pregnancy remains a critical intervention to reduce the prevalence and consequences of macrosomia.

Early detection and appropriate management of GDM are essential for improving maternal and neonatal outcomes. Universal screening using the oral glucose tolerance test (OGTT) between 24- and 28-weeks’ gestation is widely accepted as the diagnostic gold standard (5). However, due to cost, logistical challenges, and limited healthcare infrastructure, universal OGTT screening is often impractical in low- and middle-income countries, including many African settings (5). Consequently, selective screening based on risk factors is commonly employed. Despite GDM being one of the most frequent pregnancy-related complications, its epidemiology remains insufficiently characterized, largely due to variability in diagnostic criteria (38).

Over time, multiple diagnostic frameworks have been applied, including those from the National Diabetes Data Group (1997) (39), the World Health Organization (1985, 1998) (40, 41), the American Diabetes Association (2004, 2010, 2014, 2020) (42–46), the International Association of Diabetes and Pregnancy Study Groups (2010) (5), and the National Institutes of Health (2013) (46). Albeit these methodological inconsistencies have substantially influenced reported prevalence estimates across regions and time periods, thereby obscuring true temporal and geographic trends in GDM.

Given the rising prevalence of obesity (37, 47–52), diabetes (10, 53, 54), and associated comorbidities (55), there is a critical need to systematically evaluate GDM research in Africa. This study aims to synthesize existing knowledge on GDM in Africa using bibliometric analysis combined with a systematic literature review. Bibliometric and scientometric approaches provide robust tools for evaluating research trends, interdisciplinary engagement, and scientific impact. When integrated with systematic review methods and meta-analysis, they enable comprehensive assessment of evidence related to GDM risk factors, prevention strategies, diagnostic approaches, treatment modalities, and pregnancy outcomes in Africa. Furthermore, these methods can inform policy and clinical practice by identifying gaps in healthcare delivery and research capacity. To date, no comprehensive bibliometric or scientometric studies have examined GDM research from an African perspective. The absence of accessible bibliometric guidance specific to diabetes research in Africa presents a barrier for researchers seeking to apply these methodologies, underscoring the importance of this study.

Such analyses are essential for mapping the intellectual structure of the field, assessing research productivity and collaboration, identifying knowledge gaps, and guiding future research directions. Accordingly, this research addresses the following questions:

- Which research networks are most active in GDM-related research in Africa?
- What is the level of technological, infrastructural, and resource readiness of proposed healthcare solutions?
- What are the dominant research themes related to GDM risk factors, prevention, diagnostics, treatment, and adverse pregnancy outcomes in Africa?

**Figure 1:**
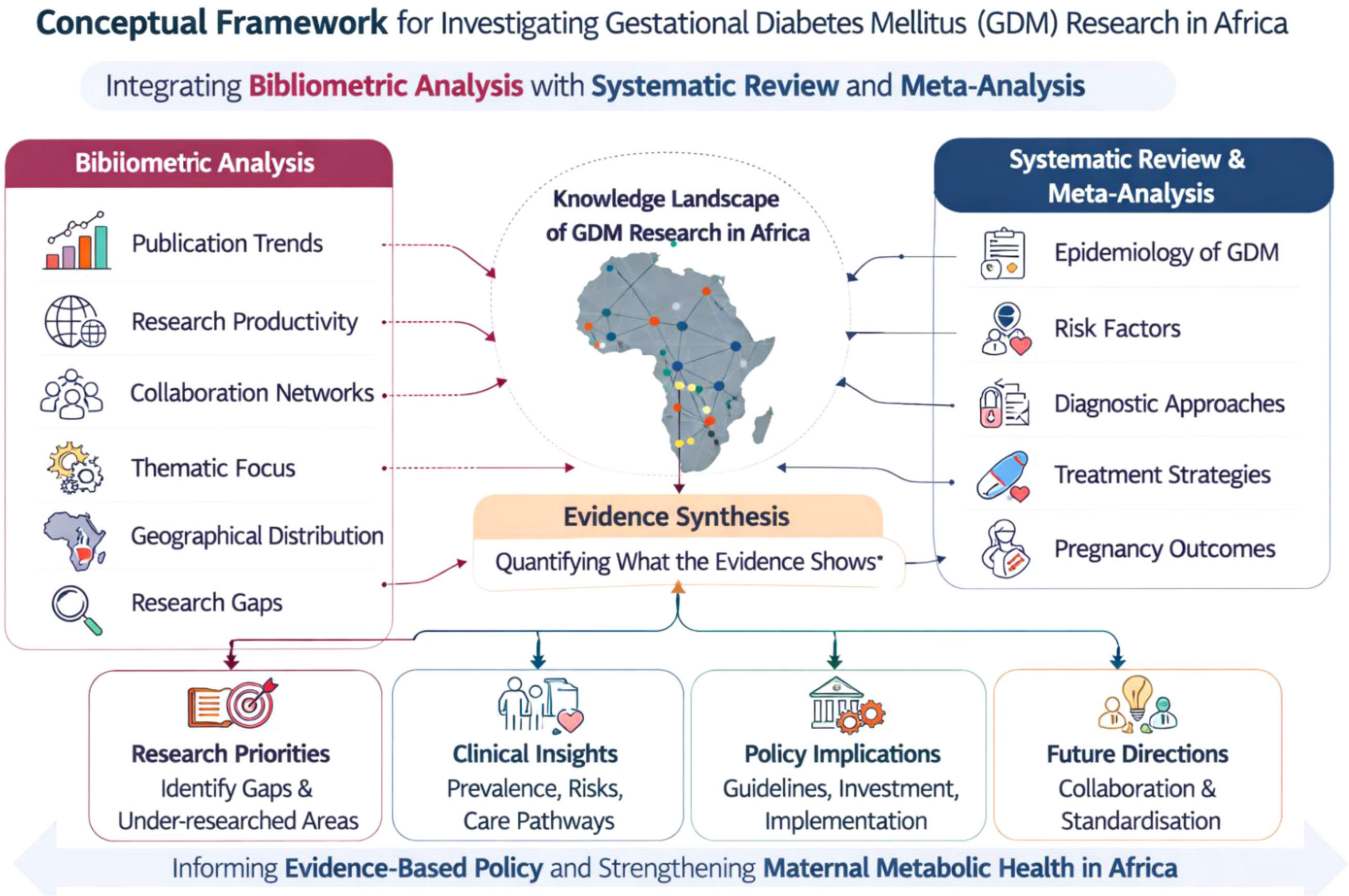
Integrated conceptual framework for analyzing GDM research in Africa.

As such, in order to provide a comprehensive understanding of GDM research in Africa, this study adopts an integrated conceptual framework that combines bibliometric analysis with a systematic review and meta-analysis of clinical evidence (see **Figure 1**). The bibliometric component is used to map the evolution, distribution, and thematic focus of GDM research across the African continent, including publication trends, research productivity, collaboration networks, and dominant research themes. This mapping serves two key purposes: first, it contextualizes the growth and geographical distribution of the scientific literature on GDM in Africa; and second, it helps identify research concentrations, gaps, and underrepresented regions or topics that may influence the available clinical evidence base. Building on this contextual foundation, the systematic review and meta-analysis synthesize empirical findings from eligible studies to quantify the epidemiology of GDM and examine associated risk factors, diagnostic approaches, treatment strategies, and pregnancy outcomes in African populations. In this framework, the bibliometric analysis functions as a macro-level assessment of the knowledge landscape, while the clinical synthesis provides a micro-level evaluation of the evidence informing clinical practices and public health policies. By integrating these complementary approaches, the study not only quantifies disease prevalence and clinical determinants but also situates these findings within the broader trajectory and structure of GDM research in Africa. This dual analytical strategy therefore enables a more holistic understanding of both what the evidence shows and how the evidence-based itself has developed, thereby informing future research priorities, methodological standardization, and policy-relevant knowledge production in the field of maternal metabolic health in Africa.

## 2 Research methods

### 2.1 Studies search strategy: bibliometric and systematic review data collection and screening

A comprehensive literature search was conducted based on a compiled dataset of bibliographic records. These records were derived primarily from the Web of Science and Scopus databases, identifying 1,209 records from the initial search. The Web of Science and Scopus databases were selected because of their wide recognition as leading multidisciplinary citation indexes with strict journal selection criteria, that ensures scholarly quality, reliability, and consistent bibliographic metadata. Additionally, both databases provide extensive citation coverage and structured indexing suitable for both bibliometric analysis and systematic evidence synthesis.

To ensure the completeness of the search and prevent the omission of relevant studies, an additional verification search was conducted during the initial data collection phase using PubMed and Google Scholar, implemented through Harzing’s Publish or Perish software. The same core search terms applied in the primary database search were used in these supplementary searches. This verification process confirmed that the peer-reviewed journal articles identified in PubMed and Google Scholar were already indexed within either Web of Science or Scopus, indicating substantial overlap in database coverage. The few additional records retrieved through Google Scholar primarily consisted of doctoral dissertations, master’s theses, conference materials, or other forms of gray literature, which did not meet the predefined inclusion criteria of the review that focused on original articles or peer-reviewed journal articles. Consequently, the Web of Science and Scopus searches were considered sufficient to capture the eligible literature included in this study.

**Figure 2:**
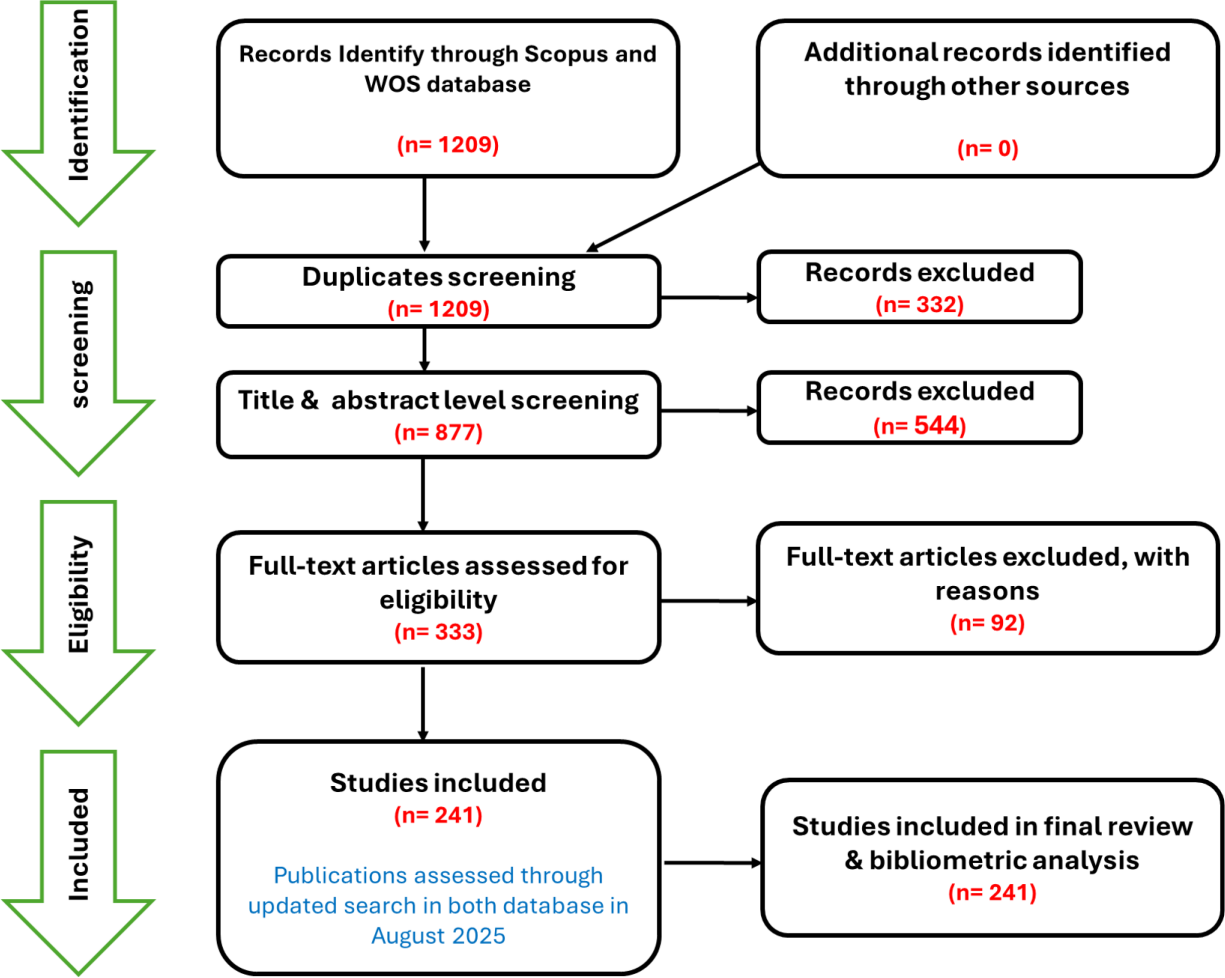
**PRISMA**-based workflow for the combined bibliometric and systematic review process.

Following the initial search, a structured screening process was applied to ensure inclusion of studies that met predefined eligibility criteria. Duplicate records were removed with the assistance of R (Bibliometrix package) and reference management software (Zotero: version 7.0). The screening included title review, abstract review, and full-text assessment to determine eligibility. The flow of records through search, screening, and eligibility stages is illustrated in the PRISMA diagram (**Figure 2**), showing the final 241 studies included for both bibliometric and systematic review analyses. The final dataset consisted of studies published between 1999 and 2024. These publications covered a wide range of topics including epidemiology, clinical outcomes, and public health aspects of gestational diabetes mellitus (GDM) in African populations. Thereafter, the methodological quality of all included studies was subsequently evaluated using design-appropriate appraisal tools, and the detailed scoring outcomes for each study are presented in (**Supplementary Table 1**).

The search strategy used both controlled vocabulary and unrestricted keyword terms. Examples of keywords include epidemiology, gestational diabetes mellitus, gestational diabetes, hyperglycemia, risk factors, predictors, health implications, diagnostics, health complications, diagnoses, testing, treatments, management, adverse pregnancy outcomes, Africa, and Sub-Saharan Africa. Boolean operators such as AND, OR, and NOT were applied to refine results. A language filter was applied to include only studies published in English as English is the primary language of proficiency of the research team. This language inclusion enabled accurate interpretation, extraction, and synthesis of study findings. Including publications written in other languages – such as those from Francophone or Lusophone African countries – would introduce a risk of misinterpretation or incomplete analysis because the authors would not be able to account for their content with the same level of accuracy.

### 2.2 Eligibility criteria

Only studies that examined pregnant women in African settings and presented primary data on GDM were considered eligible. This included research reporting on prevalence, associated risk factors, diagnostic approaches, treatment practices, or maternal and newborn outcomes. Both observational and interventional designs published between 1999 and 2024 were considered. Studies were excluded if conducted outside Africa, did not provide primary empirical data, or consisted solely of commentaries, reviews, and/or animal studies. A full description of the inclusion and exclusion criteria is provided in **Table 1**.

**Table 1:** Inclusion and exclusion criteria for both bibliometric, meta-analysis, and systematic review.

Category	Inclusion criteria	Exclusion criteria
Study Type	Peer-reviewed and non-peer-reviewed journal articles	Conference abstracts, editorials, commentaries, letters, thesis/dissertations
Study Focus	Studies explicitly examining gestational diabetes mellitus (GDM) within African countries	Studies not focused on GDM or not related to Africa
Content Type	Articles containing relevant bibliographic metadata (authors, affiliations, keywords, abstracts, citations)	Articles lacking essential bibliographic data
Language	Articles published in English	Articles published in non-English languages
Timeframe	Articles published within the selected study period (1999-2024)	Articles published outside the defined time period
Document Type in Databases	Original research relevant to GDM	Reviews, systematic reviews, meta-analyses, retracted papers, non-research materials (book chapters, notes, corrections)
Availability	Full text or metadata accessible via Scopus or Web of Science	Articles not retrievable through selected databases
Population	Studies involving human populations related to GDM	Animal studies or laboratory-only studies not connected to human GDM

### 2.3 Bibliometric analysis

The bibliometric component drew upon records retrieved from the Web of Science and Scopus databases, exported in BibTeX format and analyzed in RStudio using the Bibliometrix package and its interactive interface, Biblioshiny. These tools enabled extensive investigation of publication activity, citation patterns, authorship linkages, and thematic development within the field. Visual representations, including co-occurrence networks, thematic structures, and collaboration maps, assisted in identifying the intellectual landscape and prevailing research directions. The Bibliometrix and Biblioshiny thus provided a flexible, open-source, reproducible platform for examining, visualizing, and interpreting the bibliometric data, supporting identification of key publications, prominent authors, collaborative patterns, frequently used keywords, and thematic developments. The Bibliometrix and Biblioshiny are open access, open-source tools distributed under the MIT License. Their application in this study enabled the recognition of key publications, prominent authors, collaborative patterns, frequently used keywords, and thematic developments within the selected domain (56).

### 2.4 Meta-analysis and systematic review data analysis

The systematic review and meta-analysis provided a deeper qualitative perspective and strengthened the evidence-base. The review process followed the PRISMA framework, including formulation of a focused research question, application of predefined inclusion and exclusion criteria, and screening of titles, abstracts, and full texts to determine eligibility. Relevant information was extracted from studies meeting the inclusion criteria, and a narrative synthesis was prepared, supported by thematic grouping of findings. Methodological quality was assessed as appropriate. The PRISMA flow diagram is presented in **Figure 2**, illustrating the screening stages and final inclusion of 241 studies.

#### 2.4.1 Quality assessment: statistical procedures for quality assessment

Methodological quality and risk of bias were evaluated using established appraisal tools appropriate to study design: cohort and case-control studies with the Newcastle-Ottawa Scale (NOS), cross-sectional studies with the Joanna Briggs Institute (JBI) checklist, and randomized or quasi-experimental studies with the Cochrane Risk of Bias (RoB) tool. Articles with incomplete or ambiguous methodology were conservatively assessed using the JBI checklist. Scores were standardized (0–9 for NOS, 0–8 for JBI, 0–7 for Cochrane RoB). Overall, methodological quality was moderate, with common limitations including varying diagnostic criteria, small sample sizes, single-institution studies, inconsistent confounder adjustment, limited follow-up, and incomplete lab documentation. Most studies reached mid-level quality scores, supporting inclusion in the systematic review (**Supplementary Table 1**). Overall, the methodological quality of the included studies was moderate, with variation across study designs. Among the 42 cohort studies assessed using the Newcastle-Ottawa Scale (NOS), scores ranged from 4 to 9, with a median score of 7, indicating generally moderate to high methodological quality, although several studies lacked comprehensive adjustment for potential confounders. The 15 case-control studies assessed with the same instrument scored between 3 and 8 (median score = 6), with selection bias and incomplete exposure assessment representing the most common limitations.

The majority of included studies were cross-sectional or prevalence studies (n = 156) assessed using the Joanna Briggs Institute (JBI) checklist, which produced scores ranging from 3 to 8 with a median score of 6, reflecting moderate methodological quality overall. The most frequent limitations in these studies included hospital-based sampling, non-standardized diagnostic criteria, and limited adjustment for potential confounders. For quasi-experimental or intervention studies (n = 8) evaluated using the Cochrane Risk of Bias tool, scores ranged from 3 to 7 (median score = 5), with the most common sources of bias being lack of blinding and incomplete reporting of allocation procedures. An additional 20 studies with insufficient methodological detail or unclear study design were conservatively evaluated using the JBI checklist, yielding lower scores ranging from 2 to 6 (median score = 4), reflecting low to moderate methodological quality due to reporting limitations.

Sensitivity analyses were conducted to evaluate the influence of methodological quality on the synthesis by excluding studies below predefined quality thresholds (NOS < 5 and JBI < 4). The exclusion of these lower-quality studies resulted in minimal changes to the pooled prevalence estimates and did not materially alter the direction or interpretation of the key findings. This suggests that the principal epidemiological conclusions regarding GDM prevalence patterns, risk factors, and diagnostic variability were robust to the exclusion of studies with lower methodological scores. Overall, while methodological limitations were present in some studies, the predominantly moderate quality of the evidence base supports the inclusion of these studies in the systematic synthesis.

**Statistical Analysis with R Integration**: All statistical procedures were carried out in R (version 4.5.2). Data management relied on the *tidyverse* suite, while the *metafor*, *meta*, *psych*, and *irr* packages supported reliability testing, comparative analyses, and correlation modeling. The analyses were designed to describe the characteristics of the included studies, quantify methodological quality, and investigate the relationships between study features and indicators of risk of bias. Quality assessment scores derived from the NOS, the JBI checklist, and the Cochrane RoB tool were treated as variables with ordered but continuous properties, consistent with conventions in epidemiologic research. Descriptive analyses summarized study characteristics and quality scores using frequencies, medians, minimum and maximum values (**Supplementary Table 1**), and visualized distributions with histograms, violin plots, and boxplots (**Supplementary Figures 1**–**3**).

**Inter-Rater Reliability**: For records assessed by two independent reviewers, agreement on quality scoring was examined using established R functions. Weighted Cohen Kappa (κw), estimated through *kappam.light* in the *irr* package, was used for ordered scales such as the NOS and the JBI checklist. Intraclass correlation coefficients (ICC) were calculated using a two-way random effects model in the psych package to evaluate absolute agreement for continuous summary scores. Interpretation of κ and ICC values followed the benchmarks proposed by Landis and Koch, where values of κ or ICC ≥ 0.75 suggest strong agreement.

**Comparison Across Study Designs:** We used non-parametric tests (Kruskal-Wallis and Mann-Whitney U with Bonferroni adjustment) due to ordinal and non-normal distributions. These analyses were conducted to determine whether methodological quality varied between observational and interventional research.

**Associations with Study Characteristics** (publication year, sample size, diagnostic criteria, region) were analyzed with Spearman correlation, chi-square tests, and exploratory regression. Effect sizes with 95% CIs were computed using *rstatix*, *boot*, and *effectsize* packages with bootstrapping (2000 iterations) to account for score distributions. Inter-rater reliability metrics (weighted κ and ICC) were each reported with their respective 95% CIs. All confidence intervals were generated using a two-sided α = 0.05. For example, a positive correlation was observed between publication year and quality score (Spearman 0.307, 95% CI 0.071–0.498).

**Sensitivity and Robustness Analyses** included exclusion of low-quality studies (NOS < 5, JBI < 4), stratified analyses by quality category, and influence plots/leave-one-out diagnostics, ensuring that conclusions were not disproportionately influenced by weaker studies. In other words, these sensitivity and robustness analyses were carried out to determine whether methodological quality influenced the overall findings of the systematic review. These sensitivity analyses were carried out using R (R-based sensitivity analyses).

A random-effects meta-analysis using the DerSimonian-Laird estimator was conducted to account for the substantial heterogeneity anticipated across studies due to differences in diagnostic criteria, screening strategies, and study populations across African settings. Prevalence proportions were stabilized using the Freeman-Tukey double arcsine transformation, which is commonly applied in meta-analyses of proportions to reduce variance instability, particularly when studies report very low or very high prevalence values. Although alternative estimators such as the Hartung-Knapp adjustment can provide more conservative confidence intervals under conditions of high heterogeneity, its application may produce excessively wide or unstable intervals when studies are highly heterogeneous in design, diagnostic definitions, and population characteristics – as is the case in African GDM research. For this reason, the DerSimonian-Laird approach was retained, as the primary objective of the meta-analysis was to illustrate the extent of variability in reported GDM prevalence across studies rather than to generate a precise pooled prevalence estimate.

Collectively, these assessments ensured that key conclusions on the systematic review – related to epidemiology, risk factors, diagnostic criteria, interventions, and adverse outcomes were not disproportionately shaped by studies with weaker methodological profiles.

#### 2.4.2 Title-based studies categorization using Python

To classify the 241 included studies into predefined thematic categories, an automated text processing procedure was developed using Python (version 3.10). Python was selected for its stability, wide adoption, support for reproducible workflows, and efficiency in managing large text datasets. This approach reduced human judgement bias, minimized manual misclassification, allowed rapid re-analysis of keywords or category definitions, and ensured consistent application of classification rules. The use of Python thus provided a reliable and standardized method for assigning studies to thematic categories (**Table 2**), thereby enhancing the overall integrity of the systematic review process. .

**Table 2:** Thematic classification of included studies based on study title keywords analysis (n = 241).

Category	Count
Risk Factors	63 studies - Includes studies titles with terms such as “risk”, “predictors”, “associated factors”, “determinants”, etc.
Diagnostics & Screening	41 studies - Includes studies titles referencing “diagnosis”, “screening”, “OGTT”, “testing”, etc.
Adverse Maternal & Neonatal Outcomes	30 studies - Includes studies titles referencing “maternal outcomes”, “neonatal outcomes”, “pregnancy outcomes”, “complications” etc.
Prevalence / Epidemiology	56 studies - Includes studies titles containing “prevalence”, “incidence”, “epidemiology” etc.
Treatment & Management	18 studies - Includes studies titles containing terms such as “treatment”, “management”, “intervention”, “therapy”, “trial” etc.
Uncategorized (no keyword match)	33 studies - Includes studies titles lacking any of the above thematic keywords.

### 2.5 Integration of approaches

The bibliometric analysis provided a quantitative overview of publication patterns, research clusters, and the evolution of scholarly activity, while the systematic review and meta-analysis offered qualitative, quantitative, and critical appraisal. Integration of both approaches strengthened the inquiry by linking publication trends with methodological quality and thematic findings of individual studies.

Because GDM studies conducted across the African continent showed considerable diversity in diagnostic practices, recruitment strategies, and outcome reporting, a narrative synthesis was adopted. Descriptive information extracted from the studies was compared by region, periods, diagnostic criteria, reported risk factors, and documented outcomes. Statistical procedures described above were directly aligned with the PRISMA flow of study selection, the Quality Assessment table (**Supplementary Table 1**), and thematic Results synthesis. Analyses were conducted on the final sample of 241 studies retained after PRISMA-compliant screening procedures. Quality scores generated through the NOS, JBI, and Cochrane RoB tools informed descriptive summaries, study-design comparisons, and subsequent sensitivity analyses. These methodological procedures ensured that the Results section accurately reflects both the quantitative distribution and the qualitative rigor of the evidence base.

## 3 Results

### 3.1 Bibliometric analysis results

#### 3.1.1 Overview of findings on GDM research in Africa

**Figure 3:**
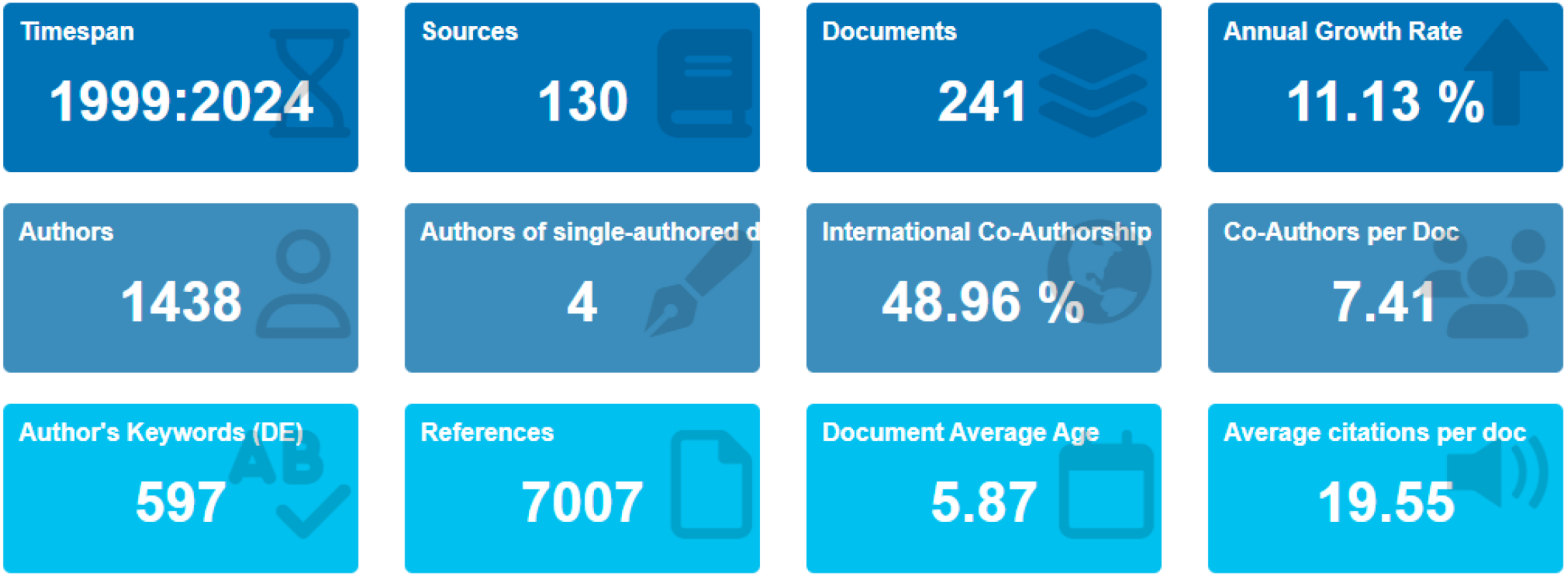
Main bibliometric indicators of African GDM research.

This bibliometric analysis provides a broad overview of the evolution of GDM research in Africa over a twenty-five-year period (**Figure 3****)**. The field has expanded steadily in both volume and thematic scope, with the most rapid growth occurring during the past decade.

The final dataset comprised 241 publications across 130 journals, reflecting the multidisciplinary nature of GDM research, which spans maternal health, endocrinology, public health, and clinical medicine. The relatively recent average publication age (approximately six years) indicates that research on GDM in Africa has accelerated alongside increasing recognition of the condition as a growing contributor to maternal and neonatal morbidity.

Annual productivity trends (**Supplementary Figure 4**) show minimal output between 1999 and the early 2010s, with several years producing only one or two publications. From 2013 onward, however, research output increased substantially, reaching a peak in 2021 and maintaining an average annual growth rate of slightly above 11%. Although a modest decline occurred in 2023, publication activity increased again in 2024, confirming a sustained upward trajectory. This expansion coincides with increasing attention to maternal metabolic disorders and the growing burden of non-communicable diseases in African populations.

**Figure 4:**
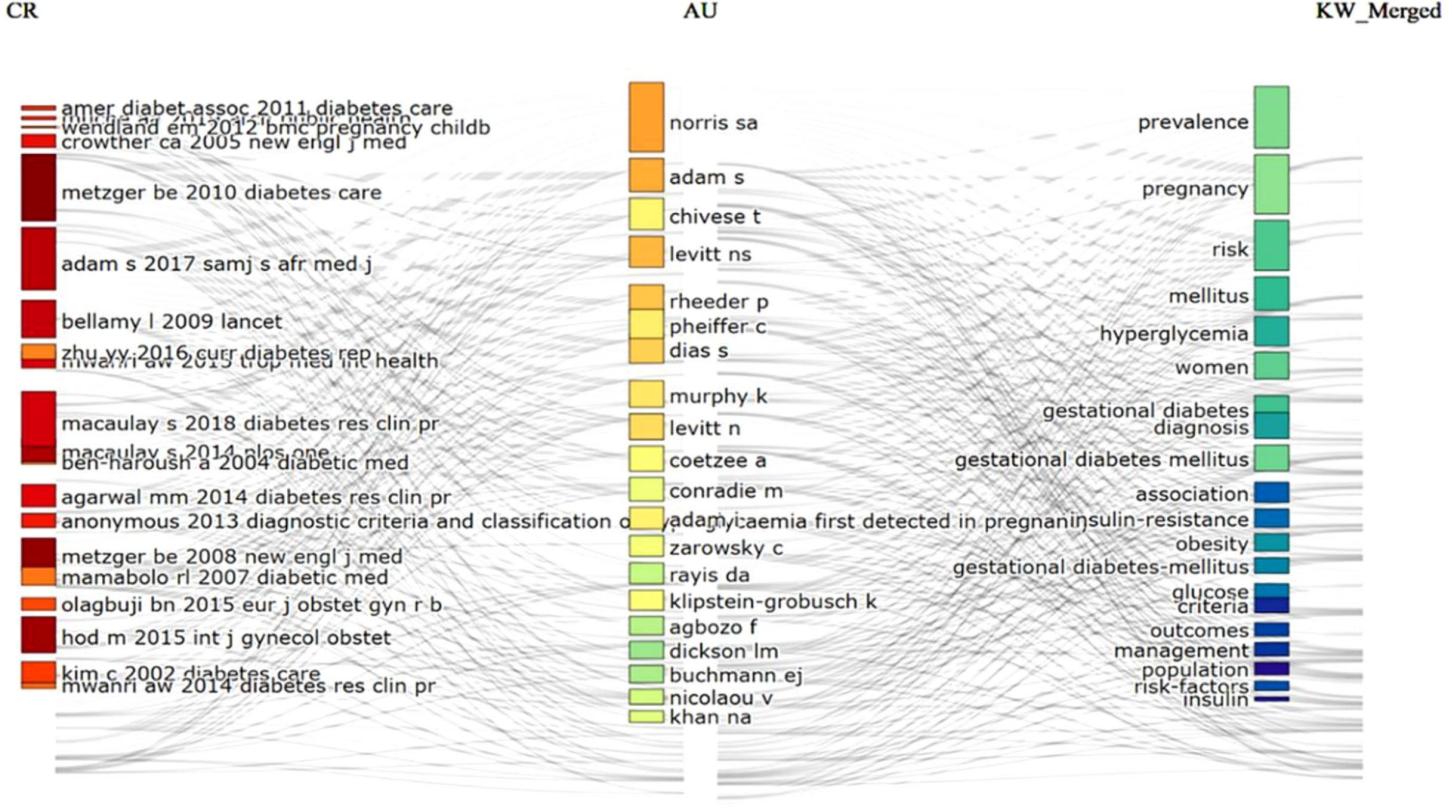
Three-field plot of African GDM research.

Collaboration patterns further highlight the maturation of this research area. A core group of researchers contributed substantially to the literature between 2017 and 2021, helping to shape the field’s development. Keyword analysis shows a broad thematic focus including prevalence, metabolic and demographic risk factors, diagnostic practices, treatment approaches, and maternal or neonatal outcomes. As shown in **Figure 4**, these themes have become increasingly interconnected, indicating a transition from early prevalence reporting toward more integrated investigations of care pathways and clinical consequences. Citation patterns (**Supplementary Figure 5**) support the interpretation of a rapidly developing research field. The dataset references over 7,000 cited sources, with an average of nearly 20 citations per document. Citation peaks observed in the mid-2000s and again in 2022 reflect influential publications that helped shape diagnostic frameworks and research priorities. Lower citation counts in the most recent years likely reflect the normal time lag required for newly published studies to accumulate citations.

Overall, the findings indicate that African GDM research has transitioned from a limited and fragmented body of research into a growing and increasingly cohesive research domain, characterized by expanding collaboration networks, broader thematic coverage, and rising global visibility.

#### 3.1.2 Sources: scientific output on GDM in Africa

The analysis of publication sources illustrates how research dissemination and influence are structured within the field. **Supplementary Figure 6** demonstrates that research output remained very limited before 2010, followed by steady growth after 2013 and a marked acceleration after 2017. This pattern aligns with increasing recognition of gestational diabetes as a major maternal health concern linked to rising obesity, urbanization, and changing dietary patterns.

Among journals, BMC Pregnancy and Childbirth emerges as the most productive outlet, showing consistent growth in publication output. Other important contributors include the International Journal of Gynecology and Obstetrics, PLOS One, and Diabetes Research and Clinical Practice, all of which publish regularly on maternal metabolic health. These journals collectively reflect the intersection of obstetric, metabolic, and public health perspectives in GDM research.

Bradford’s Law analysis (**Supplementary Figure 7**) indicates that a small core group of journals accounts for a large proportion of publications in the field. The same leading journals appear repeatedly within this core zone, suggesting that they play a central role in shaping the scientific and clinical discourse surrounding gestational diabetes in Africa.

The distribution of publications across journals (**Supplementary Figure 8**) confirms this concentration. The BMC Pregnancy and Childbirth journal leads with 18 publications, followed by International Journal of Gynecology and Obstetrics (13 publications), PLOS One (11 publications), and Diabetes Research and Clinical Practice (10 publications). Additional outlets such as Pan African Medical Journal, BMJ Open, and the Journal of Maternal-Fetal and Neonatal Medicine contribute to regional diversity and broader dissemination.

Citation analysis (**Supplementary Figure 9**) reveals that several internationally recognized journals provide the core evidence base for African GDM studies. Diabetes Care receives the highest number of local citations (640), highlighting its influence in establishing diagnostic and clinical frameworks. Other frequently cited journals include Diabetes Research and Clinical Practice, BMC Pregnancy and Childbirth, American Journal of Obstetrics and Gynecology, and Diabetic Medicine.

Source impact analysis using the H-index (**Supplementary Figure 10**) reinforces these findings. BMC Pregnancy and Childbirth demonstrates the strongest influence with a local H-index of 10, followed by the International Journal of Gynecology and Obstetrics (H = 8). Together, these results indicate that a relatively small number of specialized maternal health and diabetes journals serve as the principal platforms for disseminating African research on gestational diabetes.

Overall, the publication landscape reflects a maturing research ecosystem in which core journals anchor the field while a wider range of outlets contributes additional disciplinary and regional perspectives.

#### 3.1.3 Authorship patterns of GDM research in Africa

Authorship patterns illustrate the evolution of scholarly engagement in African GDM research. Early contributions were sporadic, with limited numbers of authors publishing prior to 2015. A marked increase in both productivity and collaboration occurs after 2017, reflecting growing research capacity and rising recognition of gestational diabetes as a significant public health challenge. As shown in **Supplementary Figure 11**, author productivity increased substantially between 2018 and 2021, with several highly cited studies published during this period. These publications frequently addressed clinically relevant topics such as risk factors, diagnostic criteria, and pregnancy outcomes, contributing to their high citation impact.

Contribution patterns are uneven (**Supplementary Figure 12**), with one author producing 20 publications, while several others contributed between 6 and 9 papers. This concentration of productivity is typical in developing research areas where a small group of leading scholars drives early progress and establishes research priorities. Author productivity distribution (**Supplementary Figure 13**) follows Lotka’s Law, with most researchers contributing only one or two publications while a smaller group publishes extensively. This structure indicates a stable but expanding field that continues to attract new contributors.

Notably, the citation influence also varies. **Supplementary Figure 14** shows that Norris SA has the highest H-index (H=10) within the dataset, followed by Adam S (H = 7). Citation counts (**Supplementary Figure 15**) further highlight Levitt NS and Norris SA as the most cited contributors, with 26 and 23 citations respectively. Additional authors, including Alberts M, Delemarre-van-de-Waal HA, Mamabolo RL, and Steyn NP, contribute to the broader scholarly network.

Collectively, these findings indicate that African GDM research is shaped by a core group of influential scholars, supported by a wider base of occasional contributors who are expanding the field’s disciplinary and geographic reach.

##### 3.1.3.1 Authors’ affiliation

Institutional contributions to African GDM research reveal a similar pattern of concentration and expansion. **Supplementary Figure 16** shows limited institutional participation before 2010, followed by gradual growth and a marked increase after 2018, reflecting both expanded research capacity and growing interest in maternal metabolic health.

The University of the Witwatersrand emerges as the leading institutional contributor, with publication output rising sharply after 2017 to reach 82 publications. Other institutions such as the University of Cape Town and Stellenbosch University also show steady contributions, while Cairo University, the South African Medical Research Council, and the University of Pretoria contribute at smaller but consistent levels.

As illustrated in **Supplementary Figure 17**, research activity remains concentrated within a limited number of highly productive institutions. However, the presence of international partners (including the University of Oxford and the University of Montreal) demonstrates the importance of cross-border collaboration in strengthening methodological capacity and expanding research networks.

Overall, the institutional landscape reflects growing but uneven research capacity, with a small number of universities acting as regional hubs for gestational diabetes research.

##### 3.1.3.2 Authors’ countries

The geographic distribution of authorship reveals notable disparities in research production across the continent. **Supplementary Figure 18** shows that South Africa dominates African GDM research, while Nigeria, Ethiopia, Egypt, Ghana, and Tanzania contribute moderate levels of output. In contrast, large parts of Central Africa show limited or no sustained publication activity. This apparent gap may partly be influenced by the language filter applied in this review, which included only studies published in English. As English is the primary language of proficiency of the research team, restricting the search to English-language publications ensured accurate interpretation, extraction, and synthesis of study findings. However, this may have excluded relevant studies published in other languages, particularly from Francophone or Lusophone African countries, potentially contributing to the lower representation of research from some Central African regions.

Temporal trends (**Supplementary Figure 19**) demonstrate steady growth in research output after 2015, with particularly strong expansion after 2018. South Africa remains the leading contributor, followed by Nigeria, Egypt, and Ethiopia. Contributions from countries such as the United Kingdom highlight the continued importance of international collaboration.

**Figure 5:**
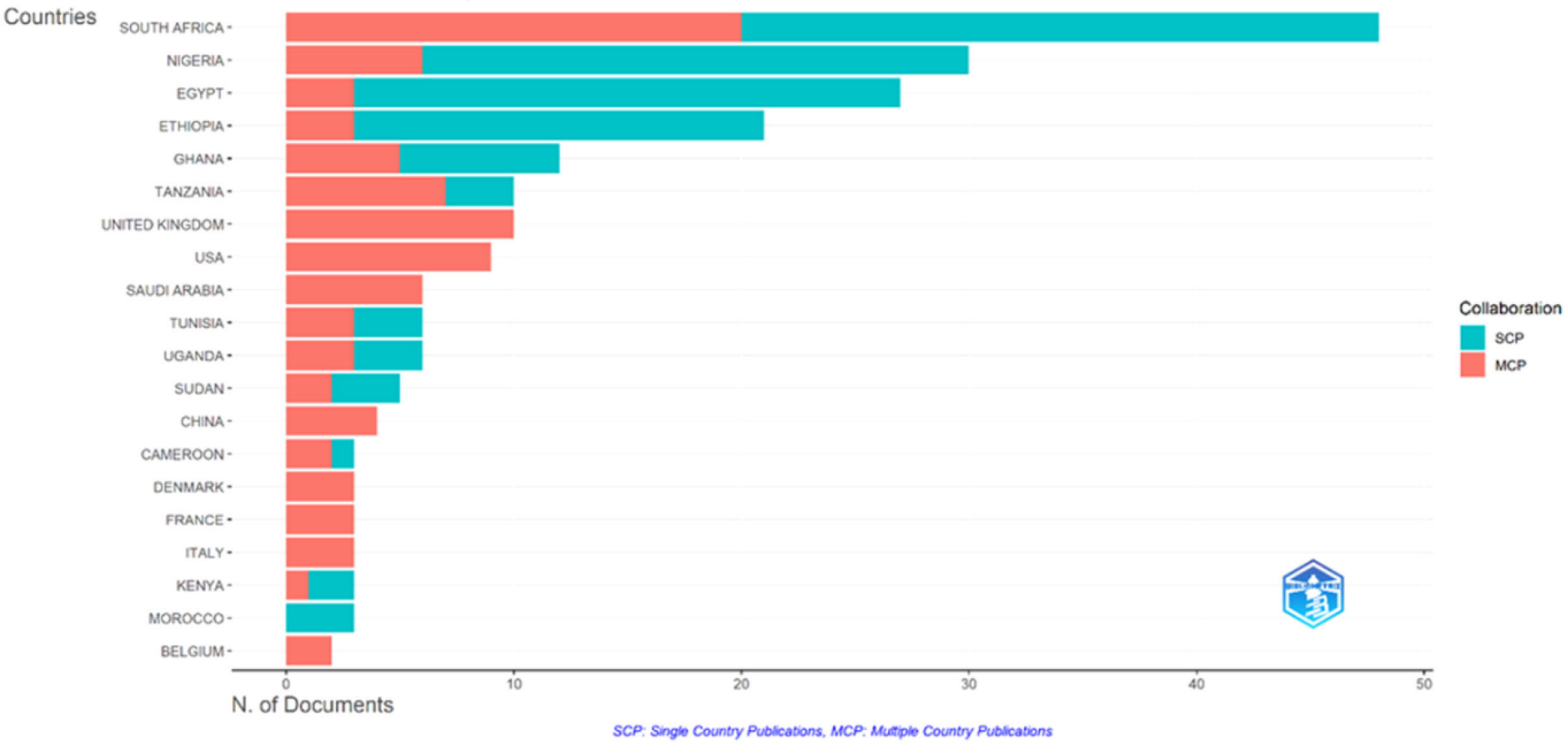
Corresponding author countries: SCP versus MCP in African GDM research.

**Figure 5** further illustrates these patterns by comparing single-country publications (SCP) and multi-country publications (MCP). South Africa leads in both categories, indicating strong domestic research capacity as well as extensive international collaboration. Nigeria and Egypt show similar patterns, although Nigeria appears more engaged in international partnerships.

Citation analysis by country (**Supplementary Figure 20**) reinforces South Africa’s leadership, with 891 citations, followed by China (738) and France (451). Nigeria also demonstrates strong impact with 438 citations, while other African countries show smaller citation counts.

Overall, these findings indicate that African GDM research is rapidly expanding but unevenly distributed, with a few countries driving most scientific output and influence.

#### 3.1.4 Documents: GDM research publication patterns in Africa

Document-level analysis reveals a research landscape shaped by a combination of globally influential studies and regionally focused investigations. Global citation patterns (**Supplementary Figure 21**) show that a small number of landmark publications dominate the field, with one study receiving 677 citations and several others receiving between 60 and 321 citations. These highly cited works often establish widely used diagnostic criteria and epidemiological frameworks.

Within the African literature itself, however, citation patterns appear more balanced. **Supplementary Figure 22** shows local citations distributed primarily between 6 and 15, indicating reliance on a shared body of regionally relevant research addressing diagnostic practices, maternal care, and context-specific risk factors.

Reference analysis (**Supplementary Figure 23**) highlights the continued influence of key methodological studies, including those that established international diagnostic standards for gestational diabetes. These references form the clinical and epidemiological foundation upon which much African research is built.

The document coupling map in **Supplementary Figure 24** illustrates the conceptual structure of the literature. Major thematic clusters focus on insulin resistance, obesity, antenatal care, pregnancy outcomes, and metabolic health, reflecting the strong emphasis on maternal metabolic risk. Secondary clusters link metabolic mechanisms to fetal outcomes and pregnancy complications, while peripheral clusters explore emerging topics such as vitamin D, metformin use, and environmental exposures.

Reference Publication Year Spectroscopy (**Supplementary Figure 25**) demonstrates a steady rise in citation activity beginning in the late 1990s, with major peaks corresponding to landmark publications that shaped diagnostic criteria and treatment approaches. The recent decline in citation frequency reflects the typical lag before newly published studies accumulate citations.

Together, these results indicate that African GDM research is increasingly grounded in shared diagnostic frameworks and epidemiological methods, while gradually expanding into new areas of investigation relevant to regional clinical contexts.

#### 3.1.5 Conceptual framing: GDM research focus areas in Africa

Keyword analysis reveals the evolving conceptual focus of GDM research in Africa. As shown in **Supplementary Figure 26**, research activity remained limited before 2015 but expanded rapidly thereafter, with increased attention to terms such as pregnancy, prevalence, obesity, hyperglycemia, and risk factors.

Frequency analysis (**Supplementary Figure 27**) identifies pregnancy as the most common keyword, followed by women, prevalence, obesity, hyperglycemia, and gestational diabetes mellitus. These patterns highlight the strong emphasis on maternal vulnerability, metabolic risk, and disease burden.

Trend analysis (**Supplementary Figure 28**) demonstrates a shift in research priorities over time. Between 2009 and 2014, studies focused largely on general metabolic health and glycemic control. Between 2015 and 2019, research expanded toward clinical mechanisms such as insulin resistance, glucose tolerance, and inflammation. Since 2020, attention has increasingly focused on prevalence, management, pregnancy outcomes, and regional epidemiology.

Keyword clustering (**Supplementary Figures 29, 34**) confirms that African GDM research is strongly centered on epidemiology and maternal health, with frequent terms including pregnancy, prevalence, gestational diabetes mellitus, and women. Diagnostic issues – such as glucose testing and diagnostic criteria – remain important, while risk factors including obesity and insulin resistance are increasingly investigated.

The word cloud analysis (**Supplementary Figure 34**) reinforces these findings, highlighting a dominant focus on epidemiology, diagnosis, and metabolic dysfunction, with maternal outcomes and geographic context appearing as secondary themes.

Together, these results suggest that African GDM research has evolved from early descriptive studies toward more integrated analyses of metabolic mechanisms, clinical management, and maternal outcomes, although research on long-term consequences and interventions remains comparatively limited.

#### 3.1.6 Conceptual structures of GDM research in Africa

**Figure 6:**
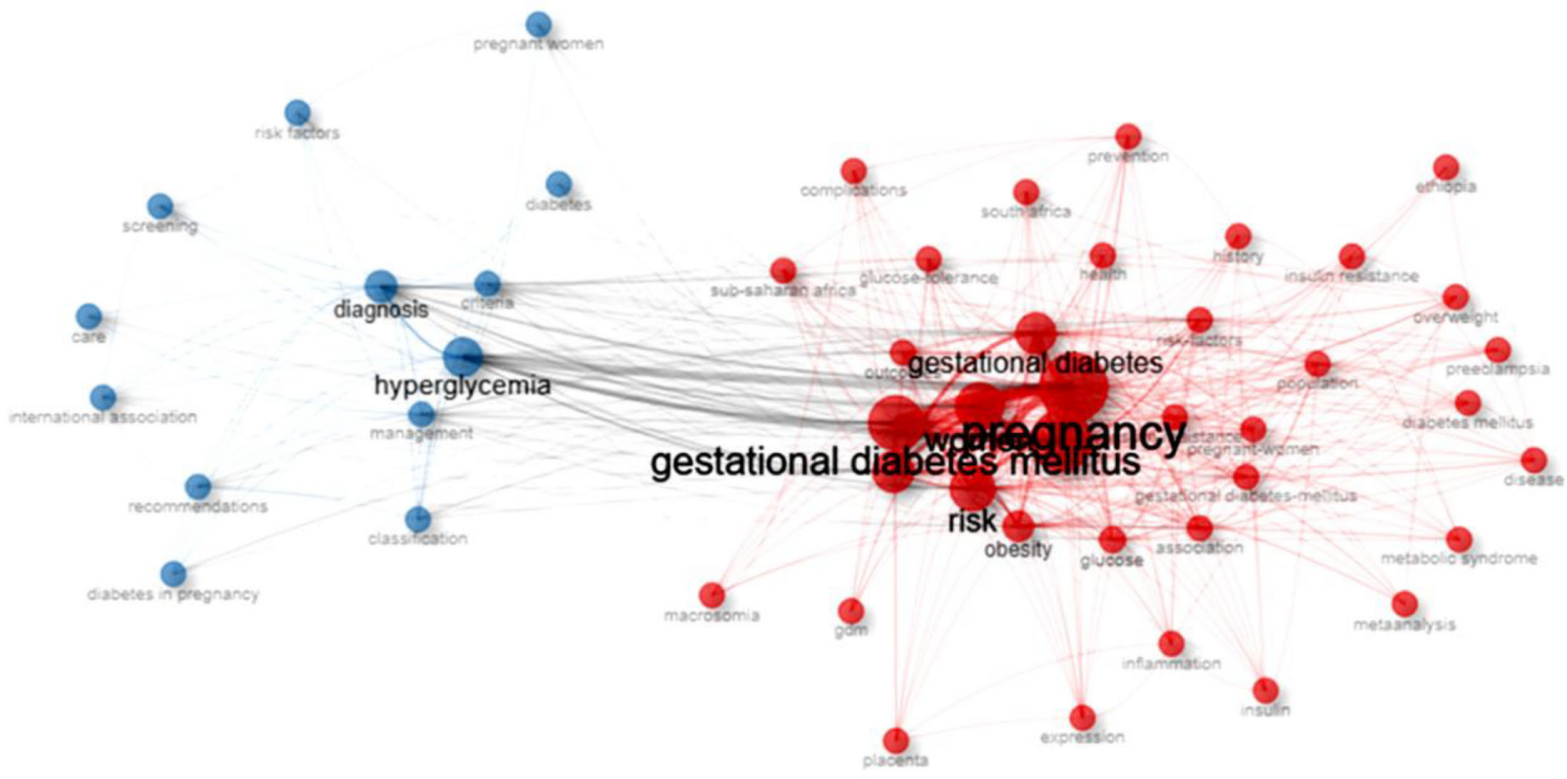
Co-occurrence network in African GDM research.

The conceptual structure of African GDM research reflects increasing integration between epidemiological, metabolic, and clinical perspectives. In **Figure 6**, the keyword co-occurrence network reveals two dominant clusters: a large cluster centered on pregnancy, obesity, insulin resistance, and maternal-neonatal complications, and a second cluster focused on screening, diagnosis, and clinical management. The strong connections between these clusters indicate that diagnostic practices are closely linked to research on disease prevalence and outcomes.

**Figure 7:**
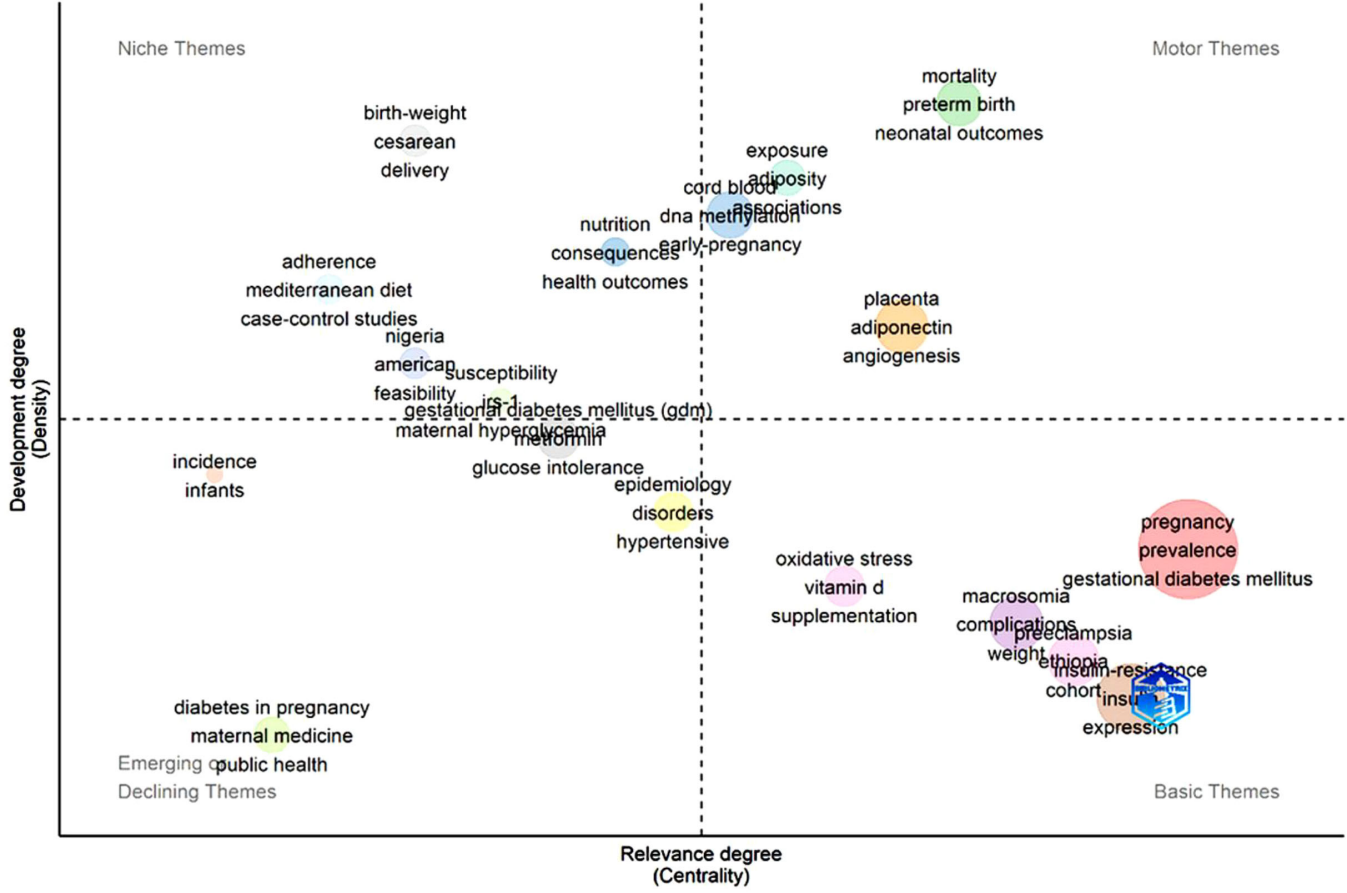
Thematic mapping of African GDM research.

The thematic map in **Figure 7** further categorizes the literature into four conceptual areas. **Basic themes** include pregnancy, GDM, prevalence, insulin resistance, and preeclampsia. **Motor themes**, such as neonatal outcomes and mortality, represent well-developed areas of investigation. **Niche themes**, including birth weight and caesarean delivery, represent specialized topics, while **emerging themes** include public health and broader maternal health perspectives.

**Figure 8:**
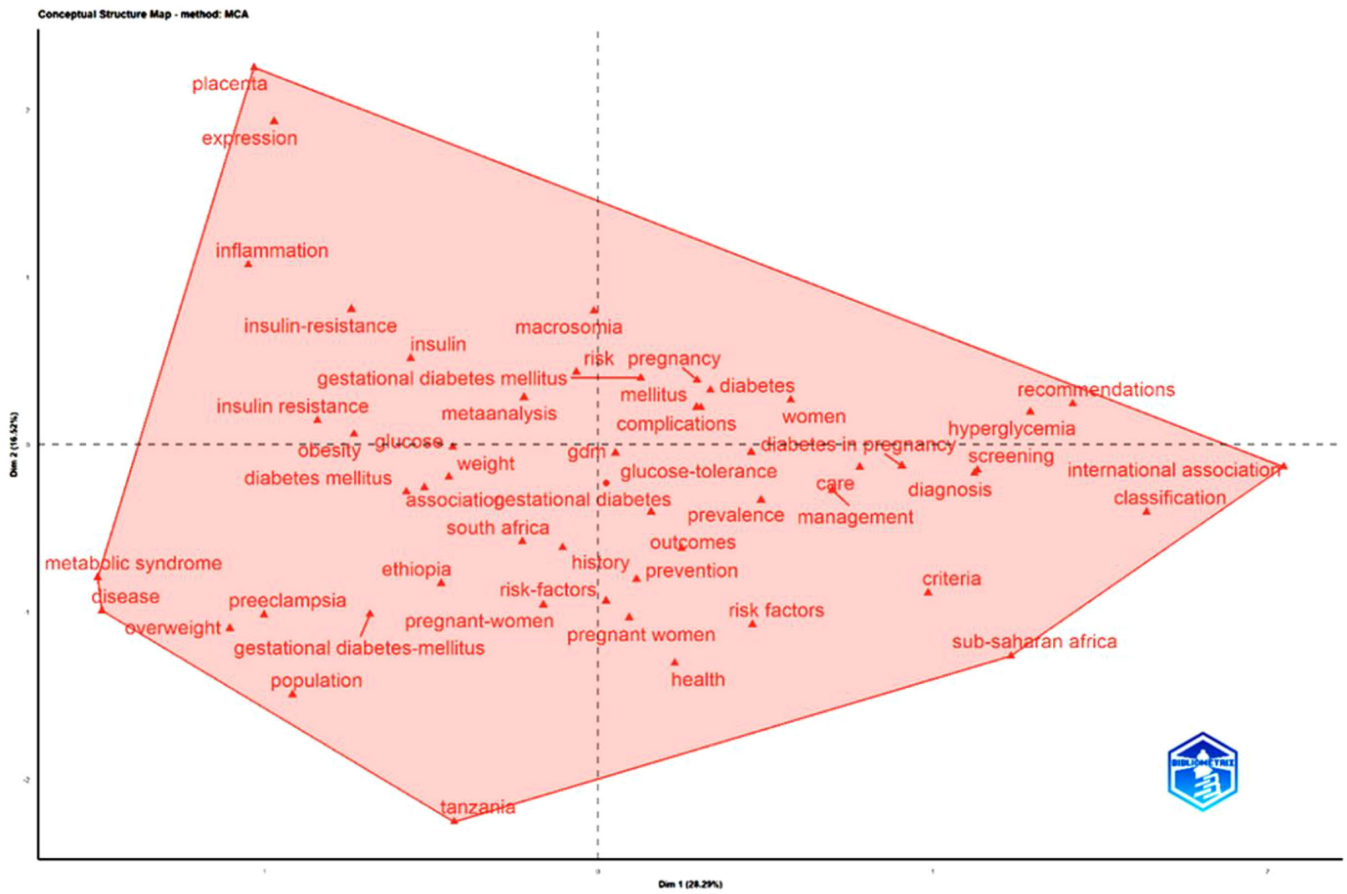
Conceptual structure map (factorial mapping of GDM research in Africa).

Hierarchical clustering (**Supplementary Figure 30**) reinforces these groupings by identifying distinct clusters of metabolic mechanisms, maternal complications, diagnostic frameworks, and regional epidemiology. The conceptual structure map generated through Multiple Correspondence Analysis (**Figure 8**) shows that the intellectual core of the field revolves around GDM, pregnancy, prevalence, risk factors, and maternal outcomes. Mechanistic topics such as inflammation and metabolic syndrome appear on the periphery, indicating emerging but still limited research activity.

Overall, the conceptual structure reveals a field organized around three major streams: metabolic mechanisms, clinical epidemiology, and diagnostic standards. While research on prevalence and pregnancy outcomes is well developed, public health interventions and long-term consequences remain relatively underexplored.

#### 3.1.7 Intellectual structures of GDM research in Africa

The citation network analysis (**Supplementary Figures 31, 32**) reveals that African GDM research has evolved into a more interconnected intellectual structure over time. Several influential studies – particularly Metzger BE (2010), Hod M (2015), and Mwanri AW (2015) – serve as central references guiding research on diagnostic criteria, epidemiology, and clinical management.

Temporal citation mapping (**Supplementary Figure 32**) indicates that earlier studies provided the methodological foundation for later investigations. Publications from Mamabolo RL (2007) through Macaulay S (2018) form a coherent citation lineage linking early epidemiological work with more recent clinical studies. Smaller clusters of emerging research topics are also visible, indicating areas that are still developing within the broader field.

Overall, these analyses demonstrate that African GDM research is grounded in global diagnostic frameworks while increasingly informed by regionally generated epidemiological evidence.

#### 3.1.8 Social structures of GDM research in Africa

Collaboration networks illustrate the social organization of the field. **Supplementary Figure 33** highlights that research activity is concentrated around several influential scholars, with Norris SA forming a central collaboration hub connecting multiple research teams. At the same time, several smaller clusters represent emerging or locally focused research groups.

**Figure 9:**
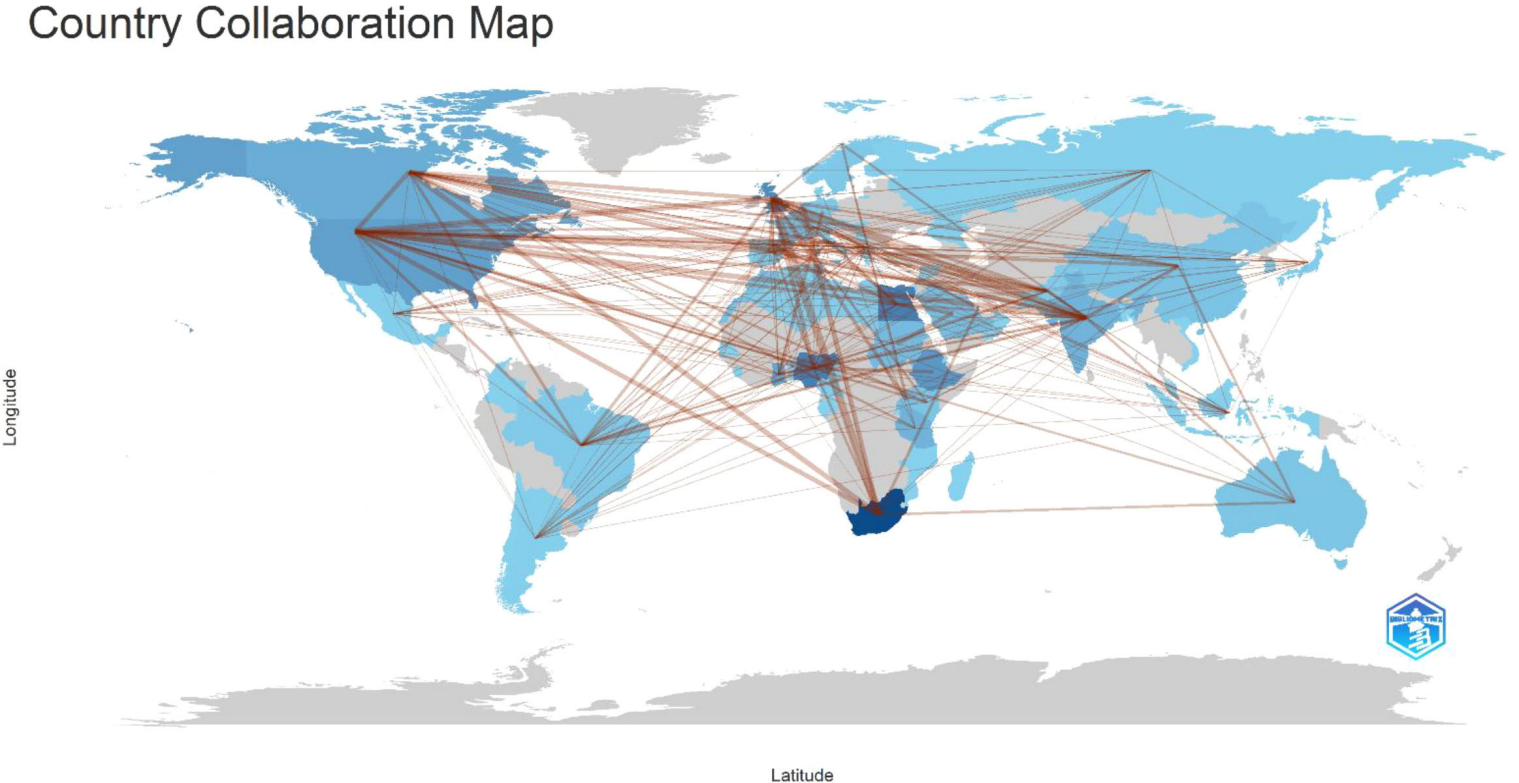
Countries collaboration networks of GDM research in Africa.

**Figure 9** adds a geographic perspective, illustrating international research partnerships. South Africa appears as the primary hub, collaborating extensively with both African and international institutions. Countries such as Nigeria, Kenya, Ethiopia, and Ghana also participate in international collaborations, though their networks are less extensive.

Many collaborations involve institutions in Europe, North America, Asia, and Oceania, highlighting the importance of global partnerships in supporting research capacity and funding. These findings suggest that while African GDM research is expanding, collaboration remains concentrated within a limited number of institutions and networks.

Although bibliometric findings primarily describe the structure and development of the research field, they provide important insights into the clinical and policy context of gestational diabetes research in Africa. The rapid growth in publications since the mid-2010s reflects increasing recognition of GDM as a significant contributor to maternal and neonatal morbidity in African populations. The strong concentration of research activity in a limited number of countries and institutions, particularly in Southern and parts of West Africa, highlights persistent geographic gaps in evidence generation that may influence the representativeness of epidemiological estimates across the continent. The thematic emphasis on prevalence, metabolic risk factors, and diagnostic criteria suggests that much of the existing literature has focused on understanding disease burden and improving detection. However, comparatively less attention has been directed toward long-term maternal outcomes, postpartum diabetes prevention, or health system interventions. These patterns underscore the importance of expanding both the geographical coverage and clinical scope of future research. Against this backdrop, the systematic review and meta-analysis presented in the following sections synthesize the available empirical evidence to quantify the burden of gestational diabetes and identify key clinical determinants relevant to maternal and neonatal health policy in African settings.

### 3.2 Systematic review and meta-analysis results

#### 3.2.1 Quality assessment results

Inter-rater reliability analyses demonstrated strong to excellent agreement between the two independent reviewers, with a weighted Cohen’s kappa of 0.82 (95% CI: 0.76–0.89) and an intraclass correlation coefficient of 0.88 (95% CI: 0.84–0.93), indicating consistent quality scoring across studies. Between-group comparisons using the Kruskal-Wallis test showed significant differences in methodological quality across study designs (H = 18.42, p = 0.00035) with a moderate effect size (η²_H = 0.091), and *post-hoc* Mann-Whitney U tests revealed that cohort and cross-sectional studies generally scored higher than case-control and interventional studies.

Associations with study characteristics indicated that newer studies (ρ = 0.19, p = 0.003) and those with larger sample sizes (ρ = 0.24, p = 0.0006) had slightly higher quality scores, while studies using IADPSG diagnostic criteria outperformed those using WHO thresholds (r = 0.27, p = 0.002). Sensitivity analyses confirmed that excluding low-quality studies had minimal impact on key outcomes, including median GDM prevalence and pooled odds ratios for obesity and advanced maternal age as risk factors, supporting the robustness of the findings.

Correlation analysis demonstrated a statistically significant positive relationship between methodological quality and publication year. Using Spearman’s rank correlation, the effect size was ρ = 0.307, indicating a modest but meaningful association, with higher-quality studies tending to appear in more recent years. The bootstrapped 95% confidence interval ranged from 0.071 to 0.498, and the association was statistically significant (*p* = 0.0169).

The magnitude and precision of this effect-size estimate support the conclusion that improvements in study quality have occurred over time within the included literature. All effect sizes and confidence intervals for additional comparisons (e.g., Kruskal-Wallis, Mann-Whitney U, ICC, and κ) are reported in the **Supplementary Materials**.

#### 3.2.2 Study designs

Among the 241 studies reviewed, four key methodological approaches were identified: cross-sectional studies (93; 38.6%), cohort studies (45; 18.7%), case-control studies (31; 12.9%), and intervention, clinical trial, or quasi-experimental studies (32; 13.3%). Studies sample sizes varied widely, ranging from 21 to 19,810 participants, reflecting the diverse antenatal care settings across Africa.

#### 3.2.3 Geographic distribution of studies

The studies encompassed all five African regions, with country information derived from titles, abstracts, and journal metadata. Overall, at least 33 countries were represented, demonstrating broad geographic coverage across the continent.

#### 3.2.4 Topic distribution of included studies

The thematic analysis of 241 studies identified five primary research domains using a structured keyword mapping of study titles: risk factors, diagnostic and screening methods, adverse maternal and neonatal outcomes, prevalence and epidemiology, and treatment or management strategies for GDM.

The largest portion of studies focused on risk factors (63; 26.1%), followed by prevalence and/or epidemiology (56; 23.2%). Research on diagnostic and screening approaches accounted for 41 studies (17.0%), while adverse maternal and neonatal outcomes accounted for 30 studies (12.4%) and treatment or management strategies (18; 7.5%) represented smaller proportions. Notably, 33 studies (13.7%) could not be classified within these GDM-specific domains based on title information alone. These uncategorized studies largely covered general maternal health, obstetric care, metabolic or endocrine aspects related to GDM, pregnant diabetes populations, laboratory-based research, or contained limited descriptive details.

Overall, the distribution indicates a strong emphasis on risk factors and diagnostic methods, with comparatively fewer studies addressing management strategies or maternal and neonatal outcomes, highlighting areas for future research.

##### 3.2.4.1 Prevalence of gestational diabetes mellitus in Africa

To estimate the prevalence of GDM in Africa from the dataset, a sub-set of studies in the dataset with reported extractable numerators and denominators for GDM were included in a quantitative synthesis of prevalence estimates. These studies include research from different regions of the continent and applied a range of diagnostic criteria, resulting in considerable variability in observed prevalence. Forty-four studies from 17 African countries conducted between 1999 and 2024 met the inclusion criteria. Sample sizes ranged from 101 to 9,314 participants, with prevalence estimates spanning 0.7% to 45.9%. Considerable variability in diagnostic approaches, screening strategies, and population characteristics necessitated stratification into three temporal periods: 1999–2024, 2014–2024, and 2020–2024.

Sensitivity analyses were also conducted to assess the robustness of the pooled prevalence estimate for the 1999–2024 dataset, employing leave-one-out (LOO) procedures, Baujat-style evaluations, and influence diagnostics derived from changes in the Freeman-Tukey-transformed estimates. The LOO approach showed that the pooled GDM prevalence remained remarkably stable, with the removal of individual studies altering the estimate by no more than roughly 1.5% points compared with the full-model value of 12.62%. Even the studies exerting slightly greater influence – such as those by Minsart et al. (2014), Darling et al. (2014), Grunnet et al. (2020), and Karamanos et al. (2014) – did not shift the estimate outside the original confidence interval or meaningfully alter its direction (57–60). These results indicate that no single dataset disproportionately affected the final prevalence.

The Baujat-style assessment produced a similar pattern: studies contributing more notably to heterogeneity also displayed marginally higher influence on the pooled estimate, yet none reached levels suggestive of distortion. The influence appeared to be distributed across multiple studies, consistent with the diversity of diagnostic practices, population characteristics, and screening approaches documented across African research settings.

Additional influence diagnostics, including changes in the transformed prevalence and weighted influence statistics, did not identify any extreme outliers. Even when the most influential studies were excluded, heterogeneity remained high (I² ≈ 98%), confirming that variability originates from broad epidemiological and methodological differences rather than from isolated influential sources. Collectively, these findings support the stability of the meta-analytic results and affirm that the observed heterogeneity reflects genuine underlying differences rather than artefacts of individual studies.

Across all diagnostics, the pooled prevalence estimates for 1999–2024 was highly robust.

- No study materially altered the pooled prevalence.
- The pattern of heterogeneity was consistent and widely distributed.
- Influence diagnostics consistently identified the same small group of moderately influential studies, none of which threatened the validity of the meta-analytic conclusions.

Together, these results confirm that the final pooled estimates – and the corresponding confidence and prediction intervals – are stable and reliable, even under systematic perturbation of the dataset.

###### 3.2.4.1.1 Overall prevalence of gestational diabetes mellitus (1999–2024)

**Figure 10:**
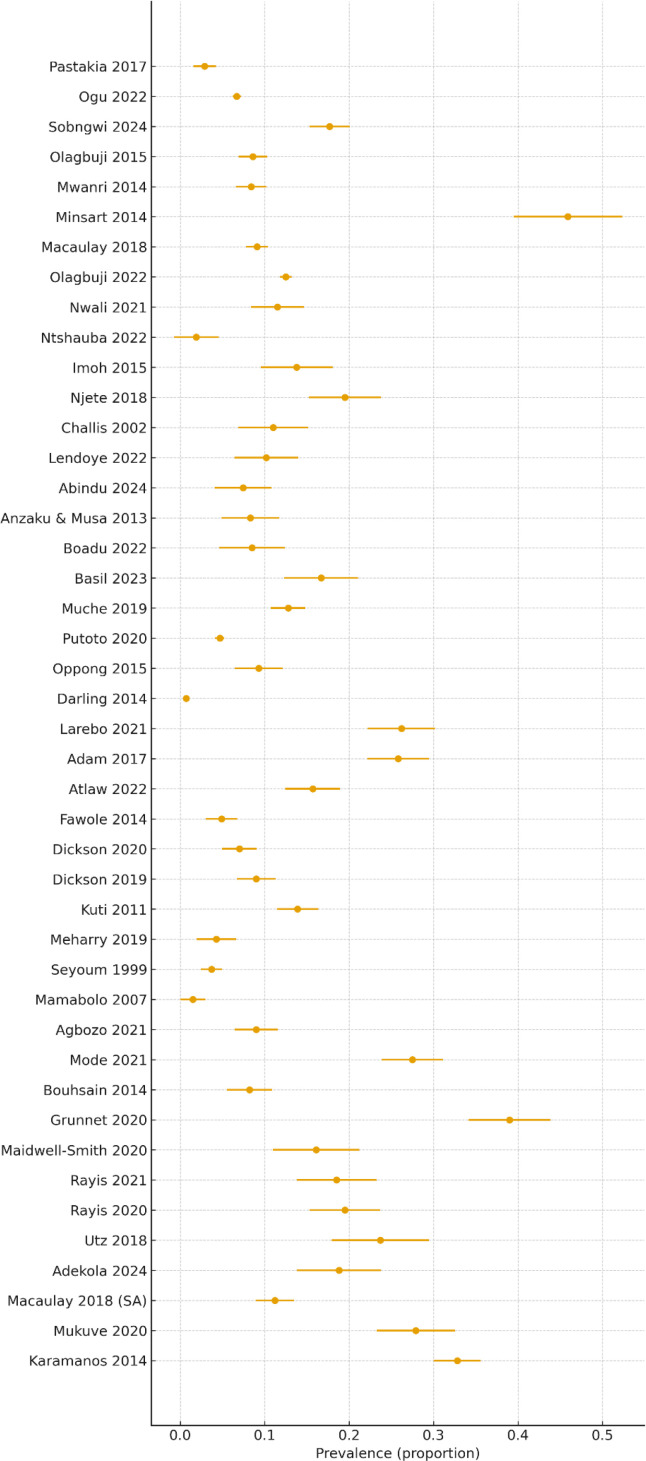
Forest plot of GDM prevalence (1999–2024).

Using a Freeman-Tukey-transformed and DerSimonian-Laird random-effects models, the pooled GDM prevalence across 44 studies was 12.62% (95% CI: 10.23%–15.21%). Heterogeneity was substantial (I² = 98.4%), and the 95% prediction interval (1.18%–33.50%) indicated wide variability in expected estimates across African settings. The lower fixed-effect estimate (9.26%) further supported the presence of pronounced between-study differences. This overall estimate and the considerable between-study variability are visually illustrated in **Figure 10**, which presents the forest plot of GDM prevalence estimates across studies conducted between 1999 and 2024.

###### 3.2.4.1.2 Prevalence of GDM (2014–2024)

**Figure 11:**
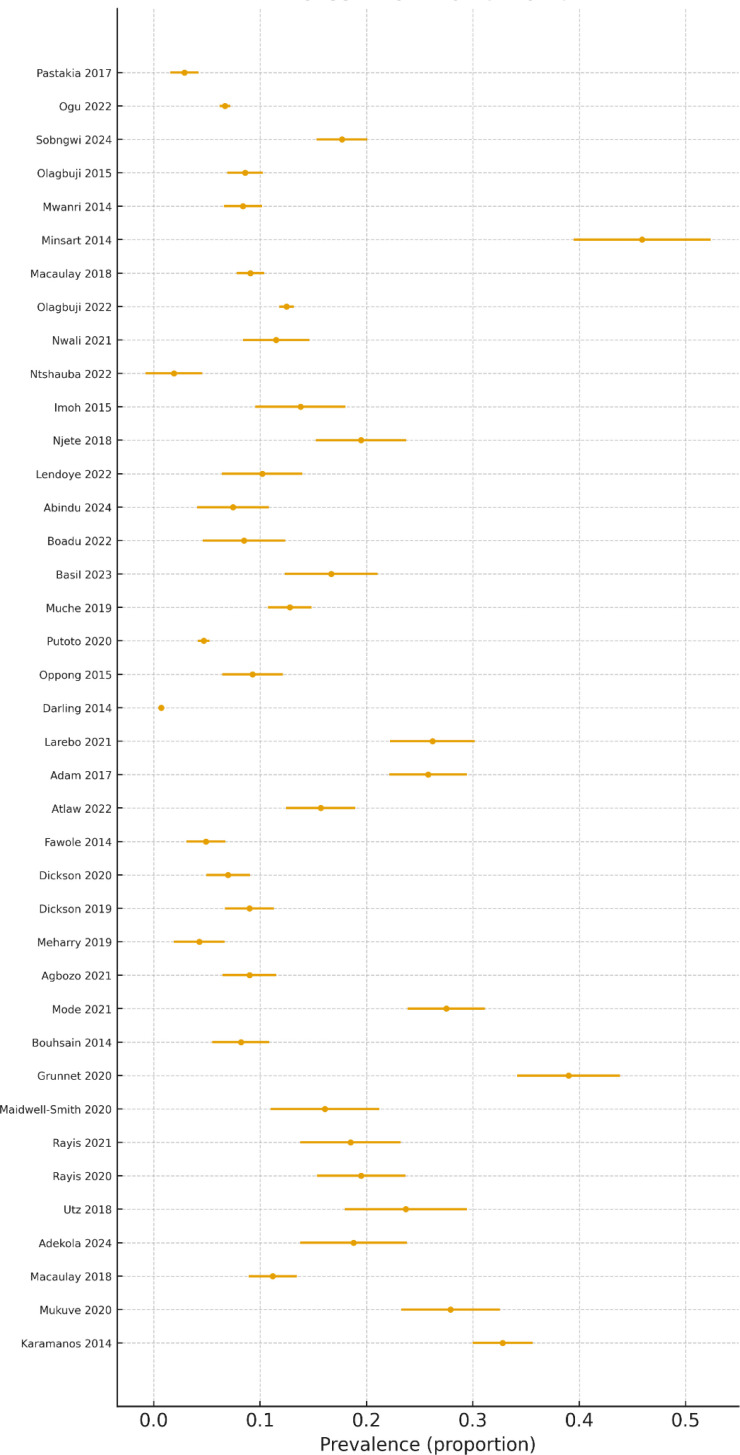
Forest plot of GDM prevalence (2014–2024).

Thirty-nine studies contributed to the pooled estimate for this period. The random-effects prevalence was 13.43% (95% CI: 10.80%–16.30%), with very high heterogeneity (I² = 98.55%). The prediction interval (1.34%–35.11%) again reflected broad dispersion. Compared with 1999–2024, the prevalence showed a modest increase. This modest increase in prevalence and the substantial heterogeneity across studies are clearly depicted in **Figure 11**, which illustrates the forest plot of GDM prevalence estimates for the 2014–2024 period.

###### 3.2.4.1.3 Recent prevalence (2020–2024)

**Figure 12:**
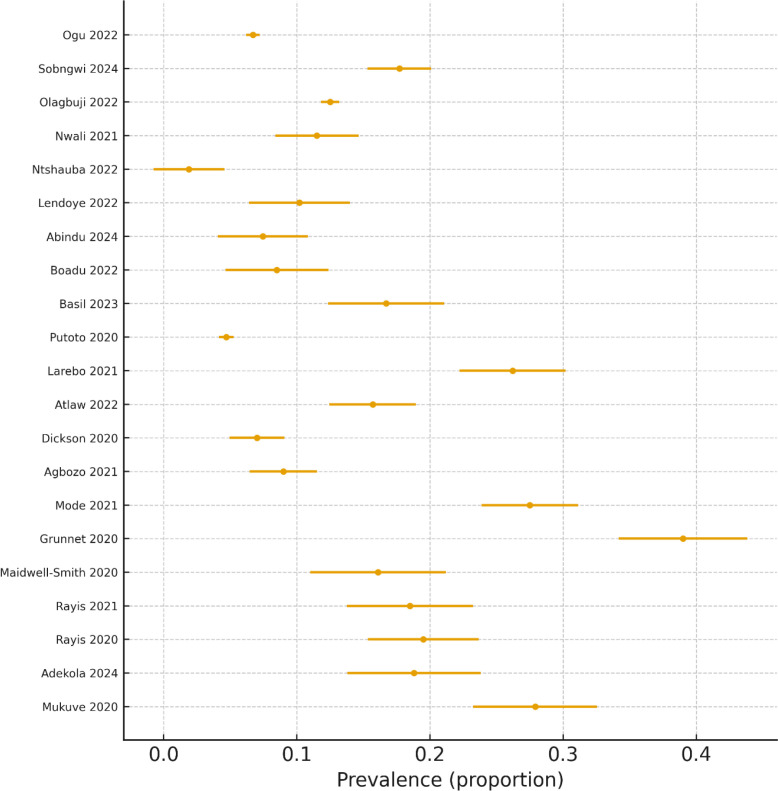
Forest plot of GDM prevalence (2020–2024).

Twenty-one studies published between 2020 and 2024 were included. The pooled prevalence rose further to 14.52% (95% CI: 11.42%–17.92%), with persistently high heterogeneity (I² = 98.16%). The prediction interval (2.74%–33.33%) confirmed ongoing variability despite more contemporary diagnostic practices. The upward trend in prevalence alongside the persistent heterogeneity is further highlighted in **Figure 12**, which displays the forest plot of GDM prevalence estimates for studies conducted between 2020 and 2024.

In summary, **Table 3** indicated that substantial heterogeneity persisted across all analytic periods (I² > 98%), underscoring significant methodological and population differences. Contributing factors included variation in diagnostic thresholds (IADPSG, WHO 1999, Carpenter-Coustan, NICE), gestational age at screening, rural-urban differences, and socioeconomic and health-system contexts. These findings consistently supported the use of random-effects modeling.

**Table 3:** Summary of random-effects pooled prevalence estimates and prediction intervals (Freeman–Tukey + DerSimonian-Laird Method).

Period	Pooled prevalence (%)	95% CI	Prediction interval	Heterogeneity
1999–2024	12.62	10.23-15.21	1.18-33.50	I² = 98.40%
2014–2024	13.43	10.80-16.30	1.34-35.11	I² = 98.55%
2020–2024	14.52	11.42-17.92	2.74-33.33	I² = 98.16%

The findings further demonstrate a high and progressively increasing GDM burden in Africa, with estimates rising from 12.62% (1999–2024) to 14.52% (2020–2024). Although prediction intervals remained wide, reflecting heterogeneous study conditions, the upward trajectory aligns with rising obesity rates, urbanization, lifestyle changes, and evolving diagnostic criteria. This growing burden highlights the need for standardized screening policies, improved antenatal glucose testing, targeted prevention strategies, and context-specific public health planning.

The meta-analysis demonstrated extremely high between-study heterogeneity across all analytical periods, with I² statistics exceeding 98% and correspondingly wide prediction intervals. These findings indicate substantial variability in reported GDM prevalence across African studies, reflecting differences in study populations, healthcare settings, diagnostic criteria, and screening strategies. Under such conditions, pooled prevalence estimates should be interpreted cautiously and primarily as summary indicators of the distribution of reported values rather than precise estimates applicable to specific African populations. Across the included studies, reported prevalence ranged widely from less than 1% to over 40%, highlighting the considerable contextual and methodological variability across settings. Random-effects meta-analysis using the DerSimonian-Laird estimator with Freeman-Tukey transformation produced pooled prevalence estimates of 12.62% for the overall study period, 13.43% for the intermediate analytical period, and 14.52% for the most recent period, although the wide prediction intervals indicate that true prevalence in individual African settings may vary substantially beyond these averages. This high heterogeneity is largely attributable to differences in diagnostic criteria, particularly the increasing adoption of IADPSG/WHO 2013 thresholds, as well as variations in screening strategies such as universal OGTT versus selective or risk-based testing.

In addition, the methodological variability observed across studies also provides important context for interpreting the substantial heterogeneity detected in the meta-analysis. Differences in study design, sample size, diagnostic criteria, screening strategies, and confounder adjustment contributed to variability in reported prevalence estimates and epidemiological findings across settings. In particular, the predominance of cross-sectional and hospital-based studies with varying diagnostic protocols likely amplified the observed between-study heterogeneity. Consequently, while the included studies were generally of moderate methodological quality, these structural and methodological differences underscore the need to interpret pooled estimates cautiously and highlight the importance of standardized diagnostic practices and study designs in future GDM research across Africa.

##### 3.2.4.2 Diagnostic criteria and screening methods

African studies on GDM demonstrated considerable heterogeneity in both diagnostic criteria and screening approaches.

###### 3.2.4.2.1 Diagnostic criteria

- IADPSG/WHO 2013 thresholds were the most frequently applied, used in approximately 40% of studies, particularly in East and Southern Africa (13, 61–63).
- WHO 1999 and 2013 criteria remained common in West African research (64, 65), especially in earlier studies (6, 66, 67).
- ADA and NICE guidelines were less frequently adopted, mainly in North African contexts (60).
- A minority of studies applied other or unspecified criteria.

###### 3.2.4.2.2 Screening methods

The 75g oral glucose tolerance test (OGTT) was the predominant diagnostic tool across the continent (13, 65, 68–73). Fasting plasma glucose was often used in resource-limited settings (59, 74, 75), while HbA1c was rarely applied, limited to select multi-country cohorts (76, 77). A two-step approach involving a glucose challenge test followed by OGTT was reported in a few North African studies.

###### 3.2.4.2.3 Impact of screening approach

Universal OGTT (75g) screening generally identified higher rates of GDM compared with selective or risk-based testing, which missed a substantial proportion of cases, particularly among younger or non-obese women (78–80).

###### 3.2.4.2.4 Challenges to standardized diagnosis

Key barriers included lack of harmonization across countries, inconsistent gestational age at screening, limited availability of universal screening, cost constraints, laboratory capacity limitations, and variation in glucose measurement techniques (69, 81). These factors complicate comparisons of GDM prevalence and impede accurate estimation across Africa.

##### 3.2.4.3 Risk factors for GDM in Africa

Across the included studies, several maternal, obstetric, and lifestyle factors were consistently associated with GDM in African populations.

###### 3.2.4.3.1 Strongly supported risk factors

- **Advanced maternal age (≥35 years):** Repeatedly identified as a key predictor of GDM due to age-related metabolic changes (76, 78).
- **Overweight and obesity (BMI ≥25 kg/m²):** High BMI emerged as a major determinant, linked to insulin resistance, especially in urban populations (79–81).
- **Family history of diabetes:** Genetic predisposition was a consistent predictor across West and East African studies (70, 82, 83).
- **Previous obstetric complications:** History of GDM or delivery of a macrosomic infant (>4 kg) increased risk (65, 69).
- **Multiparity and hypertensive disorders of pregnancy** were also frequently reported as risk factors (82, 84).

###### 3.2.4.3.2 Lifestyle and socioeconomic factors

Urban residence reduced physical activity, and dietary transitions toward refined or processed foods were consistently associated with higher GDM prevalence (80, 81). Socioeconomic factors, including limited access to prenatal care and low education, further contributed to risk (62, 82).

###### 3.2.4.3.3 Emerging or region-specific risk factors

- HIV infection and antiretroviral therapy, particularly in Southern Africa (75, 85).
- Polycystic ovary syndrome (PCOS) and short interpregnancy intervals (86).
- Genetic susceptibility in certain ethnic groups (87–92).

The multi-country analysis by Nisar et al. (2024) (76) emphasized early pregnancy glycemic dysregulation as predictive of adverse outcomes, reinforcing the importance of early identification and intervention.

##### 3.2.4.4 Treatment and Management Approaches/Modalities

Management strategies for GDM varied widely across African settings, with notable differences in access to care, availability of medication, and consistency of follow-up.

###### 3.2.4.3.4 Lifestyle modification

Lifestyle change was the foundation of treatment in nearly all studies, emphasizing dietary adjustment, physical activity, and regular glucose monitoring (62, 78, 82). However, several studies highlighted limited access to structured nutritional counselling, particularly in resource-constrained environments (93–95). Adherence also varied due to the cost of recommended foods and cultural dietary practices.

###### 3.2.4.3.5 Pharmacological treatment

Insulin remained the most common pharmacological therapy, especially within tertiary care facilities (79, 80). Metformin use increased in more recent research from East and Southern Africa due to its relative affordability and availability (81). Combination therapy was reported when monotherapy did not achieve adequate glycemic control (94). Challenges to medication adherence, including cost, availability, and travel requirements for clinic visits, were documented across multiple regions (75).

###### 3.2.4.3.6 Models of care and health system capacity

Comprehensive GDM management was more common in urban tertiary centers, whereas rural clinics often lacked essential resources such as glucometers, dieticians, and specialist care. These disparities affected both diagnosis and ongoing management.

###### 3.2.4.3.7 Postpartum follow-up

Few studies reported structured postpartum glucose testing (65). Where documented, follow-up attendance was low, and screening for progression to type 2 diabetes was limited (65). Overall, treatment practices across African studies reflected substantial variation in clinical resources and health system readiness, with lifestyle modification universally emphasized and pharmacological options constrained by cost and availability.

##### 3.2.4.4 Adverse maternal outcomes

Evidence across the included studies showed that GDM was associated with a range of adverse maternal outcomes. Although the magnitude of risk varied by setting, the direction of association was consistent across regions.

**Preeclampsia and gestational hypertension**: Hypertensive disorders were among the most frequently reported complications in women with GDM (76, 82, 83). Several studies identified significant associations with preeclampsia and gestational hypertension, reinforcing their relevance as key maternal risks (76, 82, 83).

**Cesarean delivery**: Higher rates of cesarean section, including both elective and emergency procedures, were observed in GDM pregnancies (78). These findings were reported in studies that linked operative delivery to fetal macrosomia, induction, or concerns regarding labor progression (78).

**Preterm birth**: Preterm delivery was more common among women with GDM, as documented in several East and West African studies (78). Additional evidence from the multi-country analysis by Nisar et al. (2024) (76) showed that elevated HbA1c further increased the likelihood of early birth.

**Polyhydramnios**: Polyhydramnios was identified as a recurrent complication in a subset of women with GDM. Its occurrence was noted in both clinical and population-based studies (80).

**Labor complications associated with macrosomia**: Obstructed labor and induction due to large for gestational age infants were also reported as maternal complications linked to GDM (78).

**Maternal infections**: Although mentioned less extensively, studies identified increased risks of urinary tract and postpartum infections among women with GDM, indicating an additional burden of morbidity, particularly in high-risk clinical environments (96).

Overall, the reviewed evidence demonstrates that GDM consistently increases the likelihood of hypertensive disorders, operative delivery, preterm birth, polyhydramnios, and labor complications across diverse African populations.

##### 3.2.4.5 Adverse neonatal outcomes

Neonatal complications were consistently more frequent among infants born to mothers with GDM. Although the pattern varied by region and clinical capacity, several outcomes appeared repeatedly across the reviewed studies.

**Macrosomia**: Macrosomia was one of the most common complications and was reported across numerous settings (69, 81). Inadequate maternal glycemic control further increased this risk (62).

**Neonatal hypoglycemia and respiratory distress**: Hypoglycemia and respiratory distress were frequently documented in infants exposed to GDM (82). These outcomes were also noted in multi-site studies assessing broader neonatal morbidity (69, 81).

**Shoulder dystocia and birth trauma**: Higher rates of macrosomia contributed to shoulder dystocia and mechanical birth complications, as reported in several cohorts (69, 81, 97–100).

**Admission to neonatal intensive care units**: Infants born to mothers with GDM showed increased likelihood of requiring neonatal intensive care, often due to respiratory challenges, hypoglycemia, or delivery complications (69, 81).

**Stillbirth**: Although less common, stillbirth showed a significant association with GDM in multi country analyses, particularly in settings with limited prenatal surveillance (65, 76, 79).

**Low Apgar scores and hyperbilirubinemia**: Lower Apgar scores and elevated risk of hyperbilirubinemia were also documented, reflecting a broader pattern of neonatal instability in GDM pregnancies (57, 101).

**Neonatal mortality**: Neonatal mortality varied substantially across regions and was highest in settings lacking sufficient neonatal intensive care capacity (65, 79).

Overall, these findings show that GDM is associated with a wide spectrum of adverse neonatal outcomes, many of which are exacerbated by limited clinical resources and challenges in achieving optimal maternal glycemic control.

#### 3.2.5 Regional evidence gaps

The review identified several important gaps in the evidence base for GDM across Africa. Data from Central Africa remained particularly limited, with few studies available from this region. Long term follow up of women after pregnancy was also uncommon, and most studies did not report outcomes related to later progression to type 2 diabetes. High quality cohort studies in rural settings were scarce, restricting understanding of GDM in populations with limited health system access.

Research evaluating the cost effectiveness of screening and diagnostic strategies was minimal, despite considerable variability in practice across countries. Broader gaps were also noted in the study of nutritional interventions, psychosocial factors, and the influence of health system capacity on GDM detection and management.

Overall, these gaps highlight the need for more comprehensive and regionally balanced research to strengthen the evidence base for GDM across the continent.

#### 3.2.6 Overall synthesis

Although the included studies differed in design, diagnostic approaches, and population characteristics, several consistent patterns emerged across the African context. Evidence from multi-country analyses, including the work of Nisar et al. (2024) (76), indicates a rising prevalence of GDM, particularly in rapidly urbanizing areas. Much of this variation reflects differences in diagnostic criteria, which remain a major limitation for comparing results across regions.

Obesity, advanced maternal age, family history of diabetes, and previous adverse pregnancy outcomes were the most consistently reported risk factors. Management practices showed similar trends, with insulin remaining the principal pharmacologic therapy and metformin use increasing in more recent studies. Maternal and neonatal complications continued to pose a considerable burden, particularly where health system capacity was constrained. Limited resources also affected the implementation of universal screening and contributed to inconsistent postpartum follow up. Overall, the available evidence points to an expanding GDM burden across the continent, shaped by both epidemiologic transitions and substantial clinical and system level challenges.

## 4 Discussion of findings

The combined bibliometric and clinical synthesis reveal an expanding but uneven body of scholarship on GDM in Africa. Publication trends mirror growing recognition of GDM as a contributor to maternal and neonatal morbidity, while also reflecting persistent structural, diagnostic and infrastructural disparities across the continent. The bibliometric analysis demonstrates a rapidly expanding but uneven research field characterized by strong contributions from a small number of countries, institutions, and authors, alongside growing international collaboration. These patterns reflect increasing recognition of GDM as an emerging public health challenge linked to demographic transitions, rising obesity, and changes in diet and lifestyle across the continent. At the same time, the systematic review and meta-analysis reveal substantial epidemiological heterogeneity and important clinical implications for maternal and neonatal health. By integrating these complementary perspectives, the study highlights not only the magnitude of the clinical burden but also the structural factors shaping the evidence base. This integrated approach therefore provides a stronger foundation for identifying research priorities, informing policy development, and guiding the implementation of effective screening, prevention, and management strategies for gestational diabetes within African health systems.

### 4.1 Burden of GDM and geographic disparities

A key finding from our analyses is the steady growth in scientific output over the past 25 years, with accelerated expansion after 2015. This increase-parallels heightened awareness of GDM as a public health concern. The thematic map identified GDM, pregnancy, insulin resistance, macrosomia and preeclampsia as central basic themes, while motor themes such as preterm birth, neonatal outcomes and mortality demonstrated high maturity. These patterns align with systematic review findings linking GDM to hypertensive disorders, fetal overgrowth, operative delivery and neonatal complications.

The observed trends are consistent with continental prevalence estimates of approximately 13.6 percent reported by Muche et al. (2019) (102) and corroborated by Natamba et al. (2019) (103), both of whom documented substantial disease burden alongside obesity and adverse obstetric outcomes. Earlier work by Atun et al. (2015) (104) and Macaulay et al. (2014) (13, 17, 63) similarly highlighted pronounced heterogeneity driven by inconsistent diagnostic approaches.

Geographic concentration of research output remains evident. South Africa, Nigeria and Ethiopia dominate publication and collaboration networks, reflecting uneven research capacity. Prevalence estimates varied widely across regions, ranging from 12 to 45.5% in North Africa, 4 to 39% in West and East Africa, 8 to 25% in Southern Africa and 4 to 7% in Central Africa. These variations largely reflect differences in screening strategies, diagnostic thresholds and levels of urbanization. Within-country variation further complicates interpretation, as demonstrated by Dias et al. (2019) (1), who reported substantial differences in screening practices in South Africa. These imbalances suggest that GDM burden is likely underestimated in settings with limited diagnostic resources.

An important finding of this study is the consistently extreme level of heterogeneity observed across the included studies, with I² values exceeding 98% across all analytical periods. Rather than representing merely a statistical limitation, this heterogeneity reflects the structural and methodological diversity of GDM research across Africa. Studies differed substantially in diagnostic criteria, screening strategies, healthcare infrastructure, and population characteristics. In particular, the progressive adoption of IADPSG/WHO 2013 diagnostic thresholds and universal OGTT has been associated with substantially higher detection rates compared with earlier diagnostic frameworks such as WHO 1999 criteria or selective screening approaches. As a result, reported prevalence estimates across African studies ranged widely from very low values in settings with limited screening to substantially higher values in studies employing more sensitive diagnostic criteria. These findings suggest that the wide variation in prevalence estimates may not only reflect true epidemiological differences but also differences in diagnostic implementation and health system capacity across regions. Consequently, the extreme heterogeneity observed in this meta-analysis highlights a critical gap in the African GDM research landscape: the absence of standardized diagnostic practices and consistent screening strategies, which continues to obscure accurate estimation of the true burden of gestational diabetes across the continent.

### 4.2 Metabolic drivers, diagnostic variability and clinical outcomes

Metabolic factors dominate the intellectual structure of GDM research in Africa. Insulin resistance, glucose intolerance and obesity consistently emerged as high centrality themes, reinforcing the prominence of metabolic pathways. Conceptual mapping confirmed tight clustering of these terms with GDM, echoing findings by Muche et al. (2019) (102) and Natamba et al. (2019) (103), who identified overweight, family history of diabetes, previous macrosomia and prior GDM as key risk factors.

Clinical outcomes such as macrosomia, preterm birth, neonatal morbidity and perinatal mortality remain well developed themes, supported by evidence from Natamba et al. (2019) (103), Abera et al. (2024) (105), Chileshe et al. (2025) (106), Sweeting et al. (2024) (107) and Gadhia and Loyal (2024) (108). However, the diagnostic landscape remains fragmented. Studies employ diverse criteria, including WHO and IADPSG thresholds, often with local modifications. Dias et al. (2019) (1) emphasized that this variability substantially affects prevalence estimates and limits synthesis. Mwanri et al. (2015) (109) and Putoto et al. (2020) (110) further noted that reliance on externally developed diagnostic frameworks often overlooks local constraints such as limited laboratory capacity and delayed antenatal attendance.

Furthermore, the substantial heterogeneity observed across the pooled estimates is also better understood when interpreted alongside the bibliometric findings of this study. The bibliometric analysis demonstrated that GDM research in Africa is geographically concentrated, with South Africa, Nigeria, and Ethiopia accounting for a disproportionate share of the published literature. This uneven research distribution likely contributes to the heterogeneity identified in the meta-analysis, as the epidemiological and clinical characteristics of these countries may not fully represent the diversity of healthcare systems, screening practices, and population risk profiles across the continent. Consequently, pooled prevalence estimates and reported risk factors may reflect the dominant methodological approaches and diagnostic criteria used in these research-intensive settings rather than a uniform continental pattern. Furthermore, the bibliometric mapping revealed a relative scarcity of health-systems and implementation research, which may partly explain the considerable variability in screening strategies, diagnostic thresholds, and reporting practices observed across the included studies. Differences in the adoption of diagnostic guidelines – such as WHO, IADPSG, or locally adapted criteria – as well as variation in universal versus risk-based screening approaches, likely contribute to the wide range of prevalence estimates and clinical outcomes reported in the literature. Taken together, these findings suggest that the heterogeneity identified in the meta-analysis is not solely a statistical phenomenon but also reflects structural characteristics of the African GDM research landscape, including geographic research concentration, methodological diversity, and limited system-level studies examining implementation of diagnostic and management protocols. Integrating the bibliometric perspective therefore provides important context for interpreting the variability in the clinical evidence base and highlights the need for more geographically diverse, standardized, and health-system-focused research to improve the comparability and generalizability of GDM evidence across Africa.

### 4.3 Translational gaps and public health implications

Despite strong biomedical and clinical outputs, public health and health systems research remains underrepresented. Maternal medicine, implementation science and community-based prevention appear in low density thematic regions, indicating limited translational focus. This gap is consistent with concerns raised by Muche et al. (2019) (102) and Natamba et al. (2019) (103), particularly regarding inadequate postpartum follow up despite well-established long term diabetes risk.

Geographic clustering around South Africa, Tanzania and Ethiopia further underscores disparities in research infrastructure. Expanding multi-country collaboration and investing in underrepresented regions are essential to improve representativeness. Overall, while GDM research in Africa is growing rapidly, it remains conceptually fragmented, with strong clinical insights but limited integration into health systems and policy frameworks.

From a policy perspective, the findings highlight several priorities for strengthening maternal metabolic health research and care in Africa. First, the concentration of research activity in a limited number of countries underscores the need for greater investment in research capacity across underrepresented regions, particularly in Central and parts of West Africa. Second, the strong emphasis on prevalence and diagnostic studies suggests that future work should increasingly focus on evaluating effective prevention strategies, postpartum diabetes surveillance, and long-term maternal and offspring outcomes. Third, the reliance on international diagnostic frameworks highlights the importance of developing contextually appropriate screening and management guidelines that reflect local epidemiology, resource constraints, and health system capacities. Addressing these gaps will be essential for translating research evidence into effective clinical practice and public health policy aimed at reducing the growing burden of gestational diabetes and its intergenerational consequences across the continent.

## 5 Limitations and research gaps in GDM research in Africa

Bibliometric findings highlight persistent inequities in research capacity, with publication output concentrated in a small number of countries and institutions. Large regions remain underrepresented, limiting generalizability. Diagnostic inconsistency, reliance on external guidelines and infrastructural constraints further restrict comparability across studies. Collaboration networks reveal fragmentation, with limited cross-country partnerships and overreliance on a small group of recurring authors.

The thematic structure also shows overemphasis on descriptive epidemiology and short-term outcomes, while postpartum follow up, intergenerational effects, culturally adapted interventions and implementation research remain underdeveloped. Addressing these gaps will require harmonized diagnostic criteria, stronger regional networks and sustained investment in longitudinal and population-based research tailored to African contexts.

## 6 Conclusion

The emerging landscape of GDM research in Africa, as demonstrated through the bibliometric and systematic review analysis, reveals a field that is expanding in both scope and scientific depth, yet still marked by significant structural and thematic gaps that limit its full potential. While publication output has increased steadily over the past decade and has illuminated important metabolic, clinical and epidemiological dimensions of GDM across several African countries, the concentration of research within a small cluster of institutions and regions continues to restrict the generalizability of existing evidence. Diagnostic inconsistency, limitations in collaborative networks, insufficient postpartum follow up, and the underrepresentation of health system and implementation focused research collectively underscore the need for a more cohesive, context driven and methodologically harmonized research agenda. Strengthening intra African collaborations, investing in research infrastructure, and developing diagnostic and management strategies that reflect the realities of diverse African health systems will be essential to advancing knowledge and translating evidence into sustainable maternal and child health improvements.

Taken together, the findings of this study suggest that the apparent variability in reported GDM prevalence across Africa is driven not only by underlying epidemiological differences but also by inconsistent diagnostic practices, heterogeneous screening strategies, and uneven research coverage across regions. The extreme heterogeneity observed in this meta-analysis therefore highlights a critical public health challenge for accurately estimating the burden of GDM on the continent. Without greater standardization of diagnostic criteria, wider implementation of universal screening strategies, and improved research representation across under-studied regions, the true epidemiological profile of GDM in Africa will remain difficult to quantify. Strengthening surveillance systems, harmonizing diagnostic guidelines, and expanding research capacity across diverse African health systems will be essential for improving early detection, informing maternal health policy, and reducing the long-term metabolic risks associated with untreated gestational diabetes.

## 7 Study limitations

This study provides a comprehensive synthesis of GDM research in Africa through the combined application of bibliometric analysis and systematic review with meta-analysis. Nevertheless, several limitations should be considered when interpreting the findings. First, although the primary literature search relied on the Scopus and Web of Science databases, which are widely recognized for their rigorous indexing standards and comprehensive citation coverage, this may still limit representation of some locally disseminated research outputs. To minimize potential database-related selection bias, additional verification searches were conducted in PubMed and Google Scholar using Harzing’s Publish or Perish software during the initial search phase. These supplementary searches confirmed that all eligible peer-reviewed journal articles identified in these databases were already indexed in either Scopus or Web of Science, while additional records detected outside these databases primarily consisted of theses, dissertations, conference abstracts, or other forms of gray literature, which fell outside the predefined inclusion criteria of this review.

Second, the review included only English-language publications, reflecting the language proficiency of the research team and ensuring accurate interpretation, extraction, and synthesis of study findings. While this approach ensured methodological reliability, it may have excluded some research published in other languages, particularly from Francophone and Lusophone regions of Africa, which could contribute to geographic imbalances in the observed research landscape.

Third, the meta-analysis revealed extremely high heterogeneity across studies, reflecting substantial variability in study design, diagnostic criteria, screening strategies, and population characteristics across African settings. Differences between diagnostic frameworks – such as WHO 1999, WHO 2013/IADPSG, ADA, NICE, and Carpenter-Coustan criteria – as well as variation in screening approaches (e.g., universal versus selective testing) limit direct comparability of prevalence estimates across studies. In addition, some studies reported multiple diagnostic methods within the same study, further complicating attempts to stratify analyses by diagnostic framework. As a result, pooled prevalence estimates should be interpreted primarily as descriptive summaries of the distribution of reported values rather than precise estimates applicable to specific populations.

Fourth, although methodological quality assessment was conducted using established appraisal tools appropriate to each study design, many included studies – particularly cross-sectional and hospital-based prevalence studies – exhibited moderate methodological limitations, including limited adjustment for confounders, small sample sizes, and incomplete reporting of laboratory procedures. Sensitivity analyses excluding lower-quality studies showed minimal influence on the overall findings; however, the predominance of observational and single-institution studies remains an inherent limitation of the current evidence base.

Finally, bibliometric analysis inherently focuses on quantitative patterns of publication, collaboration, and thematic development, which cannot fully capture the qualitative depth, contextual relevance, or policy impact of individual studies. In addition, the rapidly evolving nature of GDM research in Africa means that recent publications may not yet have accumulated substantial citation counts, potentially underestimating their emerging influence within the research landscape.

Despite these limitations, the combined bibliometric and clinical synthesis presented in this study provides an important overview of the structure, scope, and epidemiological variability of GDM research in Africa, while also highlighting critical gaps in diagnostic standardization, regional research coverage, and long-term clinical outcomes that warrant further investigation.

## Data Availability

The dataset contains data extracted from previously published studies and bibliometric sources. Access is limited to the curated extraction files and summary data used in the analyses. Some data may be restricted due to copyright or licensing limitations from the original publications. Researchers requesting access must ensure proper citation of the original sources and comply with any usage restrictions imposed by the publishers. Individual-level patient data are not included, so privacy concerns are minimal, but use is restricted to research, educational, or methodological purposes. Requests to access these datasets should be directed to 22384725@dut4life.ac.za.
