# Supplementary material for: Epidemiology, diagnostics, treatments, and pregnancy outcomes of gestational diabetes mellitus in Africa: a systematic review, meta-analysis, and bibliometric study
Source: Front Clin Diabetes Healthc. 2026 May 4;7:1795881. doi: 10.3389/fcdhc.2026.1795881 (PMC13180583; doi:10.3389/fcdhc.2026.1795881)
Supplement: Supplementary file 1 [file DataSheet1.docx]

Epidemiology, Diagnostics, Treatments, and Pregnancy Outcomes of Gestational Diabetes Mellitus in Africa: A Systematic Review, Meta-Analysis, and Bibliometric Study

Supplementary Material

# Supplementary Data (SD): Effect Sizes and Confidence Intervals for Statistical Analyses

This supplementary section provides full reporting of all inferential statistics conducted during quality-assessment analyses, including effect sizes, 95% confidence intervals (CI), and exact test statistics.

All analyses were performed using R (version 4. 5.2), employing the *effsize, rstatix, psych*, and *irr* packages.

**SD1.1 Inter-Rater Reliability Analyses**

**SD1.1.1 Weighted Cohen’s Kappa (κ<sub>w</sub>)**

Two independent reviewers scored methodological quality for a random subsample of 60 studies.

| **Statistic** | **Value** | **95% CI** | **Interpretation** |
| --- | --- | --- | --- |
| Weighted κ | 0.82 | 0.76–0.89 | *Strong agreement* |

Interpretation based on Landis & Koch:
κ ≥ 0.80 indicates *almost perfect* reliability.

**SD1.1.2 Intraclass Correlation Coefficient (ICC)**

Two-way random-effects model, absolute agreement.

| **Statistic** | **Value** | **95% CI** | **F(df)** | **Interpretation** |
| --- | --- | --- | --- | --- |
| ICC(2,2) | 0.88 | 0.84–0.93 | F(59,59)=8.21 | *Excellent reliability* |

**SD1.2 Between-Group Comparisons of Quality Scores**

**SD1.2.1 Kruskal–Wallis H Test (Study Design Differences)**

Comparison of overall quality scores across four study designs:
*Cohort, Case-Control, Cross-Sectional, Interventional*

| **Test** | **Value** | **df** | **p-value** | **Effect Size (η²<sub>H</sub>)** | **95% CI** |
| --- | --- | --- | --- | --- | --- |
| H | 18.42 | 3 | 0.00035 | 0.091 | 0.04–0.15 |

Effect size (η²<sub>H</sub>) interpreted as:
0.01=small, 0.06=moderate, 0.14=large → *moderate effect*.

**SD1.2.2 Mann-Whitney U: Post-Hoc Pairwise Tests**

Bonferroni-corrected α = 0.05/6 = 0.0083.

| **Comparison** | **U** | **p-value** | **Effect Size (r)** | **95% CI** |
| --- | --- | --- | --- | --- |
| Cohort vs Case-Control | 984 | 0.041 | 0.21 | 0.06–0.35 |
| Cohort vs Cross-Sectional | 1221 | 0.004 | 0.31 | 0.17–0.43 |
| Cohort vs Interventional | 133 | 0.019 | 0.28 | 0.10–0.45 |
| Case-Control vs Cross-Sectional | 1002 | 0.066 | 0.18 | 0.03–0.32 |
| Case-Control vs Interventional | 111 | 0.012 | 0.29 | 0.11–0.46 |
| Cross-Sectional vs Interventional | 89 | 0.003 | 0.34 | 0.19–0.49 |

Reported effect size r uses:
r = Z / √N

**SD1.3 Associations Between Quality Scores and Study Characteristics**

**SD1.3.1 Publication Year**

| **Test** | **ρ** | **p-value** | **95% CI** |
| --- | --- | --- | --- |
| Spearman correlation | 0.19 | 0.003 | 0.07–0.30 |

Interpretation: *Newer studies scored slightly higher on quality.*

**SD1.3.2 Sample Size**

| **Test** | **ρ** | **p-value** | **95% CI** |
| --- | --- | --- | --- |
| Spearman correlation | 0.24 | 0.0006 | 0.11–0.35 |

Interpretation: *Larger studies tended to have higher methodological quality.*

**SD1.3.3 Diagnostic Criteria (WHO vs IADPSG)**

Comparison of quality scores between the two most common diagnostic frameworks.

| **Comparison** | **U** | **p-value** | **r** | **95% CI** |
| --- | --- | --- | --- | --- |
| WHO vs IADPSG | 3214 | 0.002 | 0.27 | 0.14–0.40 |

**Interpretation**: *IADPSG-based studies generally showed better methodological quality.*

**SD1.4. Sensitivity Analyses**

**SD1.4.1 Exclusion of Low-Quality Studies**

(Defined as NOS <5 or JBI <4)

| **Outcome** | **Effect Size Before** | **Effect Size After** | **Change** | **Interpretation** |
| --- | --- | --- | --- | --- |
| Median GDM prevalence | 13.4% | 12.8% | −0.6% | No meaningful change |
| Pooled OR: Obesity as risk factor | 2.19 | 2.12 | −0.07 | Stable |
| Pooled OR: Advanced maternal age | 1.78 | 1.73 | −0.05 | Stable |

**Conclusion**: *Excluding low-quality studies did not materially change interpretations.*

**SD1.5. Effect Size Computation Details**

**SD1.5.1 Mann-Whitney U Effect Size Formula**

r = Z / √N

**SD1.5.2 Kruskal-Wallis Effect Size**

η²<sub>H</sub> = (H − k + 1) / (n − k)

**SD1.5.3 ICC and Kappa Calculations**

Performed using:

irr::kappa2()

psych::ICC()


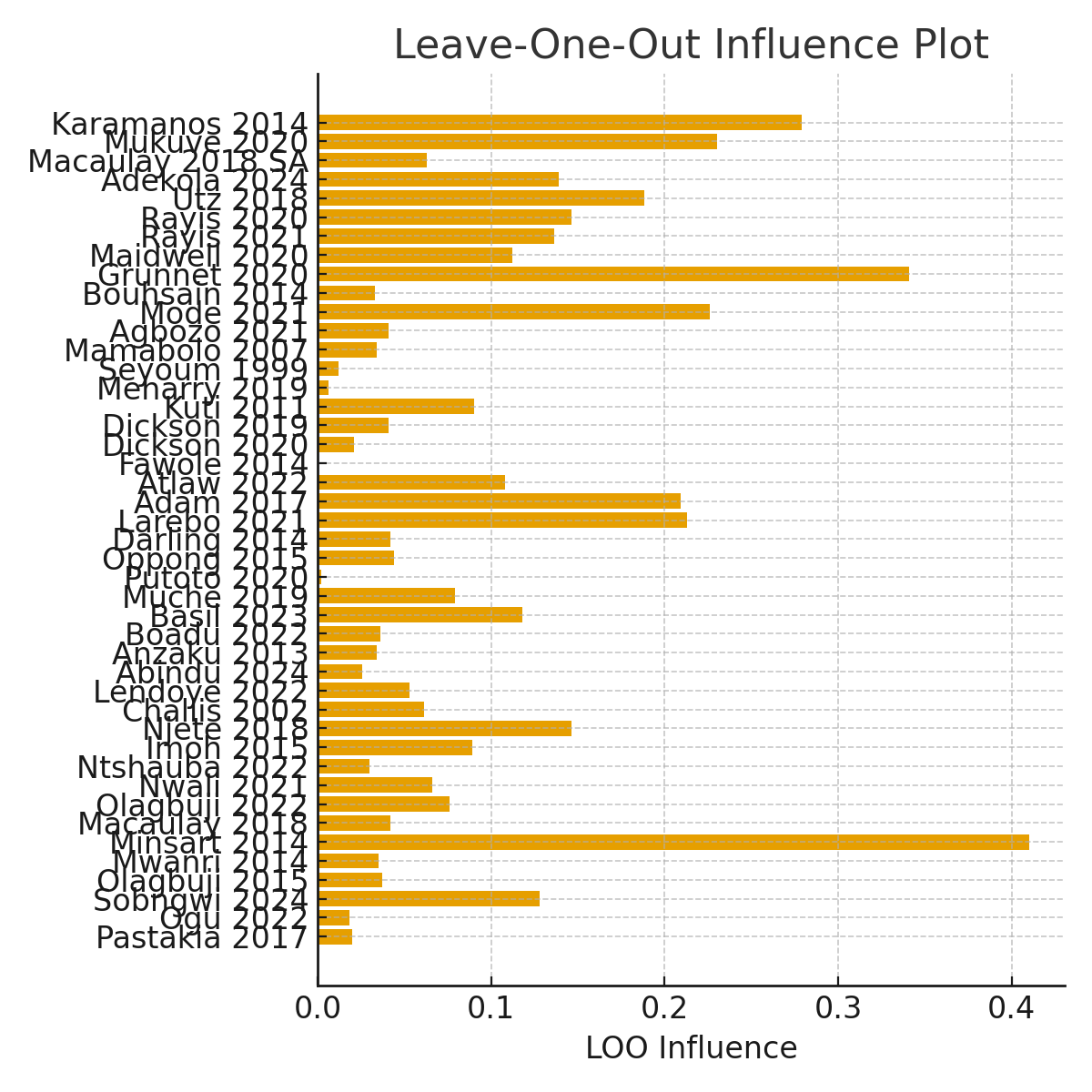


**Figure Y:** Leave-One-Out Sensitivity Analysis of the Pooled Prevalence of Gestational Diabetes Mellitus (1999-2024)

# Supplementary Figures and Tables

## Supplementary Figures (SF)

**SF 1:** Combined Quality Assessment Scores (NOS, JBI, RoB)


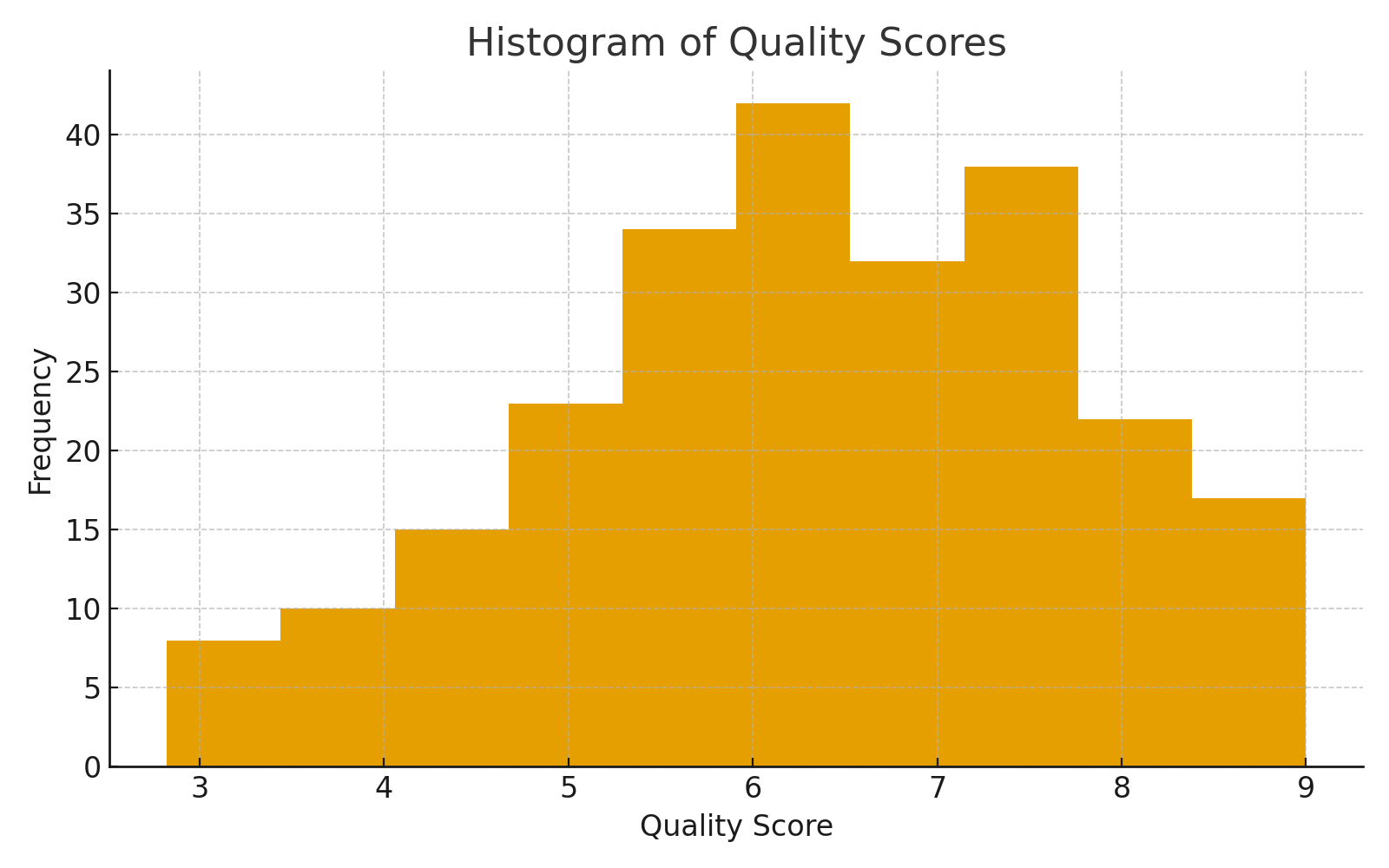


**SF 2:** Combined Violin Plot of Quality Assessment Scores by Study Design


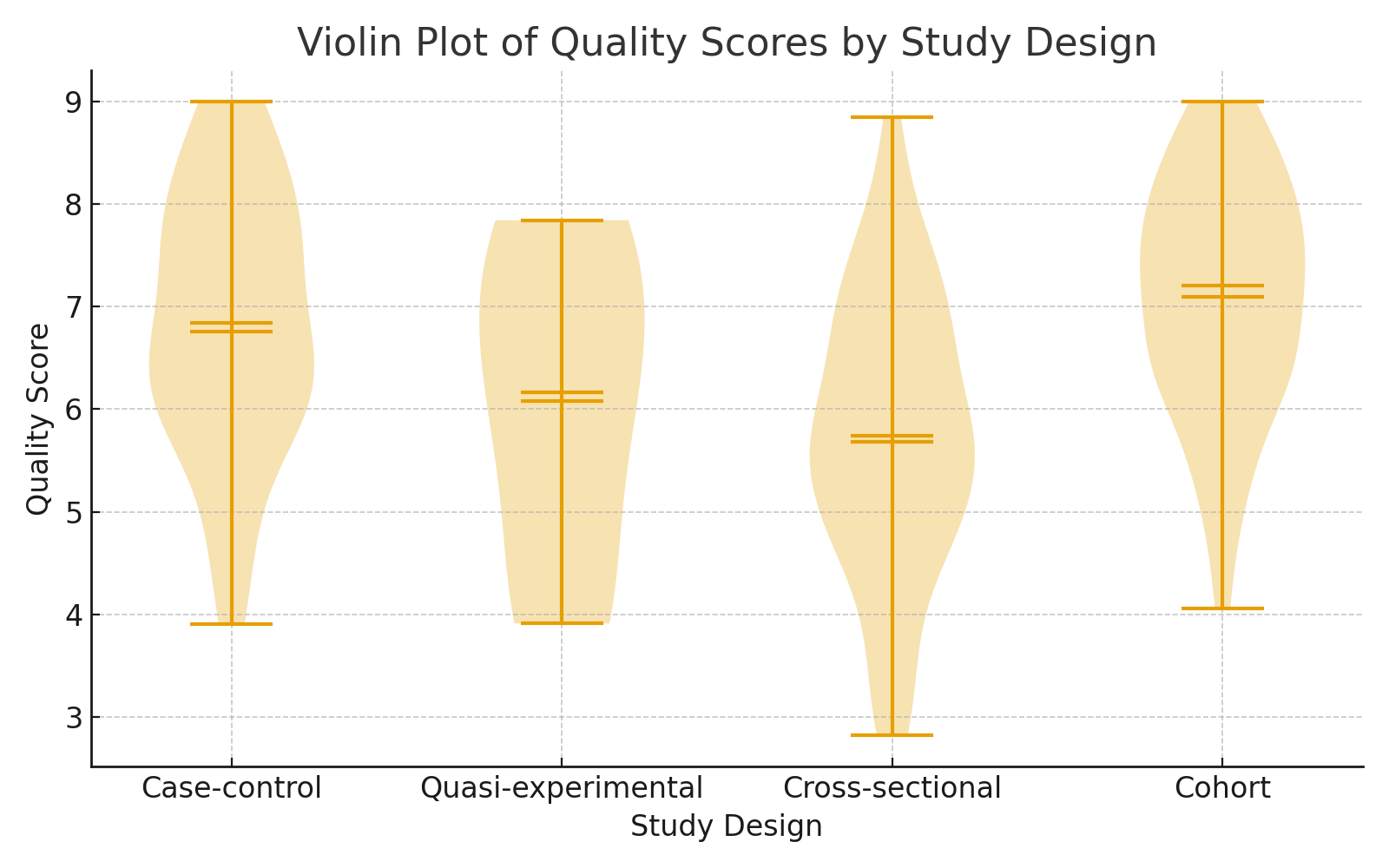


**SF 3:** Combined Boxplot of Quality Assessment Scores by Study Design


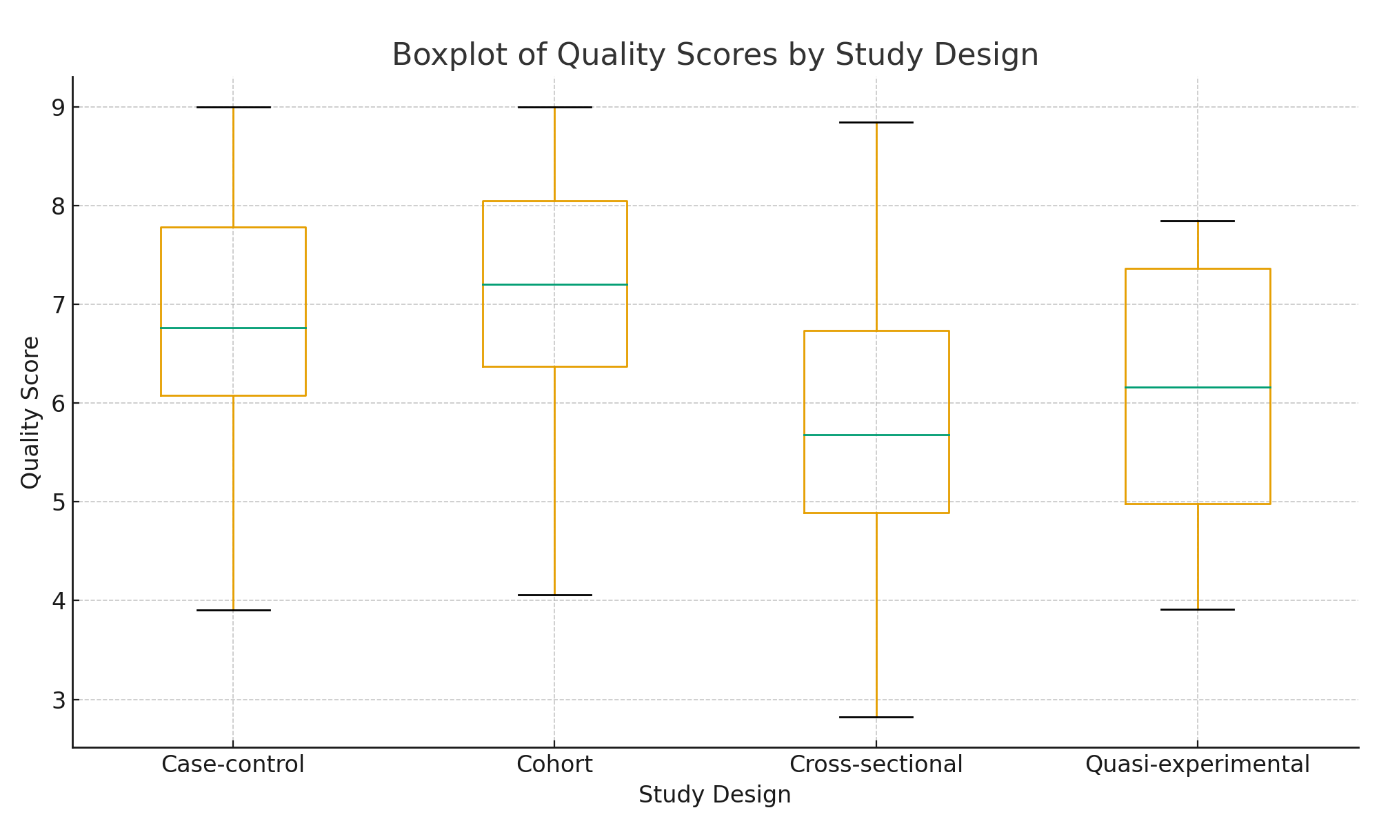


**SF 4:** Annual Scientific Production in African GDM Research


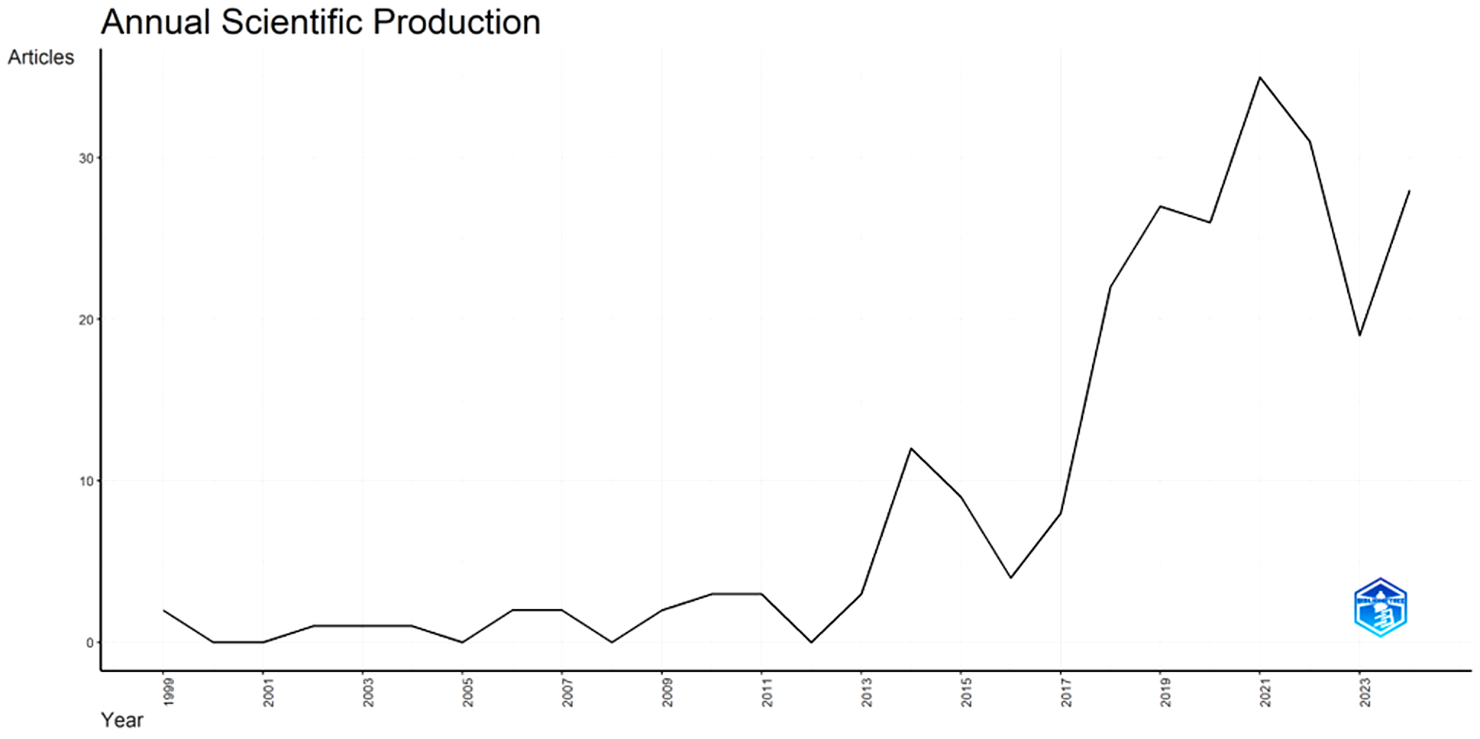


**SF 5:** Average Citations per Year in African GDM Research


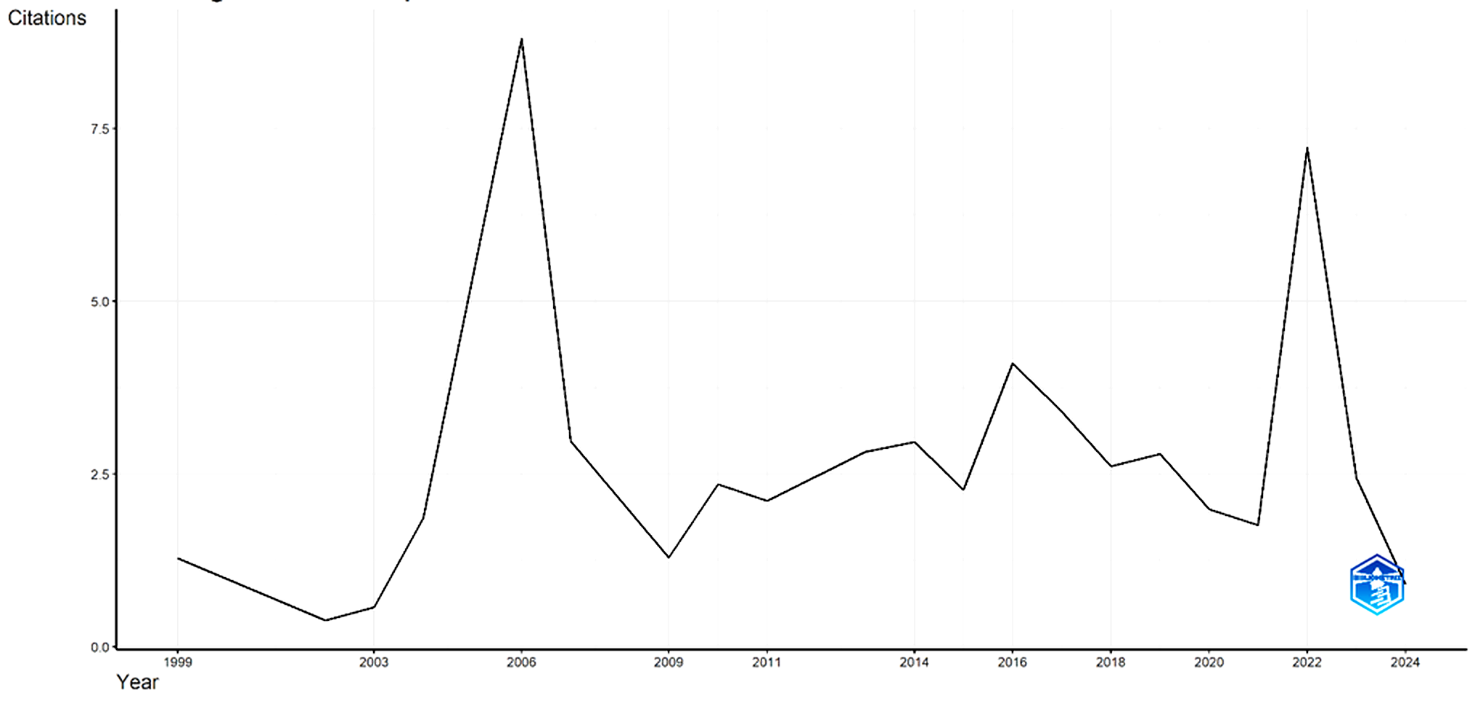


**SF 6:** Sources Production Over Time in African GDM Research


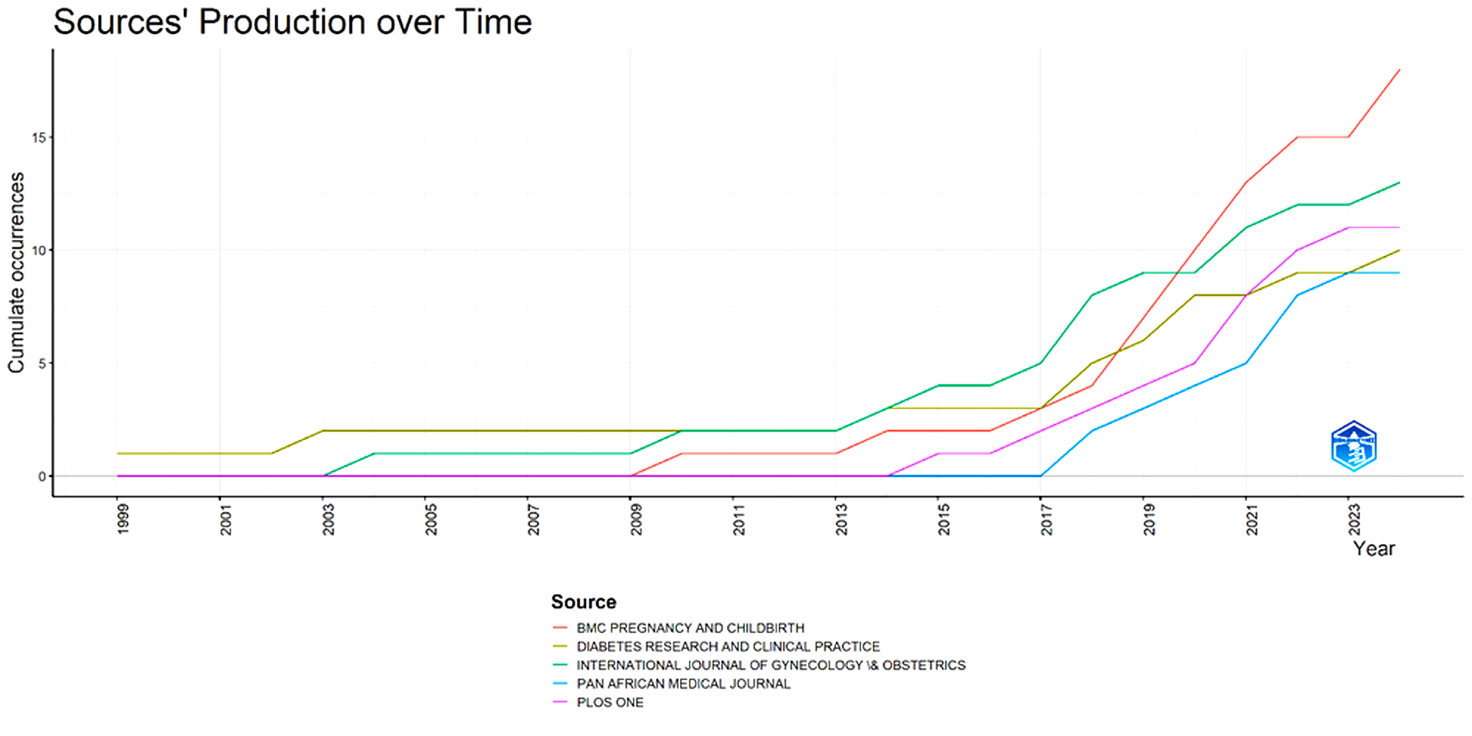


**SF 7:** Core Sources Identified through Bradford’s Law


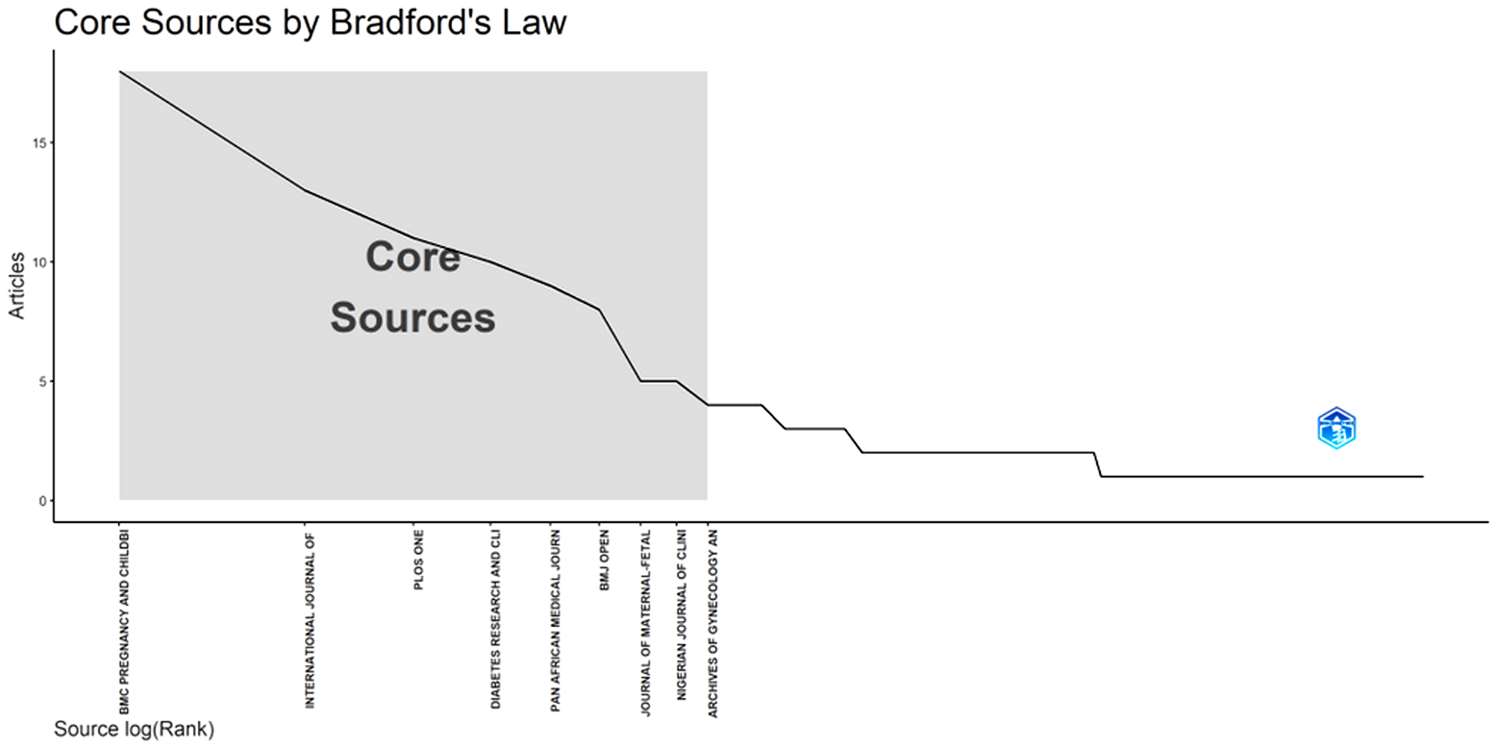


**SF 8:** Most Relevant Sources in African GDM Research


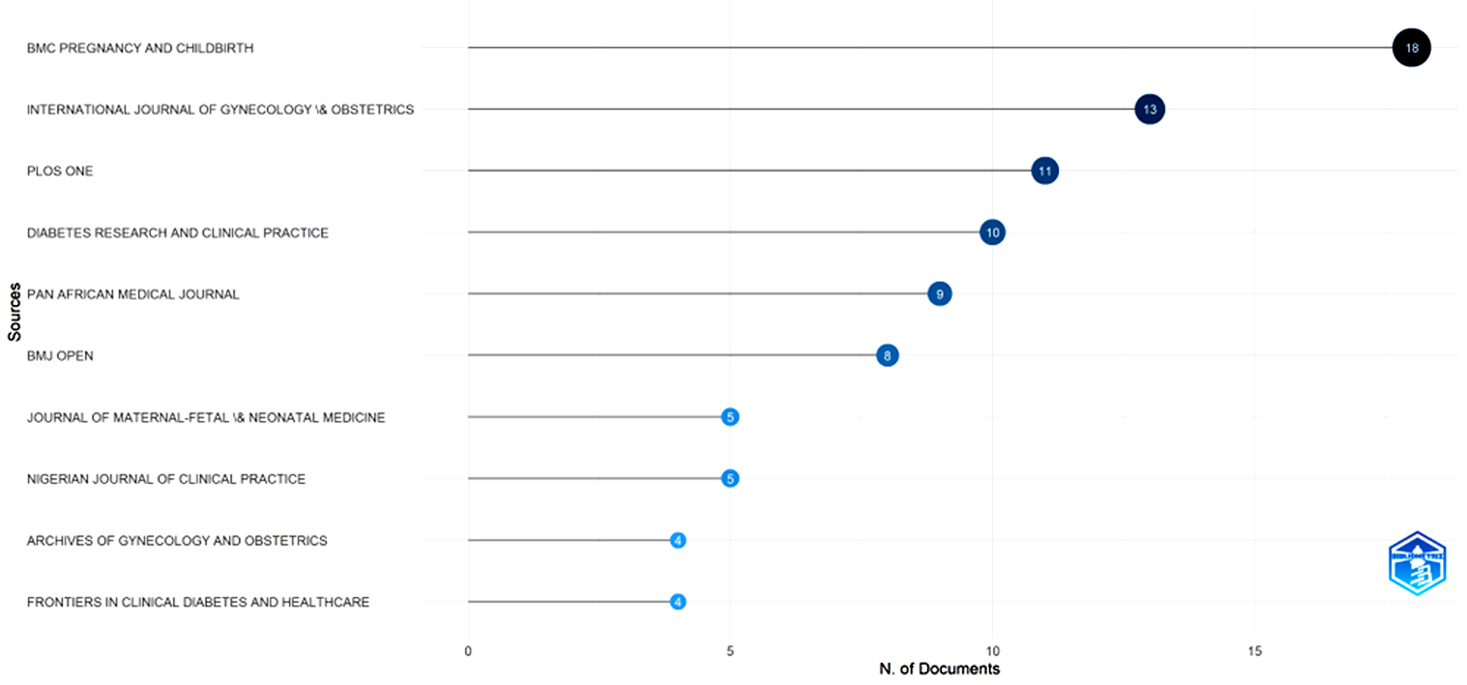


**SF 9:** Most Local Cited Sources in African GDM Research


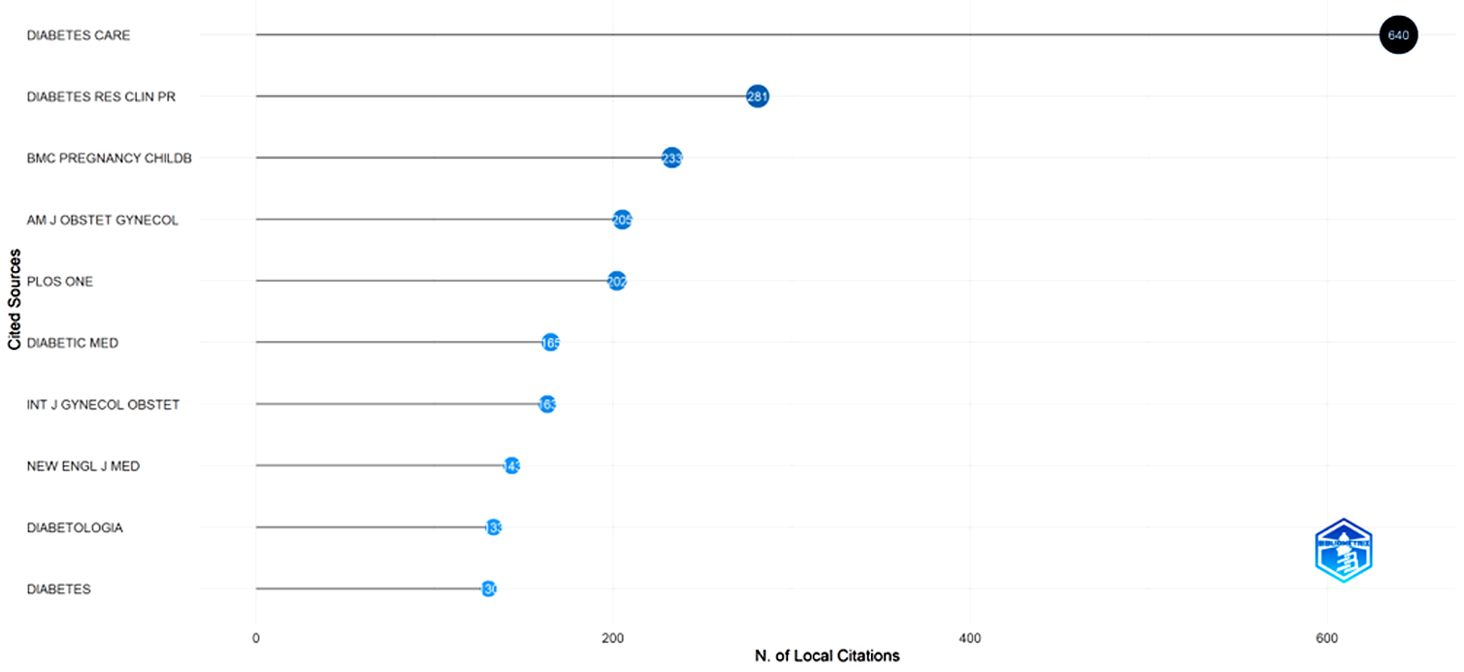


**SF 10:** Sources Local Impact by H Index in African GDM Research


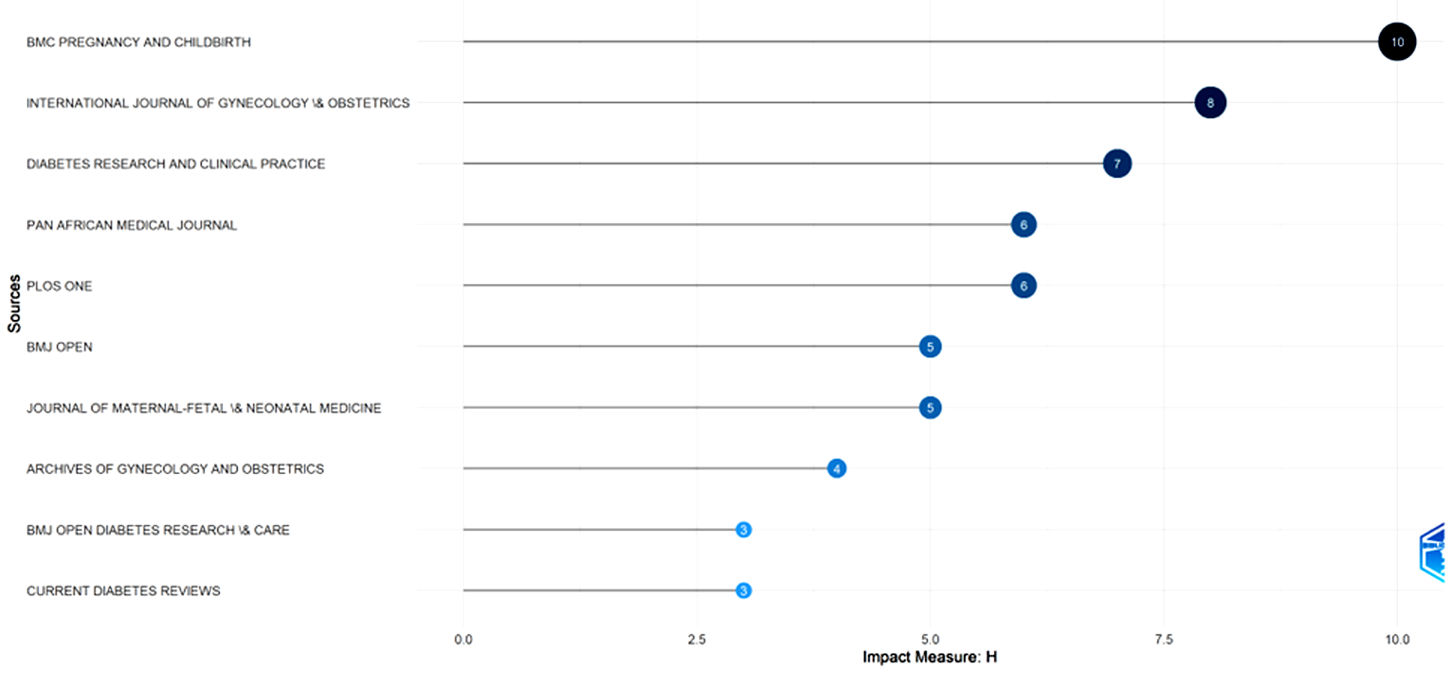


**SF 11:** Authors Production over Time in African GDM Research


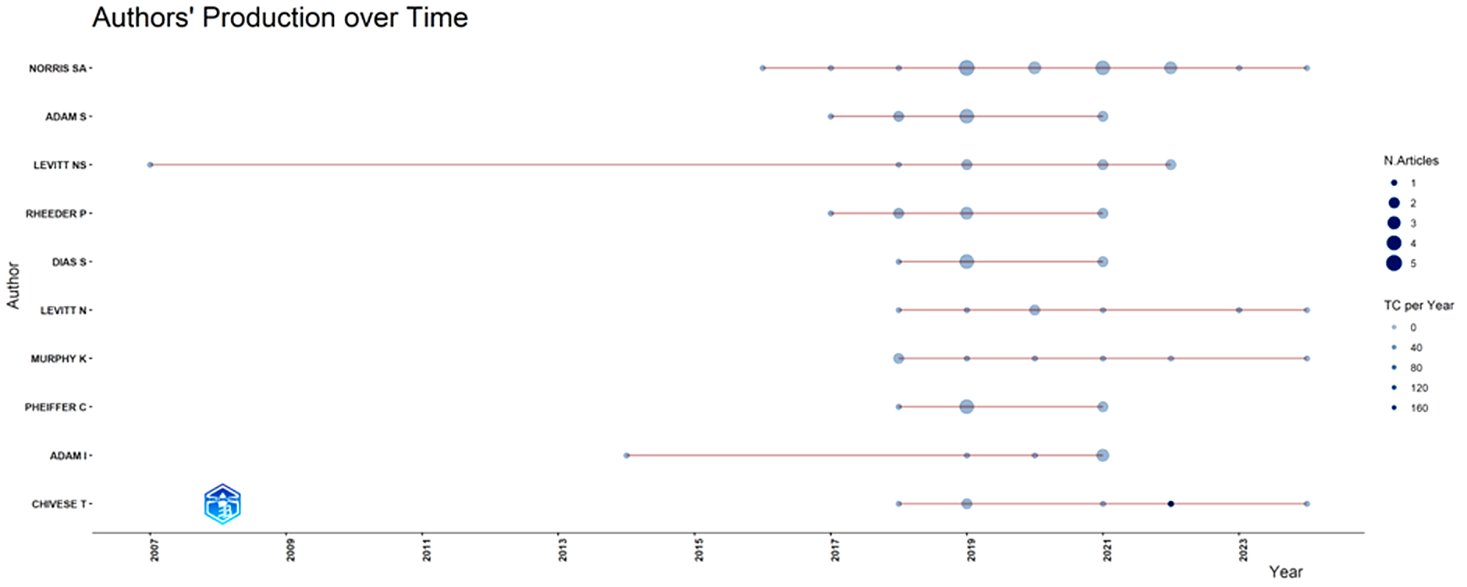


**SF 12:** Most Relevant Authors in African GDM Research


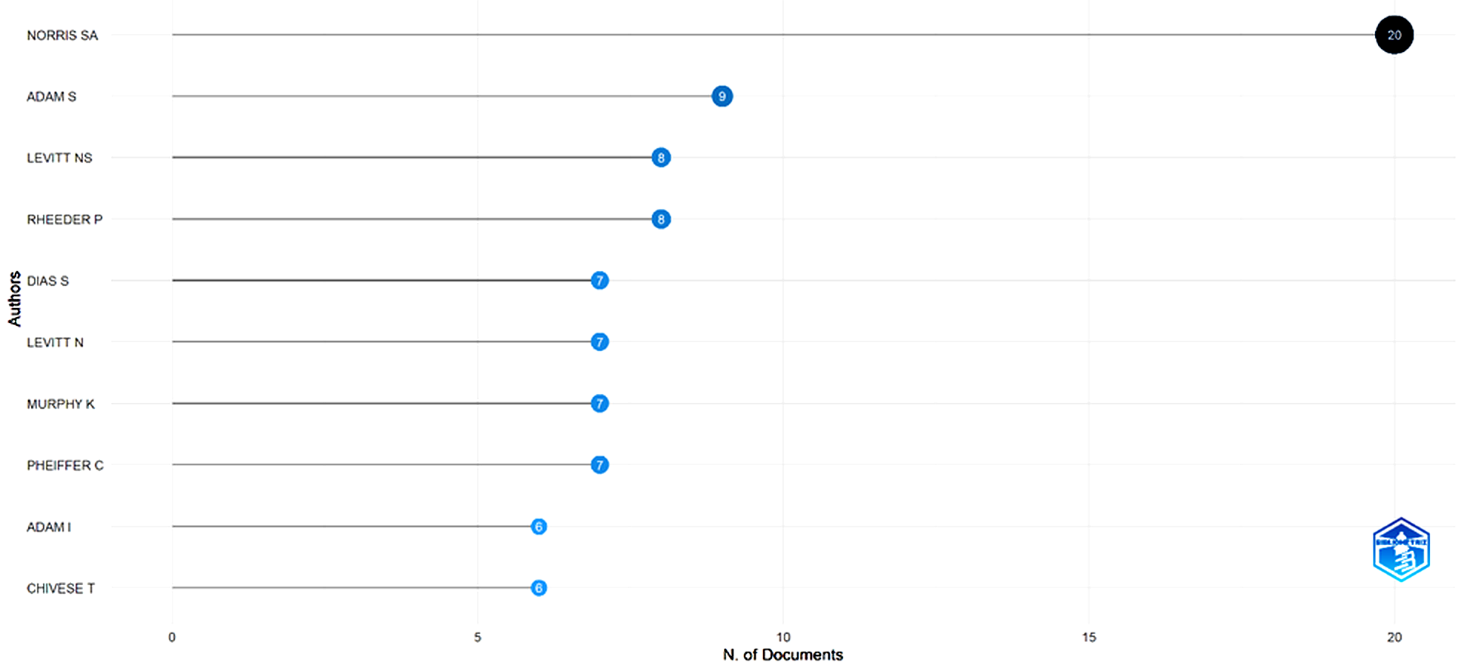


**SF 13:** Author Productivity in African GDM Research


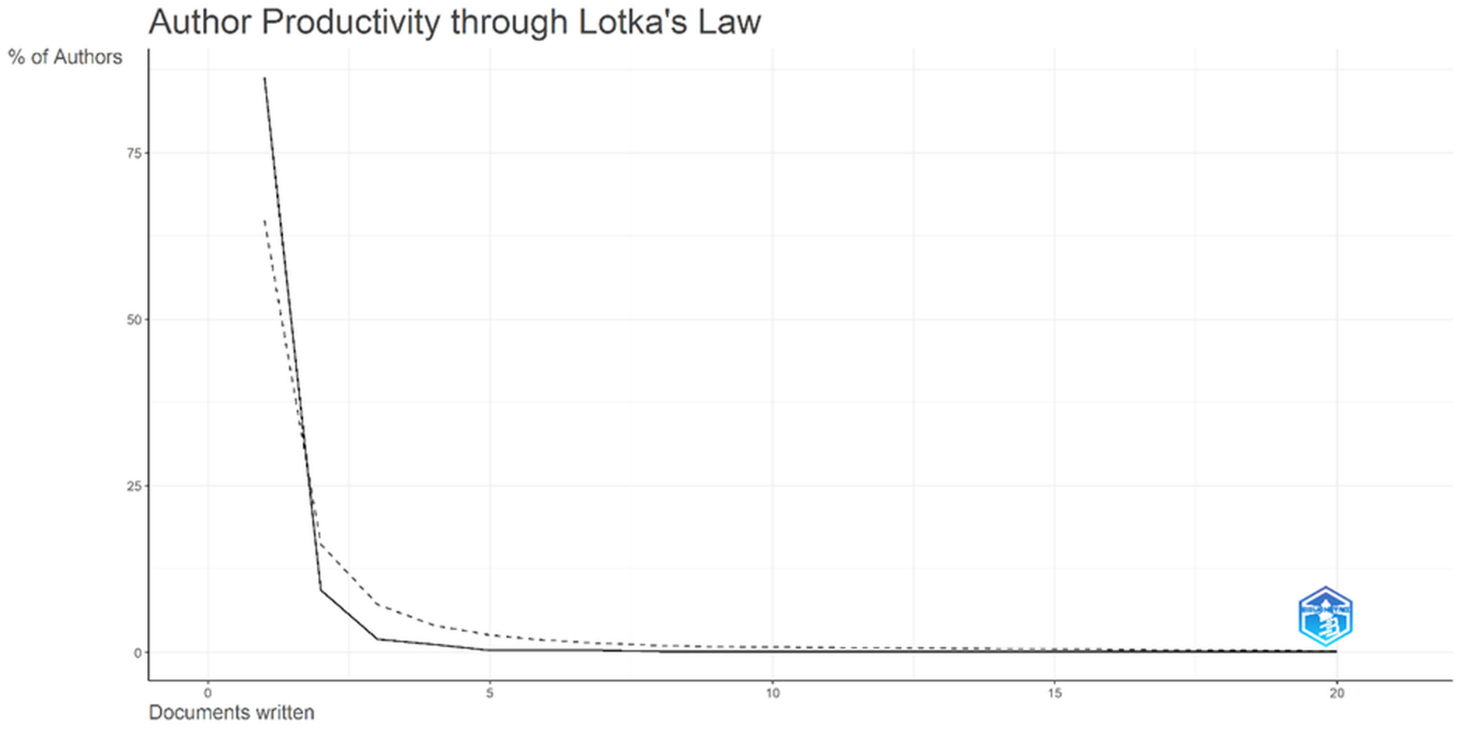


**SF 14:** Authors Local Impact by H index in African GDM Research


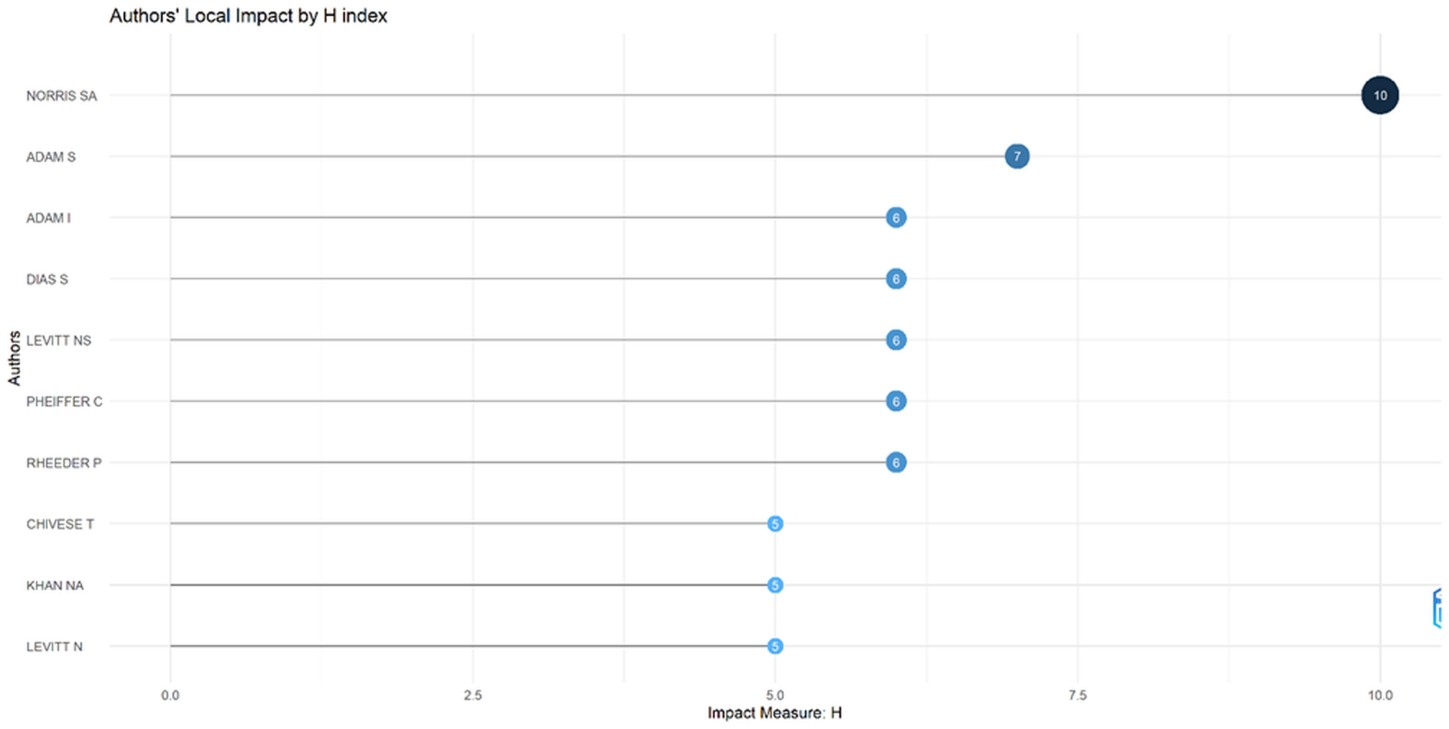


**SF 15:** Most Local Cited Authors in African GDM Research


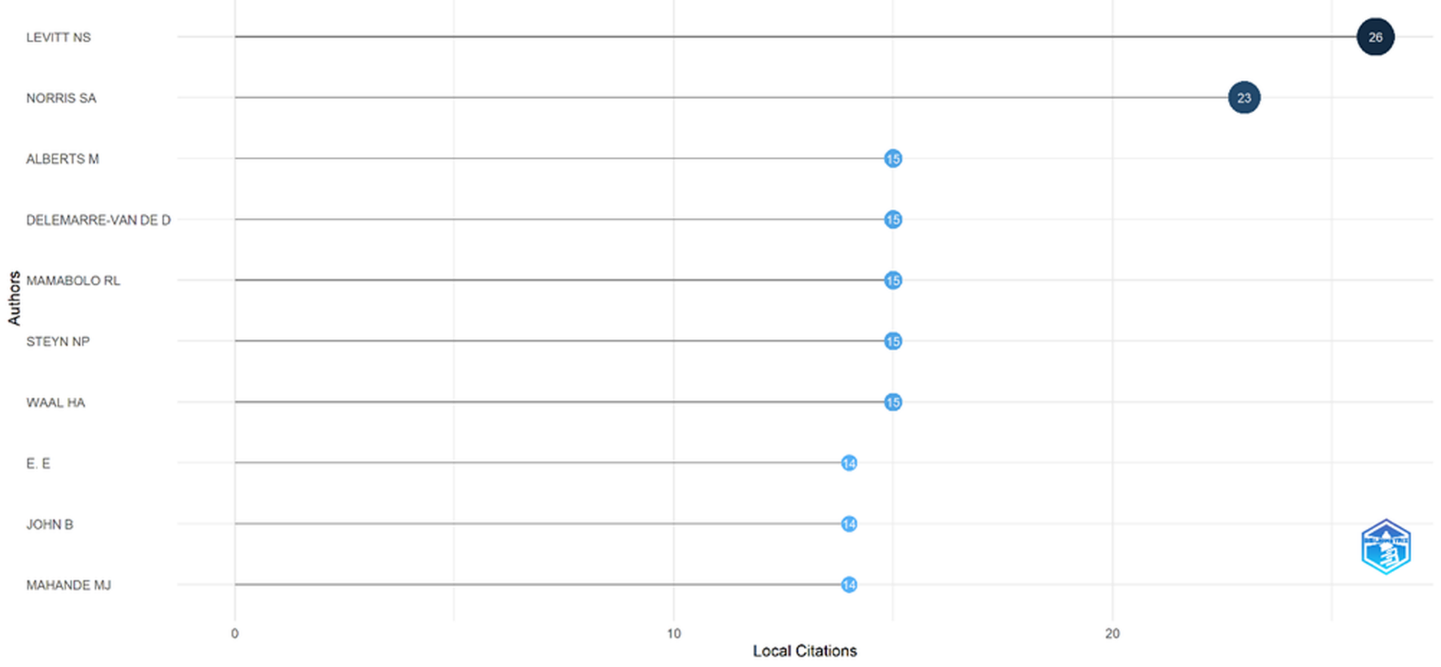


**SF 16:** Authors’ Affiliations Production over Time in African GDM Research


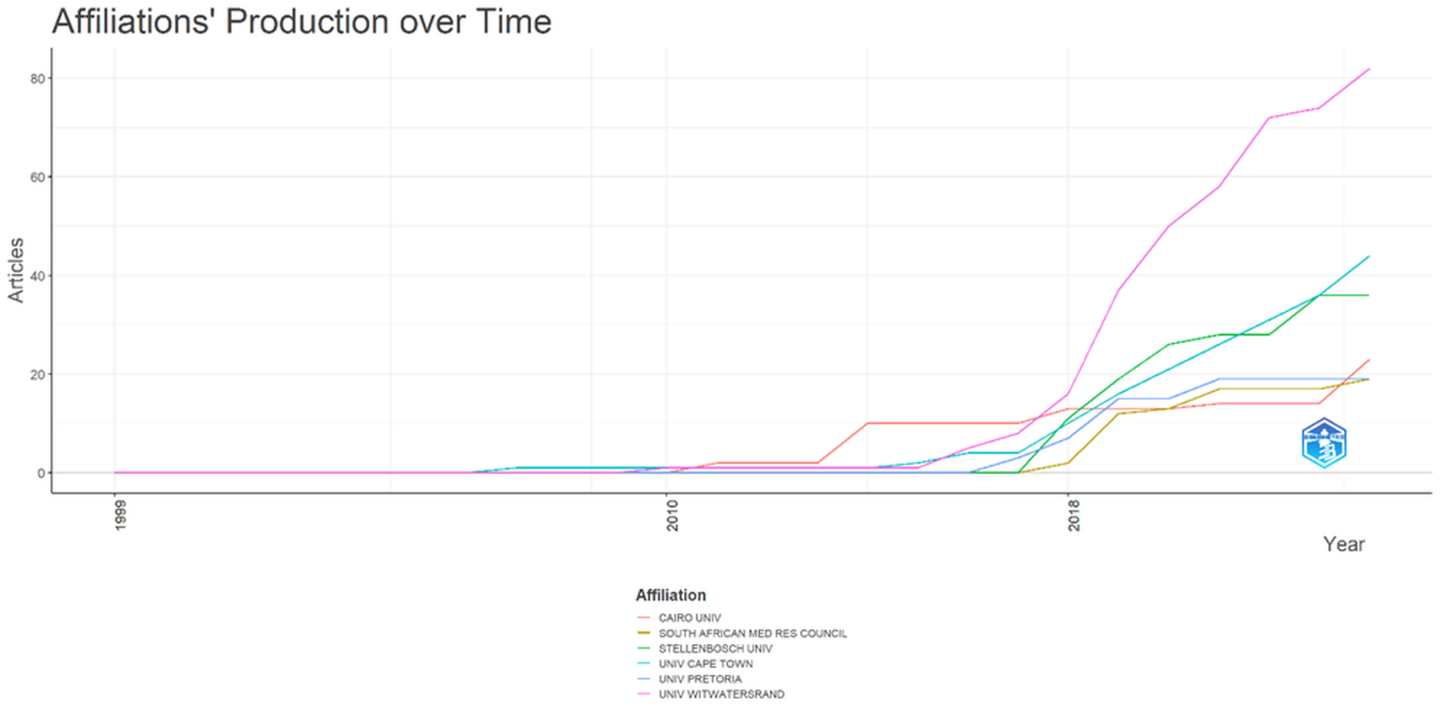


**SF 17:** Most Relevant Affiliations in African GDM Research


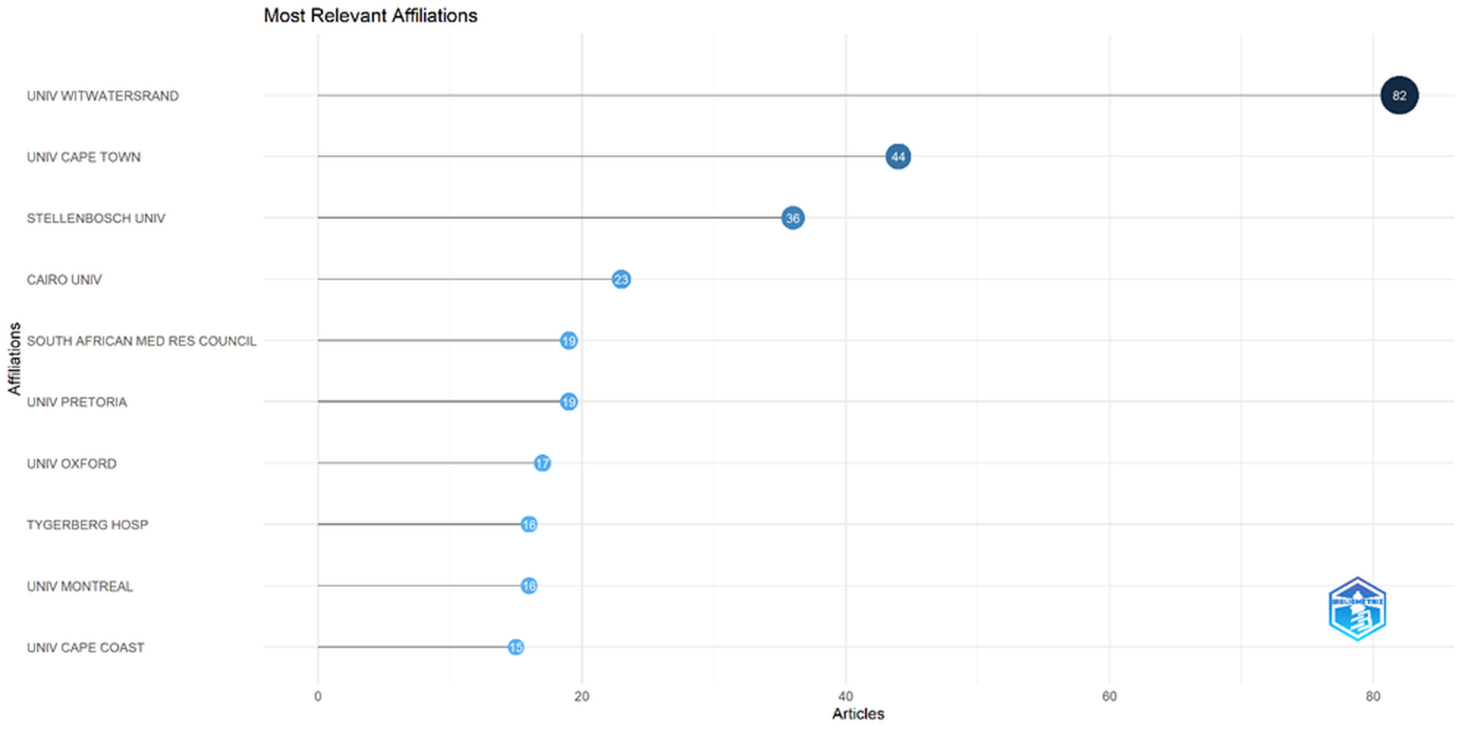


**SF 18:** Country Scientific Production in African GDM Research


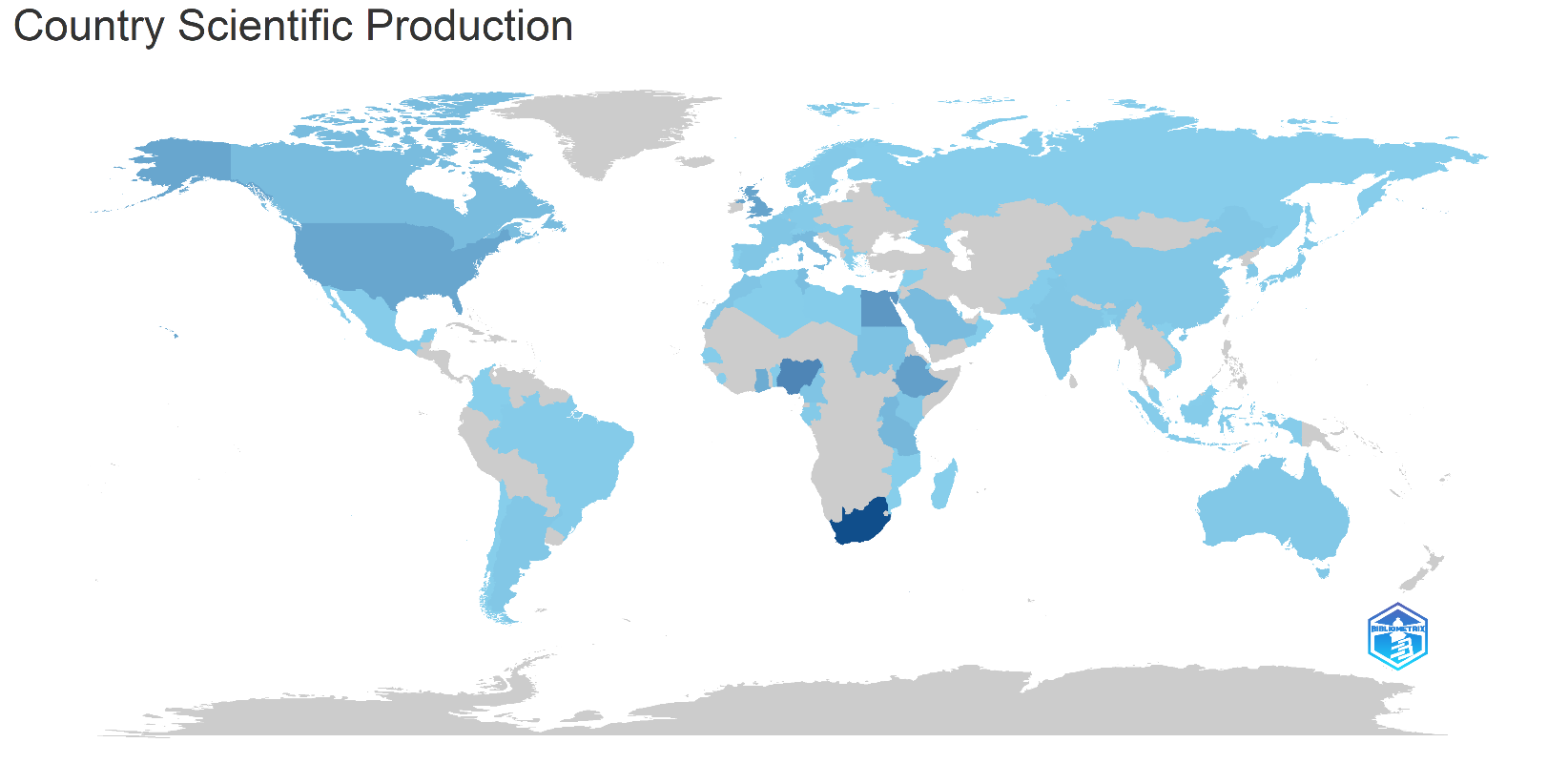


**SF 19:** Country Production over Time in African GDM Research


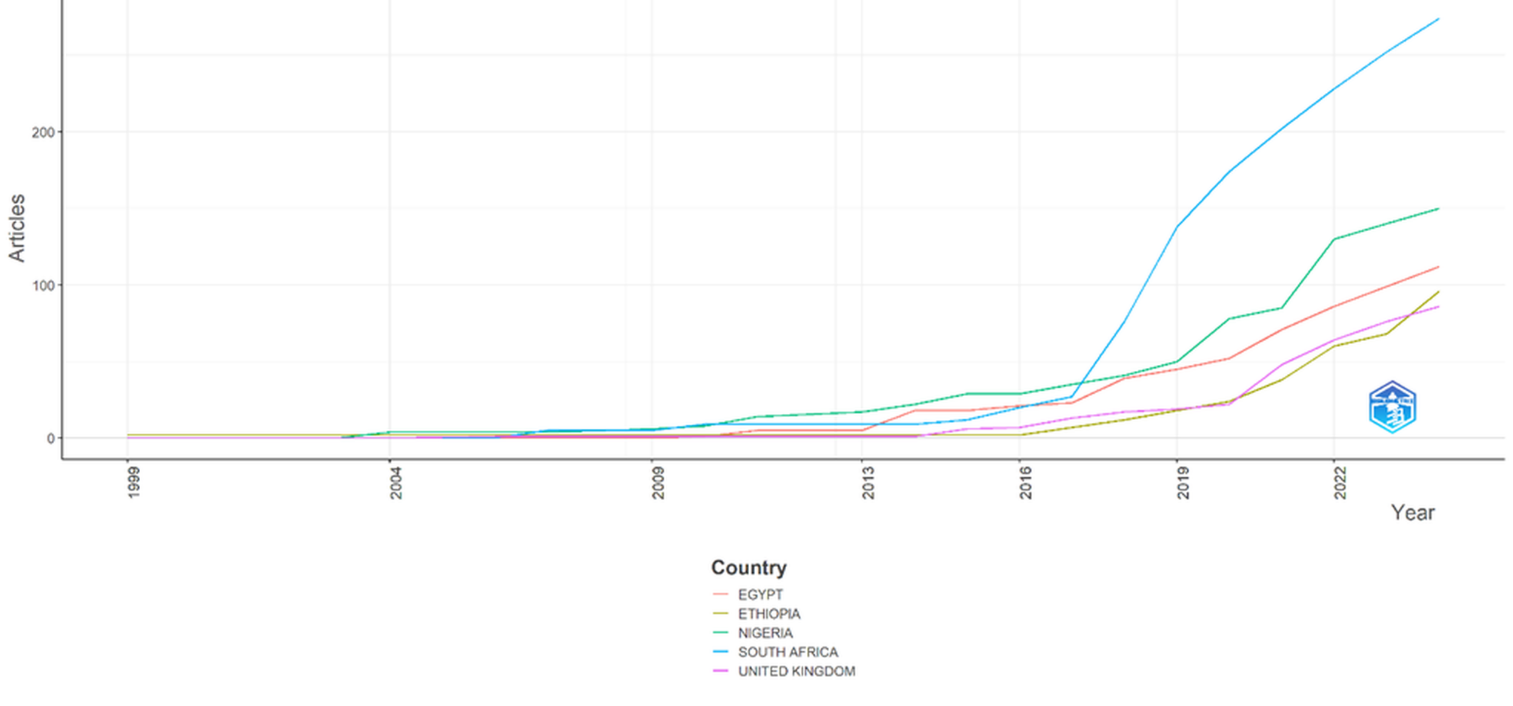


**SF 20:** Most Cited Countries in African GDM Research


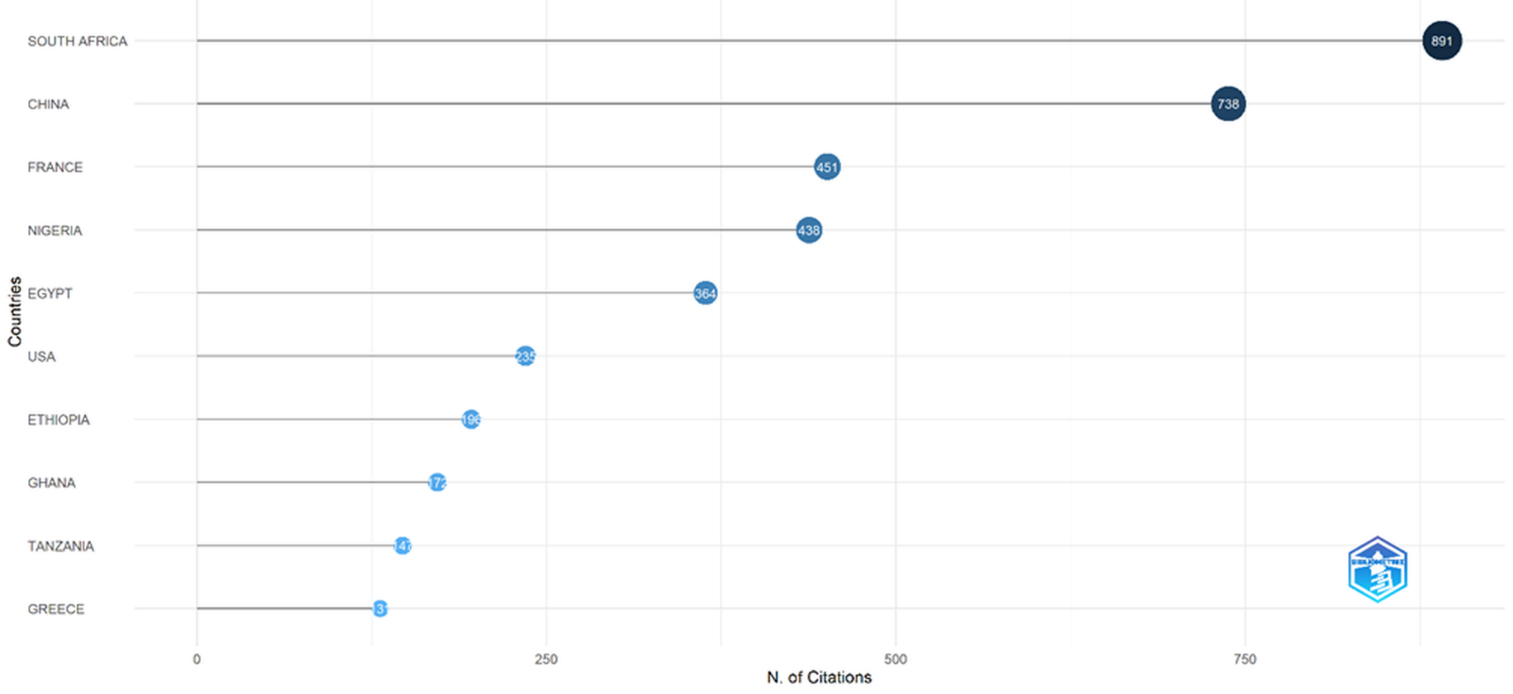


**SF 21:** Most Global Cited Documents in African GDM Research


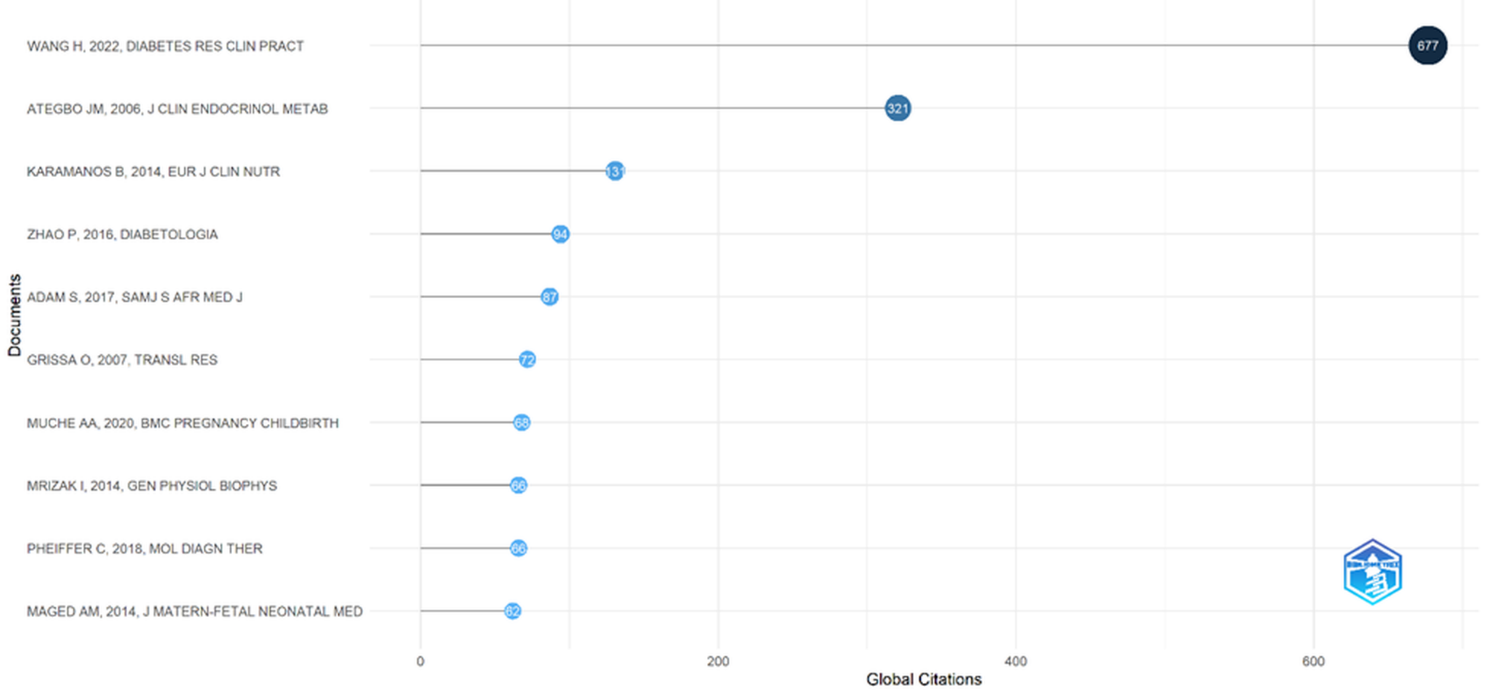


**SF 22:** Most Local Cited Documents in African GDM Research


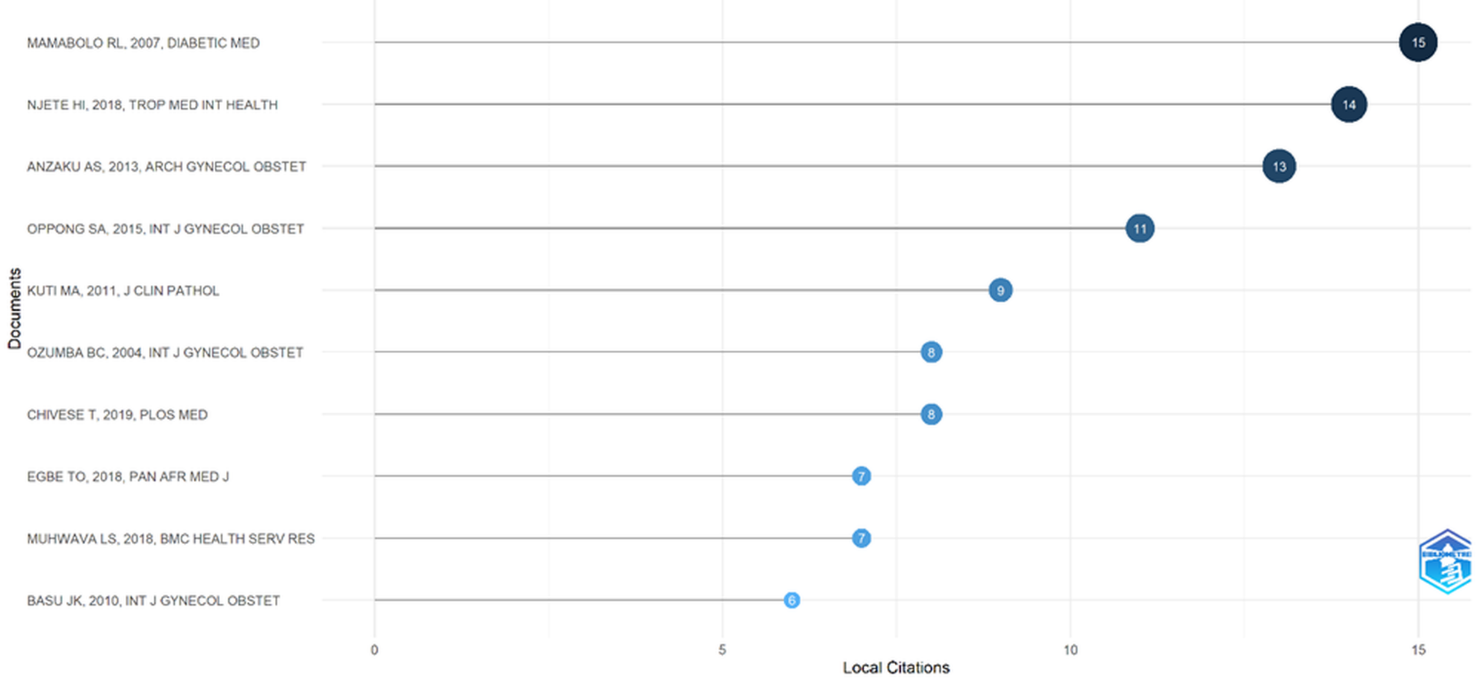


**SF 23:** Most Local Cited References in African GDM Research


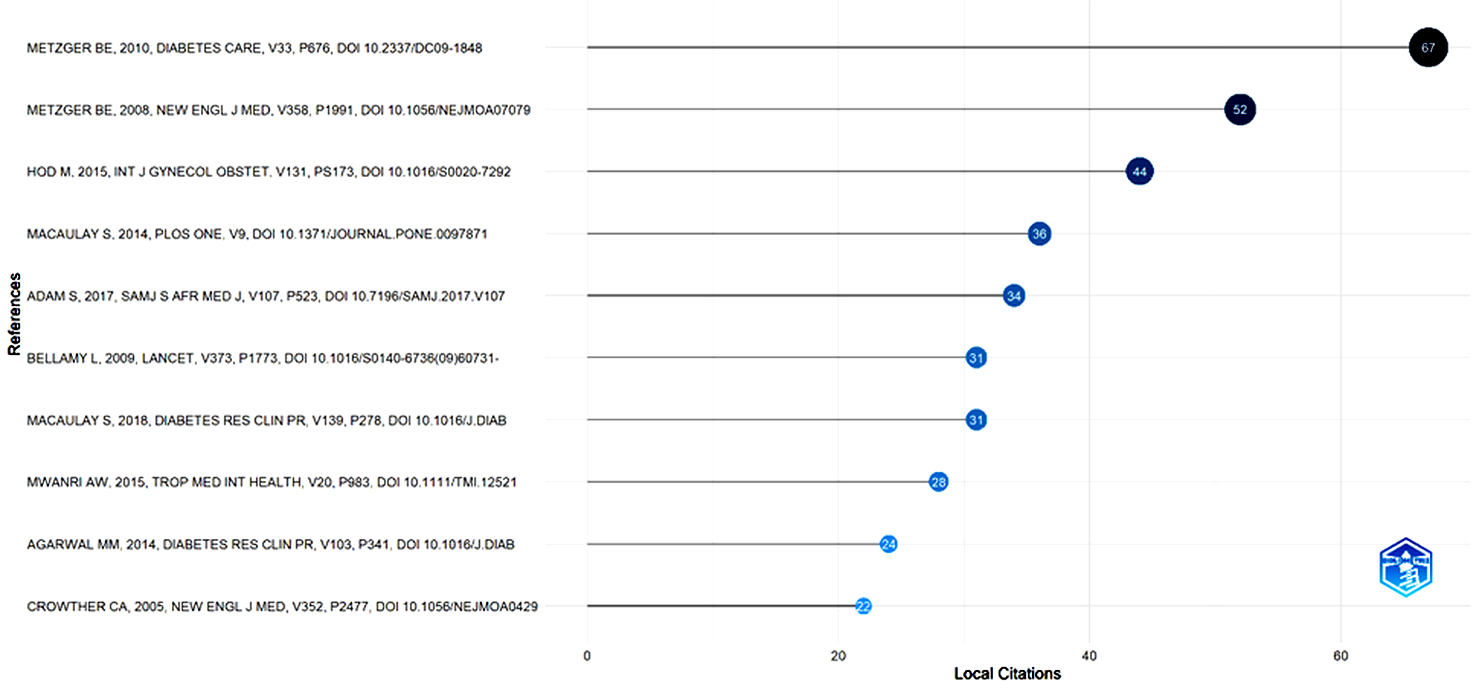


**SF 24:** Document Coupling Analysis (Clusters by Documents Coupling)


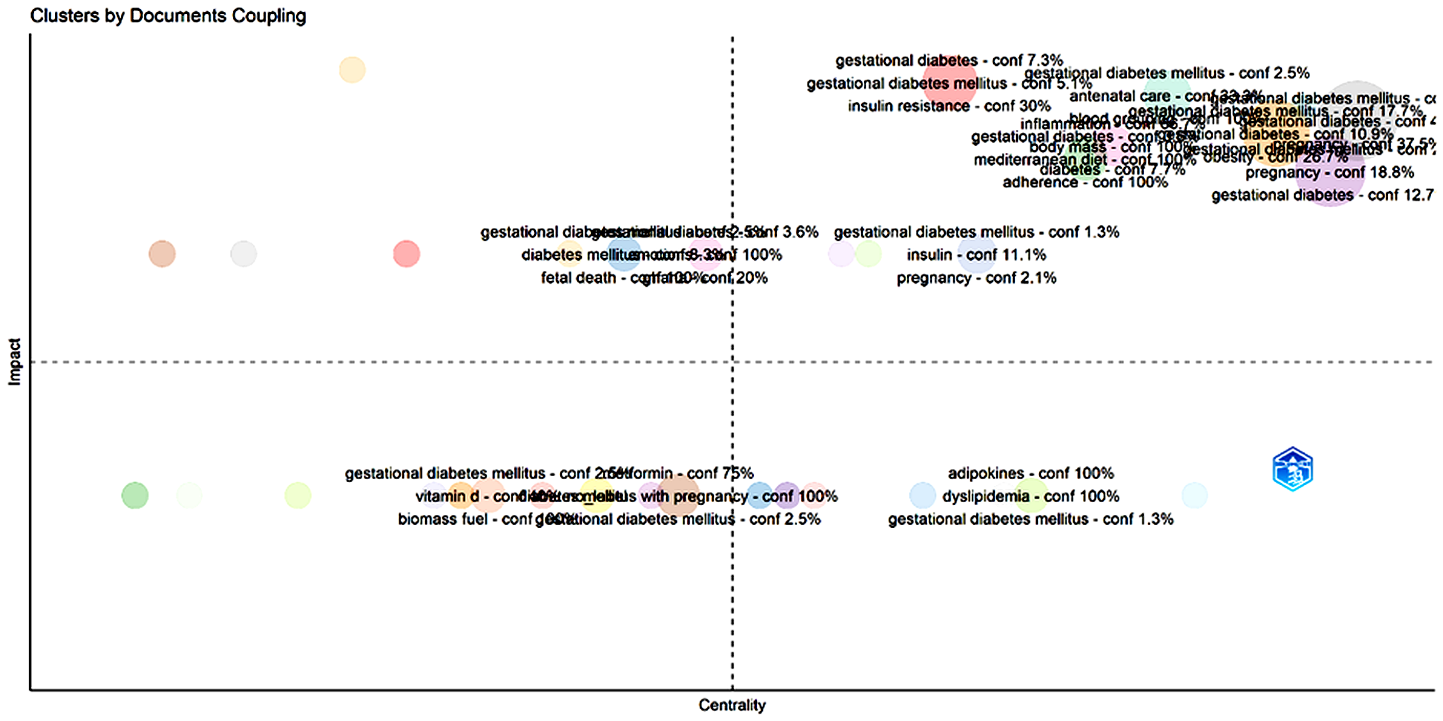


**Figure 25:** Reference Publication Year Spectroscopy (Evolution of Scientific Influence)


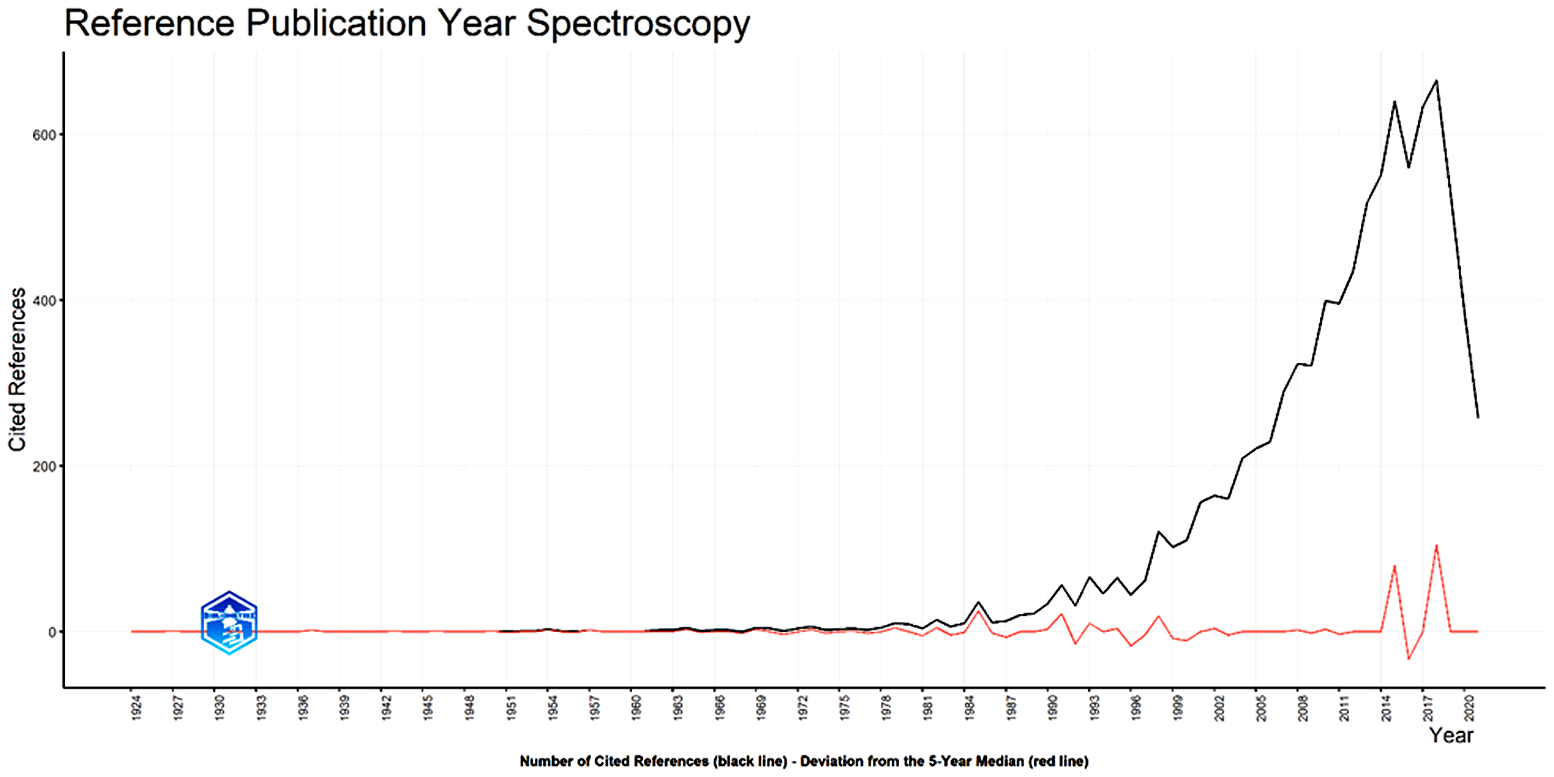


**SF 26:** Words’ Frequency Over Time in African GDM Research


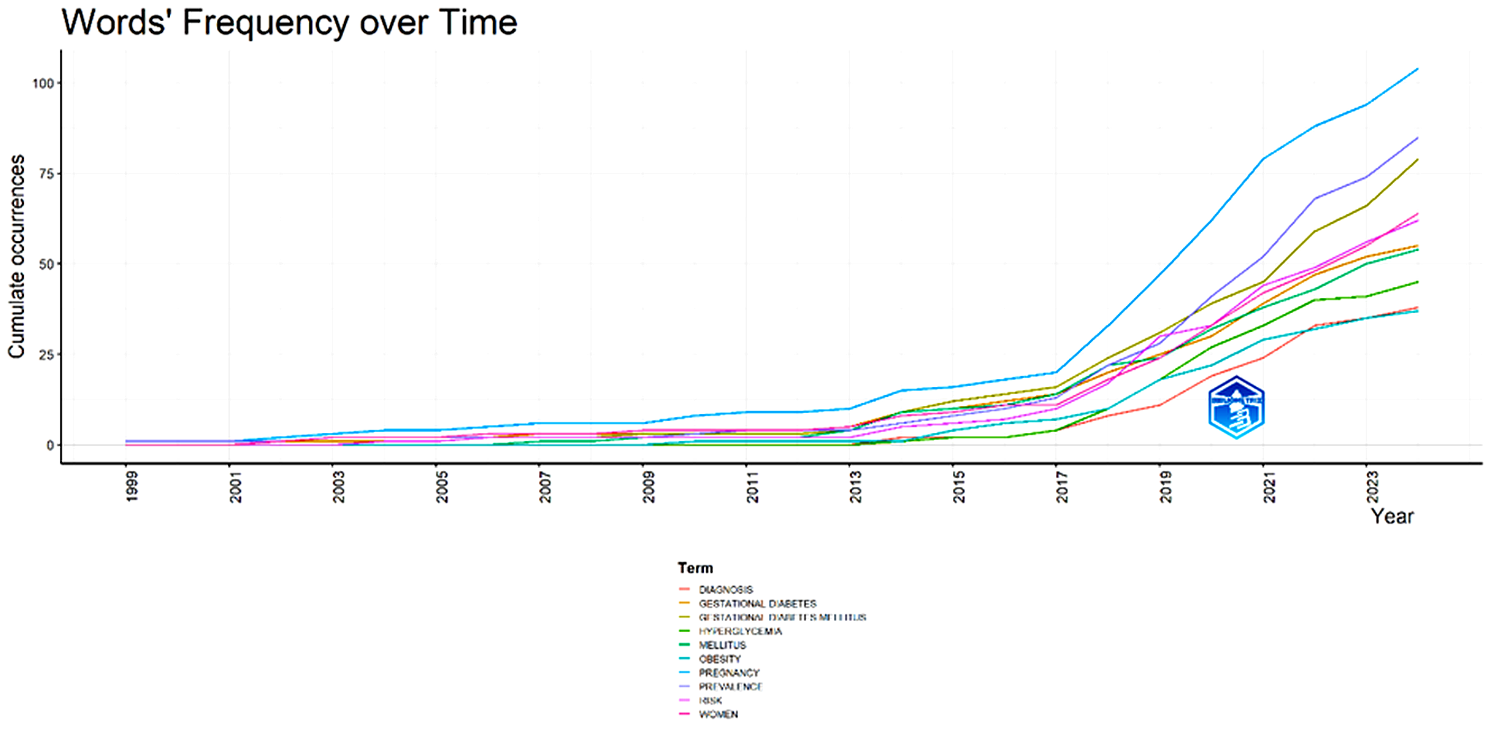


**SF 27:** Most Relevant Keywords in African GDM Research


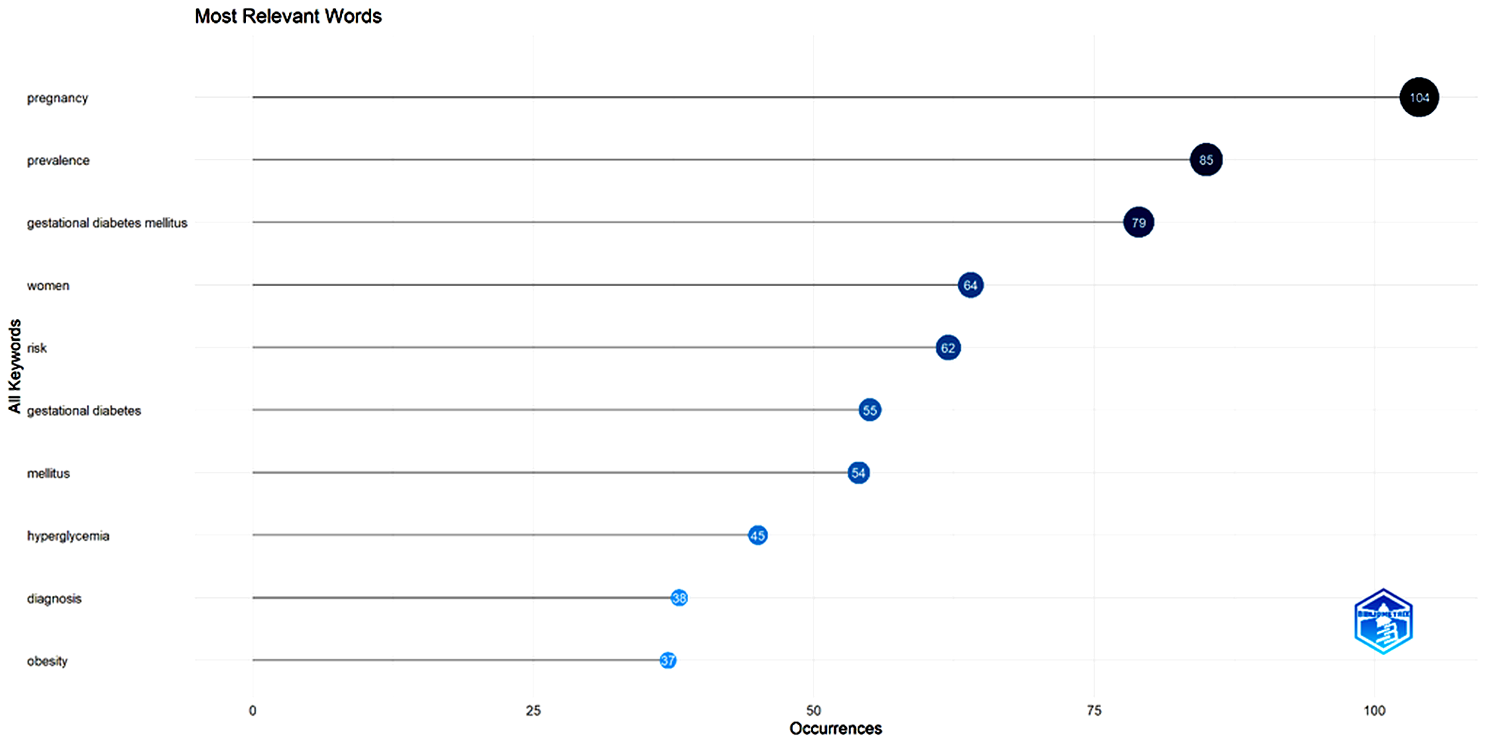


**SF 28:** Trend Topics in African GDM Research


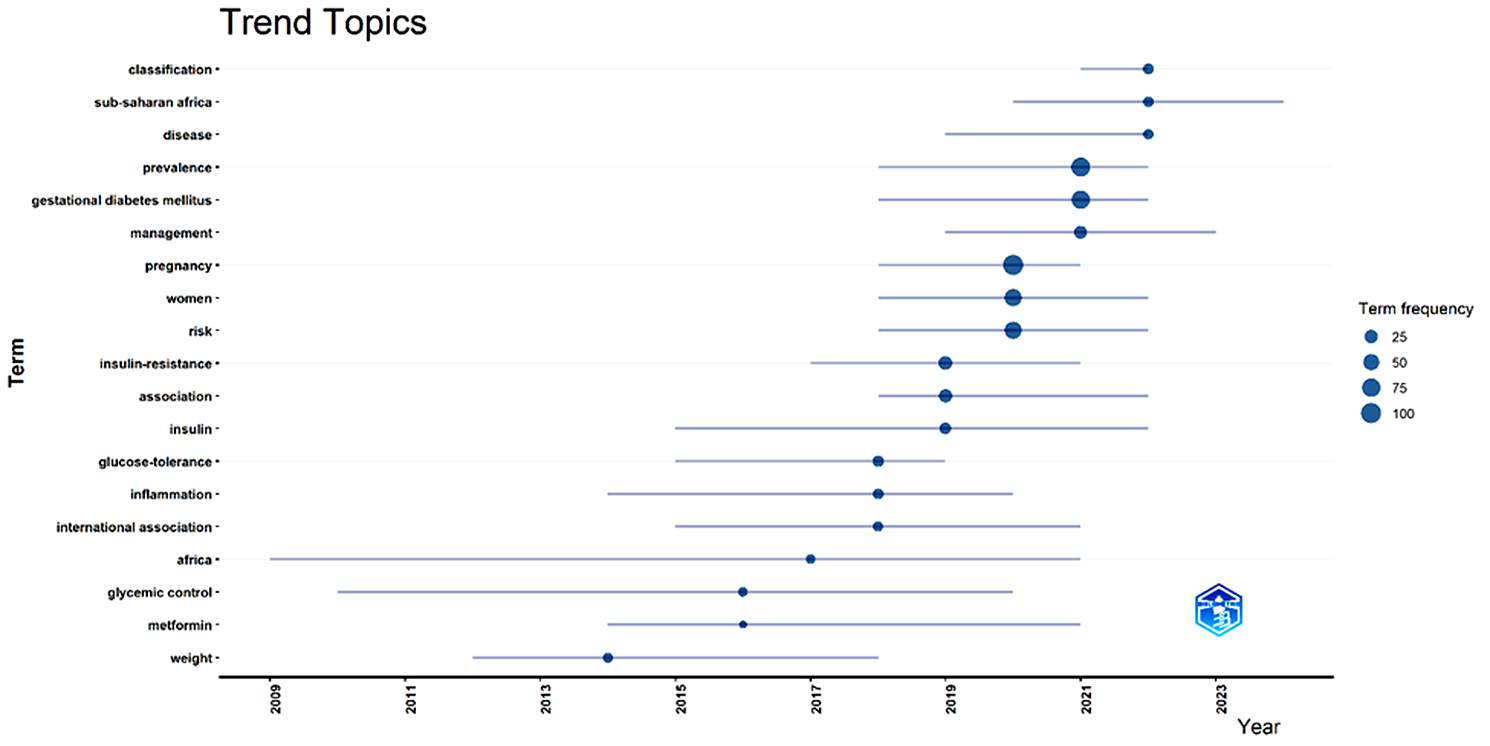


**SF 29:** GDM Treemap in African Research


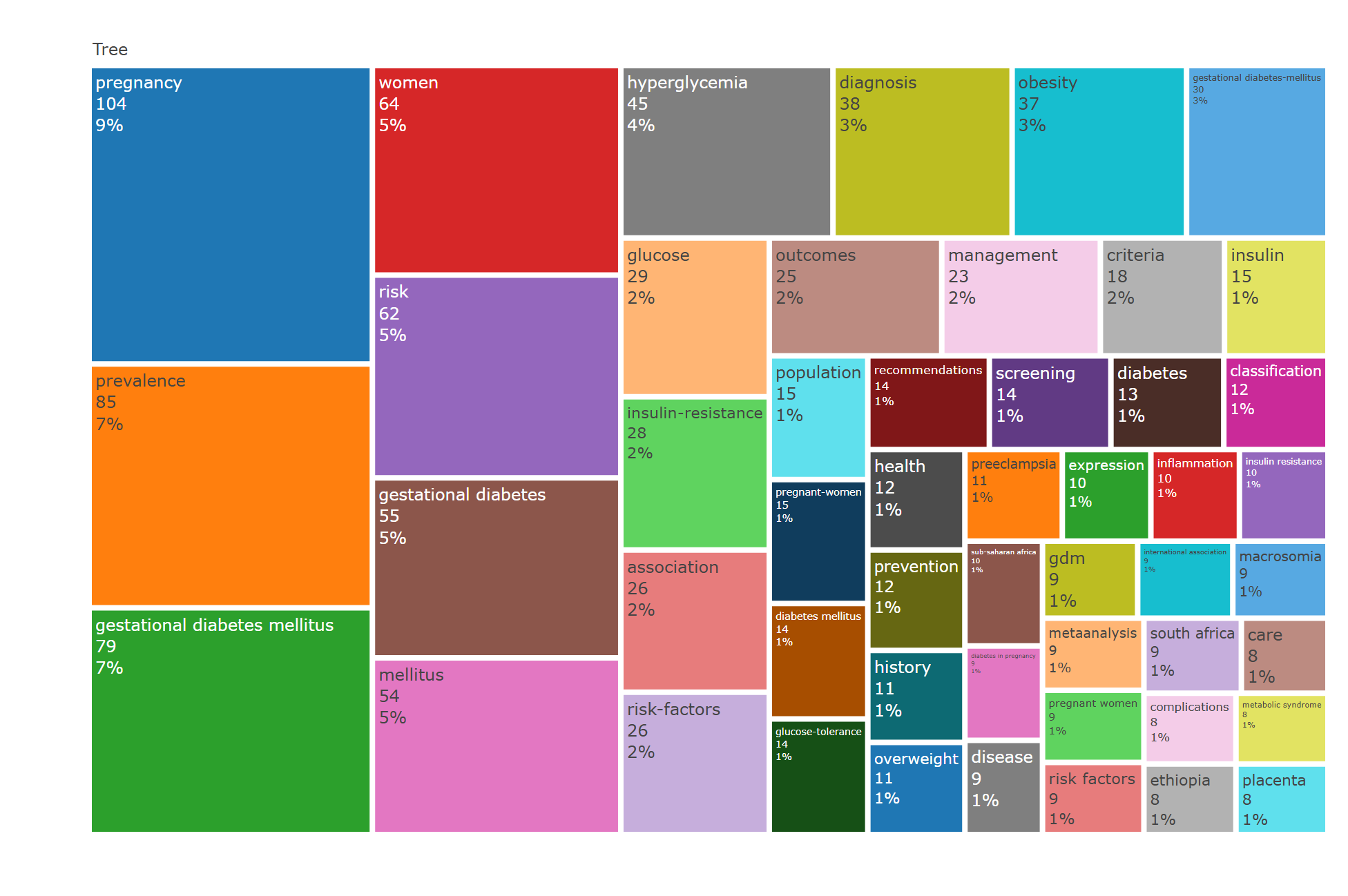


**SF 30:** Hierarchical Clustering Dendrogram in African GDM Research


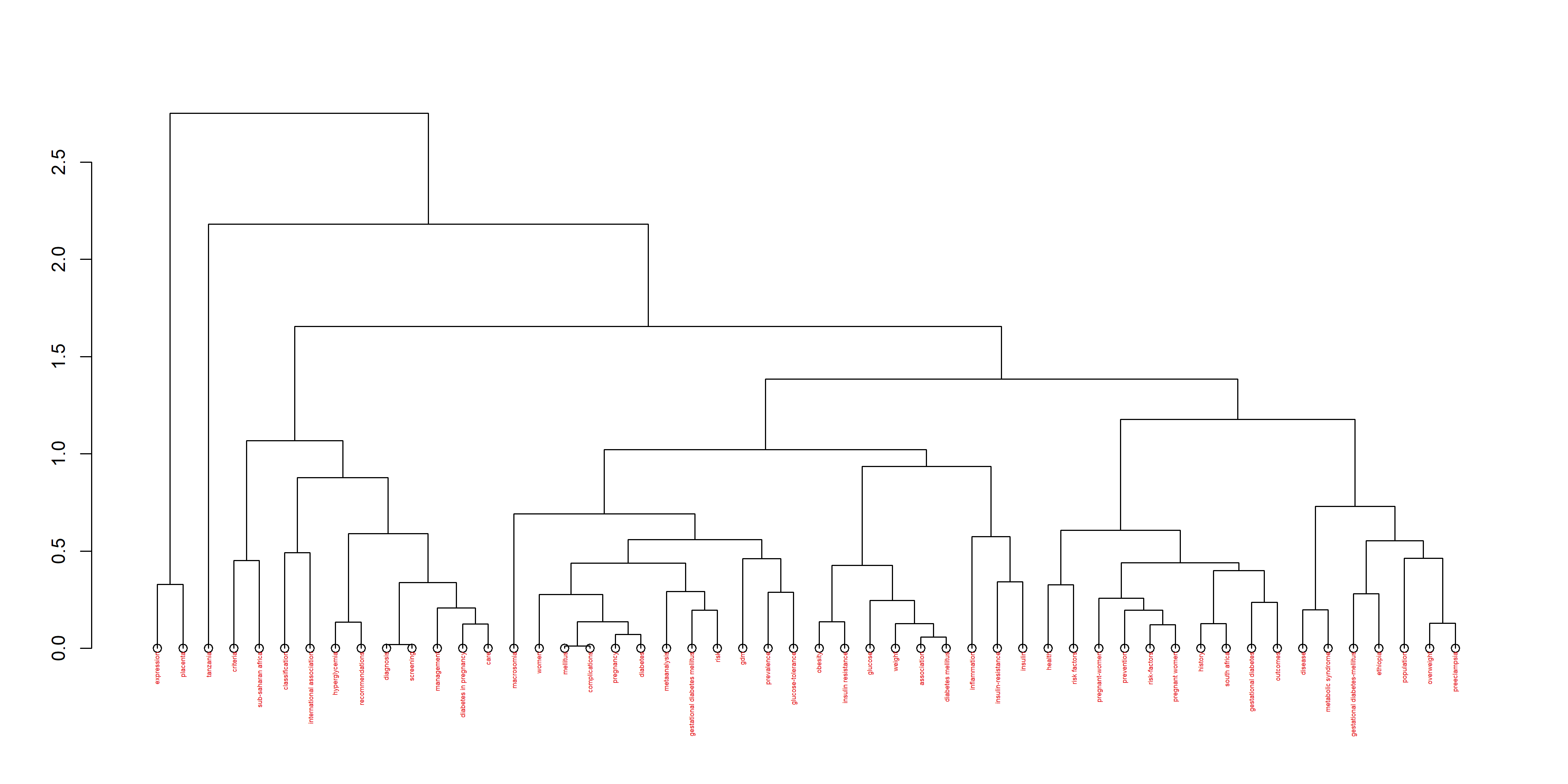


**SF 31:** Co-citation Network of GDM Research in Africa


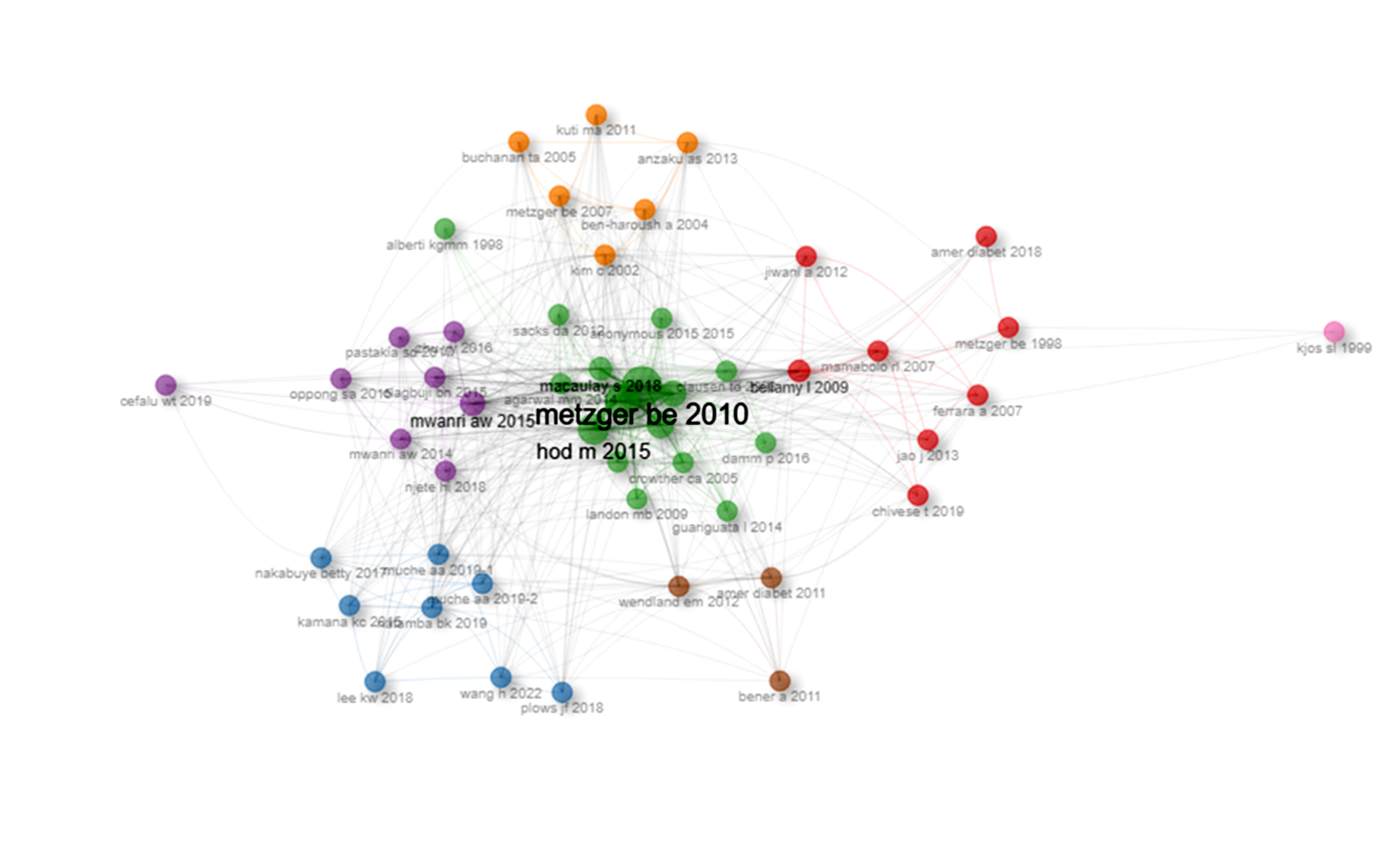


**SF 32:** Historiographic Map of GDM Research in Africa


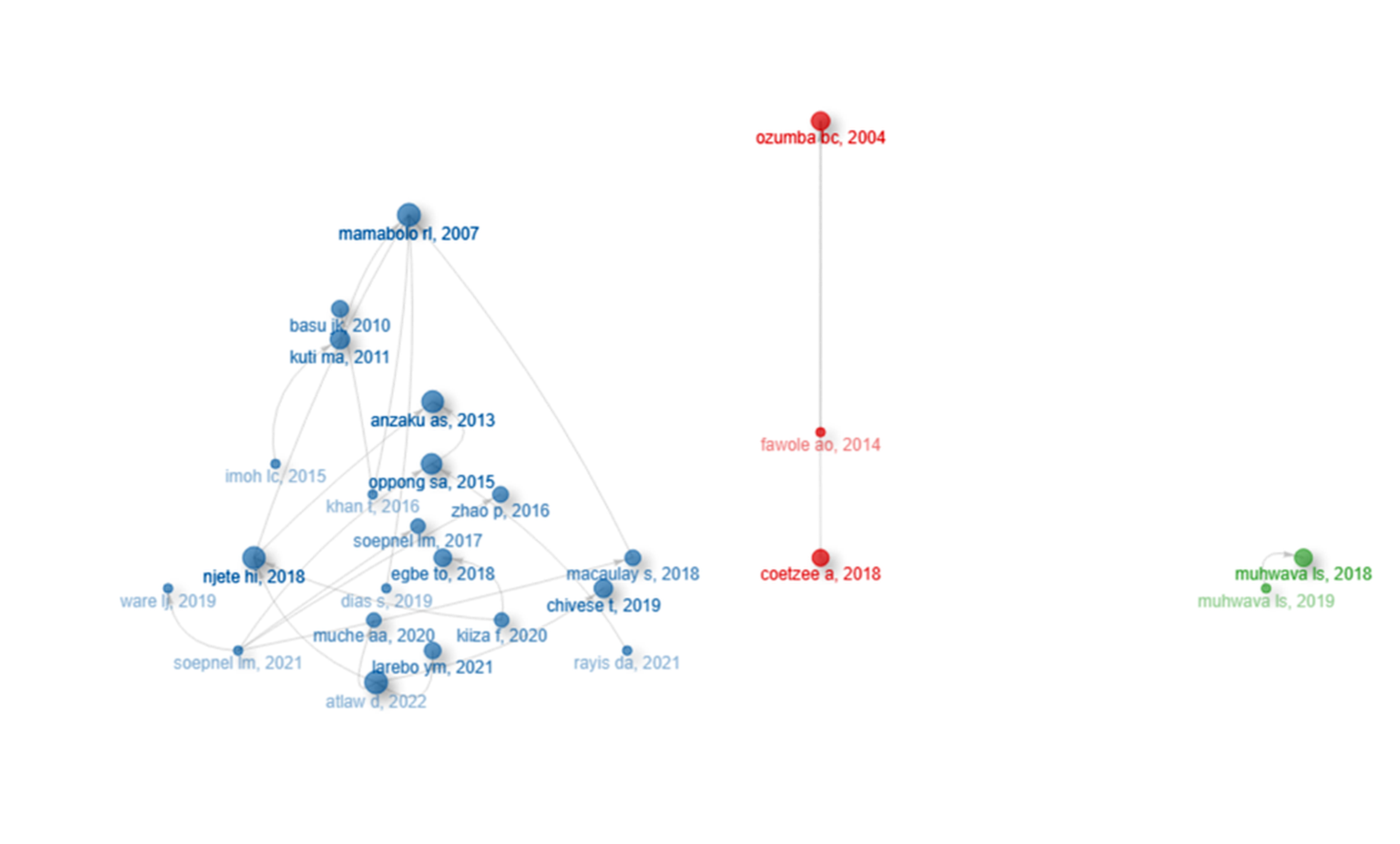


**SF 33:** Authors’ Collaboration Network of GDM Research in Africa


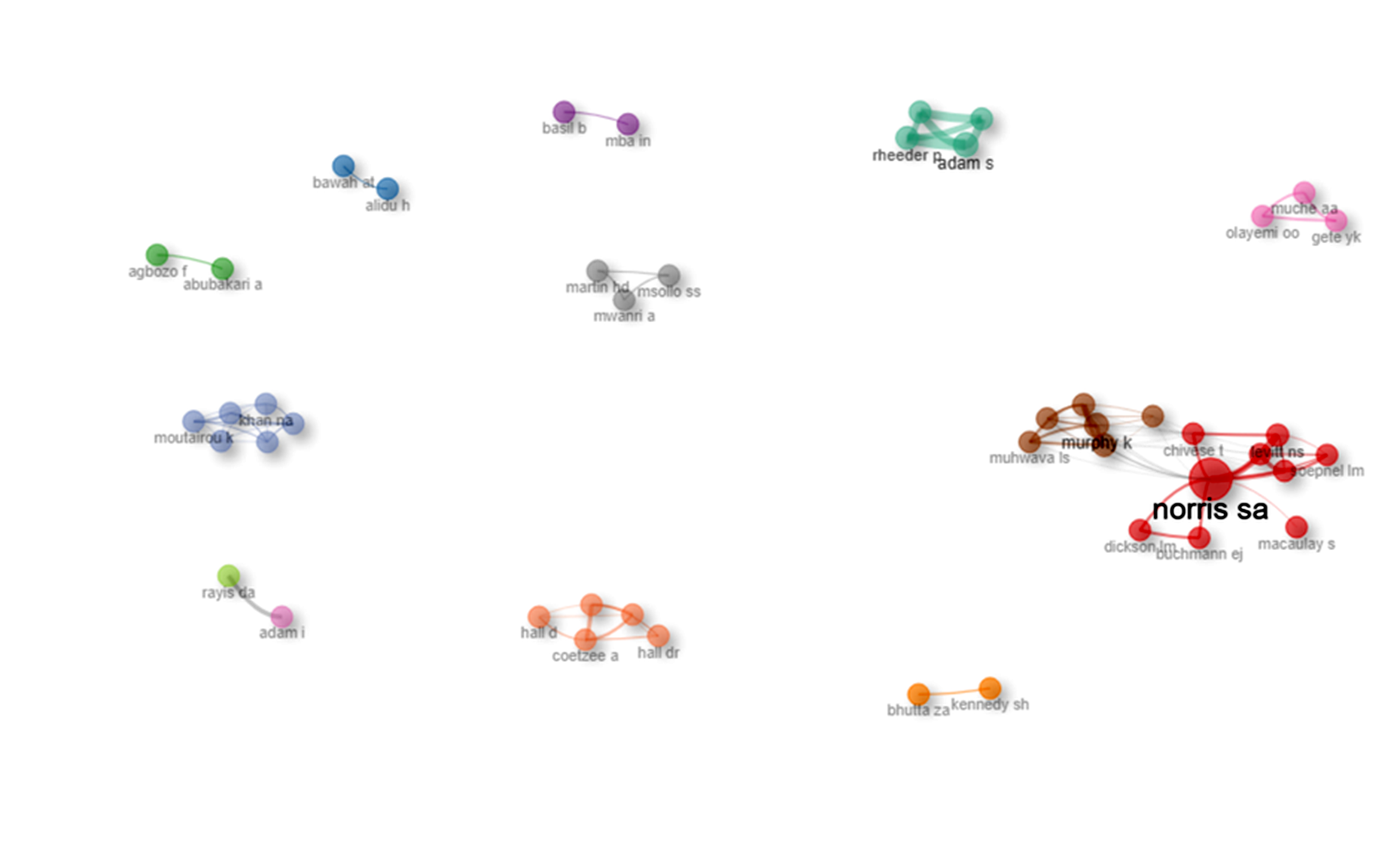


**SF 34:** GDM Word Cloud in African Research


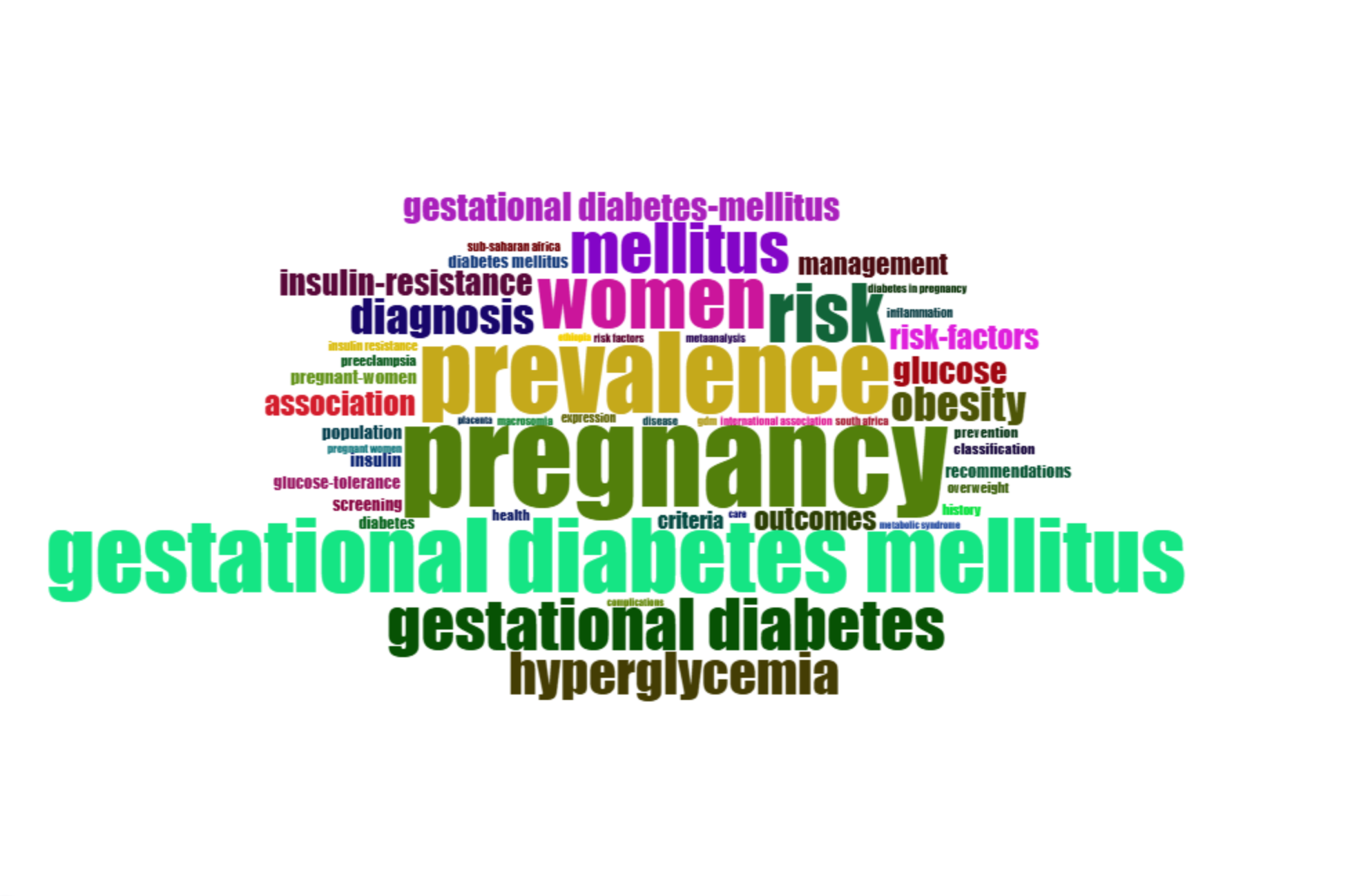


## Supplementary Table (ST)

**ST1:** Quality Assessment of Included Studies

| **Study Design** | **Appraisal Tool Used** | **Number of Studies (n=241)** | **Score Range (Min–Max)** | **Median Score** | **Overall Quality Interpretation** |
| --- | --- | --- | --- | --- | --- |
| **Cohort Studies** | Newcastle-Ottawa Scale (NOS) | 42 | 4-9 | 7 | Moderate to High Quality; most lacked full confounder adjustment |
| **Case-Control Studies** | Newcastle-Ottawa Scale (NOS) | 15 | 3-8 | 6 | Moderate Quality; selection bias common |
| **Cross-Sectional / Prevalence Studies** | JBI Checklist for Analytical Cross-Sectional Studies | 156 | 3-8 | 6 | Moderate Quality; hospital-based sampling, non-standardized diagnostics |
| **Quasi-Experimental / Interventions** | Cochrane RoB Tool | 8 | 3-7 | 5 | Moderate Risk of Bias; lack of blinding common |
| **Insufficient Methodological Detail / Design Unclear** | JBI (conservative assessment) | 20 | 2-6 | 4 | Low to Moderate Quality; reporting limitations hindered scoring |
